# Silylated cyclopentadienes as competent silicon Lewis acid catalysts†
†Electronic supplementary information (ESI) available. See DOI: 10.1039/c8sc02279h


**DOI:** 10.1039/c8sc02279h

**Published:** 2018-06-29

**Authors:** M. Alex Radtke, Tristan H. Lambert

**Affiliations:** a Department of Chemistry , Columbia University , New York , NY 10027 , USA; b Department of Chemistry and Chemical Biology , Cornell University , Ithaca , NY 14853 , USA . Email: Tristan.lambert@cornell.edu

## Abstract

The synthesis and characterization of silicon Lewis acid complexes that incorporate highly electron-deficient cyclopentadienes is reported. Several pentacarboxycyclopentadienyl and monocarboxytetracyanocyclopentadienyl complexes were prepared. A comparison of their reactivities for catalysis of the allylation of an electron-deficient benzaldehyde was established. The use of a monocarboxytetracyano silylium donor was shown to be effective for the allylation or arylation of a variety of electrophiles *via* an anion abstraction pathway.

## Abstract 2

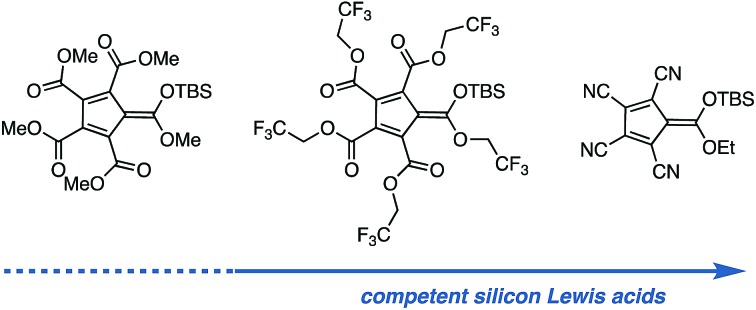
Silicon Lewis acid donor catalysts incorporating highly electron-deficient cyclopentadienes are shown to catalyze C–C bond formation *via* anion abstraction.

## Introduction

Silicon Lewis acids^1^ have proven to be useful for a variety of catalytic transformations involving the generation of highly reactive intermediates.^2^ To take advantage of this potent Lewis acidity, the silicon center must be paired with a highly stabilized conjugate base (*e.g.* triflate or triflimide),^3^ and a number of such reagents are commercially available. However, many applications of silicon Lewis acid catalysis require the ability to modulate the properties of the anionic leaving group (*e.g.* stability, solubility, and chirality) beyond what these simple species allow. To this end, notable advances have been made in the development of effective chiral anions,^4^ extremely stable anions such as carboranes^5^ and perfluoroborates,^6^ and complex counterions generated *via* anion binding.^7^ Despite these important advances, there remains an important need for new, highly stable, readily accessible, and broadly diversifiable anion frameworks.

**Fig. 1:**
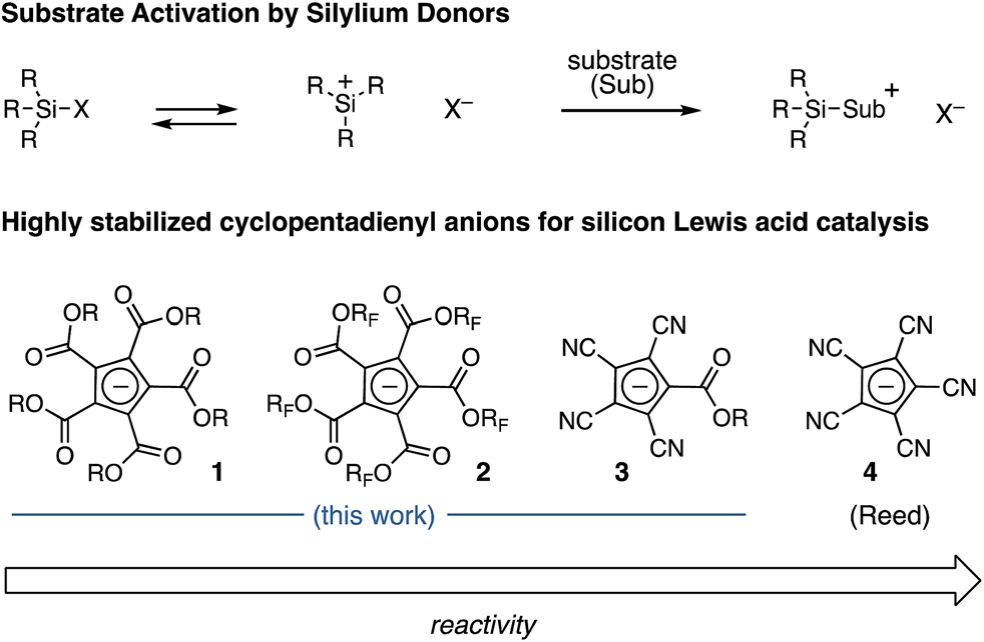
Generic scheme for silicon Lewis acid activation and the structures of representative stabilized cyclopentadienyl anions.

We have been exploring the development of electron-deficient cyclopentadienes (CPs) for applications in catalysis.^8^ These ions are attractive due to their straightforward synthesis, broad potential for structural modification, and capacity to enable very high levels of anion stability.8a,^9^ This stability suggests that the ions might have utility for silicon Lewis acid catalysis, and in fact, Reed has reported the preparation of silyl complexes of pentacyanocyclopentadiene **4** (Fig. 1).^10^ Unfortunately, **4** offers no handles for modification, which would be necessary for broad development of these materials as silicon Lewis acid catalysts. The development of CP-based silicon Lewis acids with alternative functionalities, such as carboxyl groups (**1**), could prove useful; however, the viability of these less-stabilized anions for Lewis acid catalysis has not been demonstrated. We speculated that silyl complexes of other electron-deficient cyclopentadienyl anions, such as fluorinated pentacarboxy cyclopentadienes (PCCPs) (**2**) or the mixed cyano/carboxy cyclopentadienes (**3**) developed by Mori,^11^ could display useful levels of Lewis acidic character while providing functional handles to modify attributes such as solubility or chirality. In this communication, we validate this hypothesis with the synthesis of several such materials and their application to catalytic C–C bond forming reactions.

## Results and discussion

**Fig. 2:**
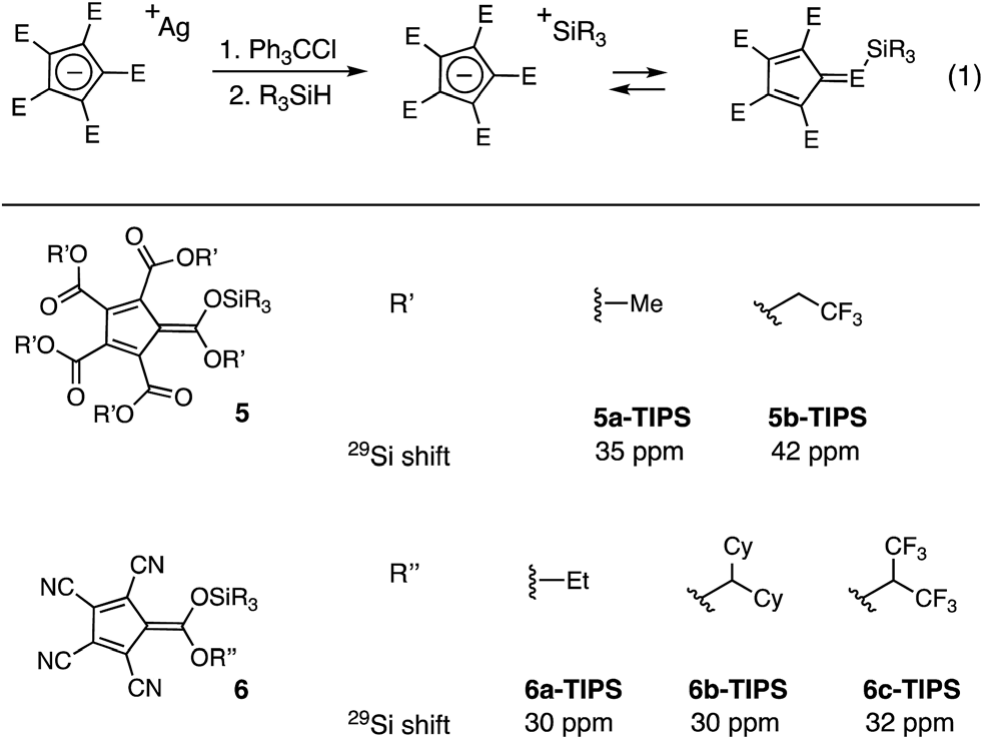
Synthesis and characterization of silylated cyclopentadiene complexes.

The silylated derivatives of a series of cyclopentadienyl anions were prepared according to Reed's procedure for the synthesis of **4** (Fig. 2, eqn (1)).^10^ The silver salts were obtained by cation metathesis from the Na^+^ or NMe_4_^+^ salts (see ESI†). Treatment of the silver salts with trityl chloride followed by triisopropylsilane or *tert*-butyldimethylsilane furnished the silylated CPs, which were characterized by ^29^Si-NMR. The ^29^Si shift of methyl PCCP **5a** occurs at *δ* 35 ppm, whereas the trifluoroethyl PCCP (**5b**) is appreciably downfield at *δ* 42 ppm, comparable to iPr_3_SiOTf (*δ* 40 ppm). By comparison, the tetracyano CPs (**6a–c**) have a markedly upfield shift (∼*δ* 30 ppm), although the nature of the ester substituent has minimal impact. These lower shifts for what are unequivocably more stable anions could be reflective of the smaller steric bulk of the flanking cyano groups in comparison to the carboxy groups of the PCCP, which allows for greater coordination to the electropositive silicon. Alternatively, the lower shifts might mean that the silyl group is bonded *via* one of the nitrogen atoms. Nevertheless, the tetracyano CP (**6a**) does display increased reactivity (*vide infra*), as expected based on consideration of anion stabilities.

**Fig. 3:**
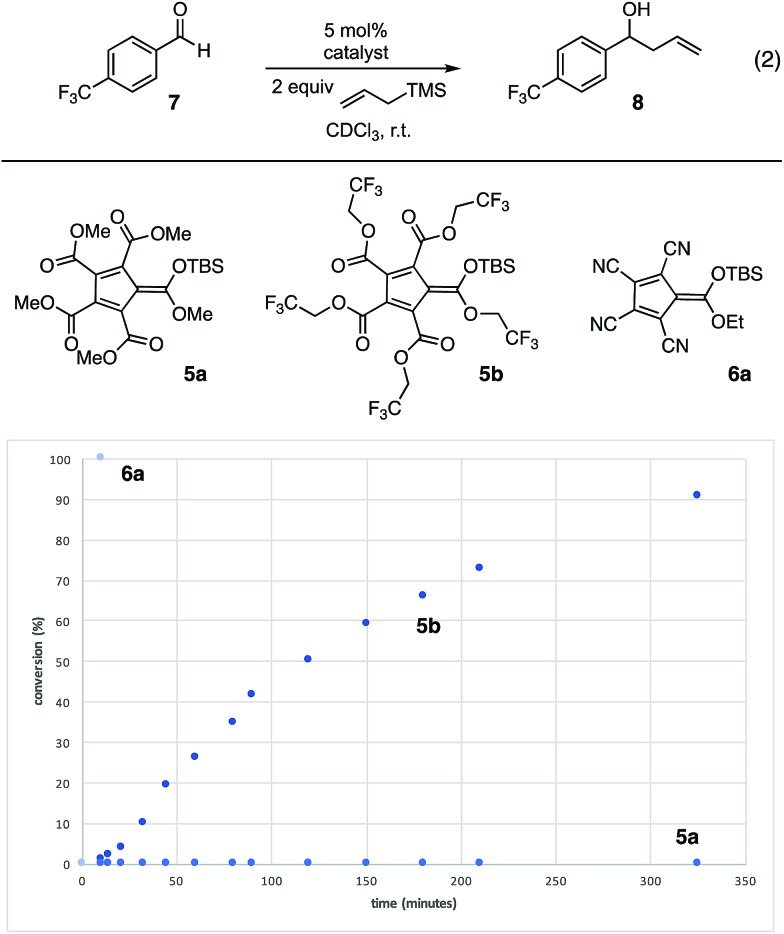
Comparison of silicon Lewis acid reactivity. Reaction conditions: **7** (0.1 mmol), allyltrimethylsilane (0.2 mmol), catalyst (0.005 mmol), 800 μL CDCl_3_, % conversion by ^1^H-NMR.

To compare the reactivities of the silicon Lewis acids, we examined the catalytic allylation of 4-trifluoromethyl-benzaldehyde (**7**) (eqn (2)).^12^ As shown in Fig. 3, no allylation was observed in the presence of catalytic **5a**, even after an extended time period. This lack of reactivity can be attributed to the poor Lewis basicity of **5a**; given that the corresponding acid of **5a** has an acidity comparable to HCl, this result is not surprising.‡
‡In these reactions, the anion may also be serving as a Lewis base activator for the allyltrimethylsilane. In support of this idea, we found that TMSOTf did not catalyze this reaction. Thus, we speculate that the cyclopentadienyl anions provide sufficient Lewis acid and Lewis base character for activation of both electrophile and nucleophile in this process. In contrast, **5b**, bearing electron-withdrawing trifluoroethyl substituents, did catalyze allylation, reaching 90% conversion in less than 6 h at 5 mol% loading. To further increase reactivity, we turned our attention to the monocarboxytetracyano CPs (**6**).^11^ The most reactive of these, **6a**, catalyzed full conversion of **7** to **8** in under five minutes. Given this potent reactivity, the ease of synthesis of the cyclopentadienyl precursor, and the fact that the carboxy substituent of **6a** retains a functional handle with which one might modulate the functional characteristics of the scaffold, we decided to further probe the potential applications of this catalyst.

**Fig. 4:**
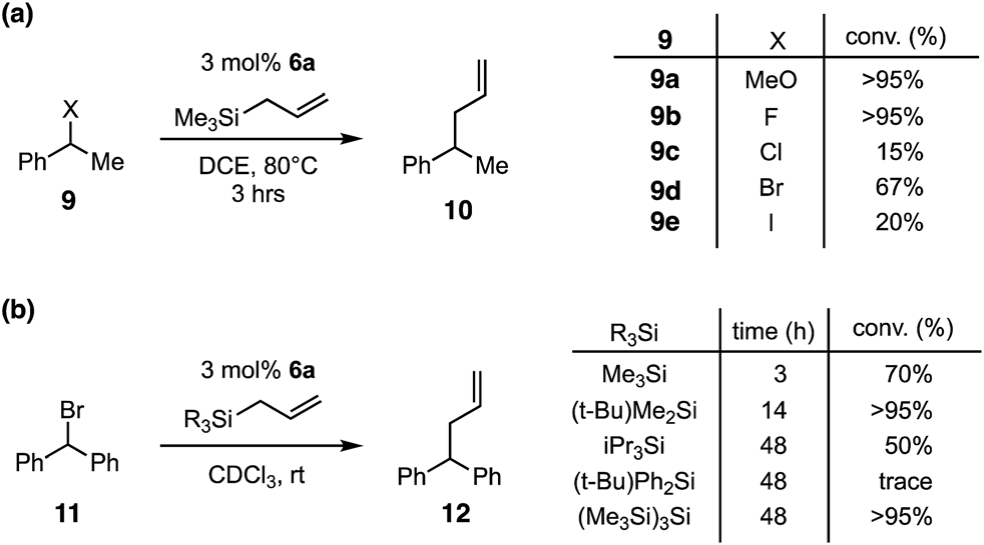
(a) Silyl-CP-catalyzed halide abstraction. Reaction conditions: **9** (0.1 mmol), allyltrimethylsilane (0.2 mmol), **6a** (0.003 mmol), 1 mL DCE, r.t., % conversion by ^1^H-NMR. (b) Impact of allylsilane on reaction with benzhydryl bromide. Reaction conditions: **11** (0.1 mmol), allylsilane (0.2 mmol), **6a** (0.003 mmol), 600 μL CDCl_3_, r.t., % conversion by ^1^H-NMR.

In light of the high electrophilicity of **6a**, we anticipated that other silicon Lewis acid reactivities, such as halide abstraction, might be possible.^13^ Indeed, as shown in Fig. 4a, we found that the formation of phenylethyl cations and subsequent trapping with allyltrimethylsilane could be achieved with a variety of pro-electrophiles,^14^ including methyl ether and any of the halides. The most productive substrates were the ether and the fluoride, which is consistent with expectations of silylium-induced nucleofugacity. Interestingly, the bromide was a moderately effective substrate, while both the chloride and iodide resulted in only low conversions after 3 h.

We next examined the impact of the allylsilane moiety (Fig. 4b) on the reaction with benzhydryl bromide. In this case, an inverse correlation of steric demand and reactivity was observed, with trimethylsilyl reacting the fastest while bulkier groups, such as triisopropylsilyl and *tert*-butyldiphenylsilyl, reacted significantly more slowly or not at all. Given that the silyl group of the allyl donor becomes the catalytic silyl species after one turnover, we believe that the lower reactivity of the larger silyl reagents is due to a decrease in Lewis acidity owing to steric encumbrance. On the other hand, the larger allylsilanes would also be expected to be less reactive, and thus we cannot definitely attribute the cause of the rate decrease. It should be noted that, although it was more reactive, the trimethylsilyl catalyst seemed to exhibit a greater propensity towards decomposition, leading to incomplete conversion. In contrast, the *tert*-butyldimethylsilyl reagent displayed intermediate reactivity but produced a higher level of conversion, underscoring the notion that there is a balance to be struck between Lewis acidity and stability.

We next probed the substrate scope of the **6a**-catalyzed benzylic allylation under our optimized conditions (Table 1). A series of benzhydryl substrates, ranging from electron-rich to electron-deficient, were allylated in good to high yields (entries 1–6). In the case of the more difficult to ionize p-CF_3_ substrate, lower conversion was observed even at elevated temperatures and the yield was significantly diminished (entry 7). Notably, monobenzylic bromide substrates were also found to be viable with an increase in reaction temperature to 80 °C (entries 8–14). In the reaction of cyclic substrates, ring size had minimal impact on efficiency (entries 12–14). Finally, while tertiary alkyl bromides were generally unreactive, adamantyl bromide underwent allylation to give a 2 : 1 mixture of regioisomeric products, with **25** as the major isomer, albeit in modest yield (30% combined, entry 15).

**Table 1:** Silyl CP-catalyzed benzylic allylation^*a*^

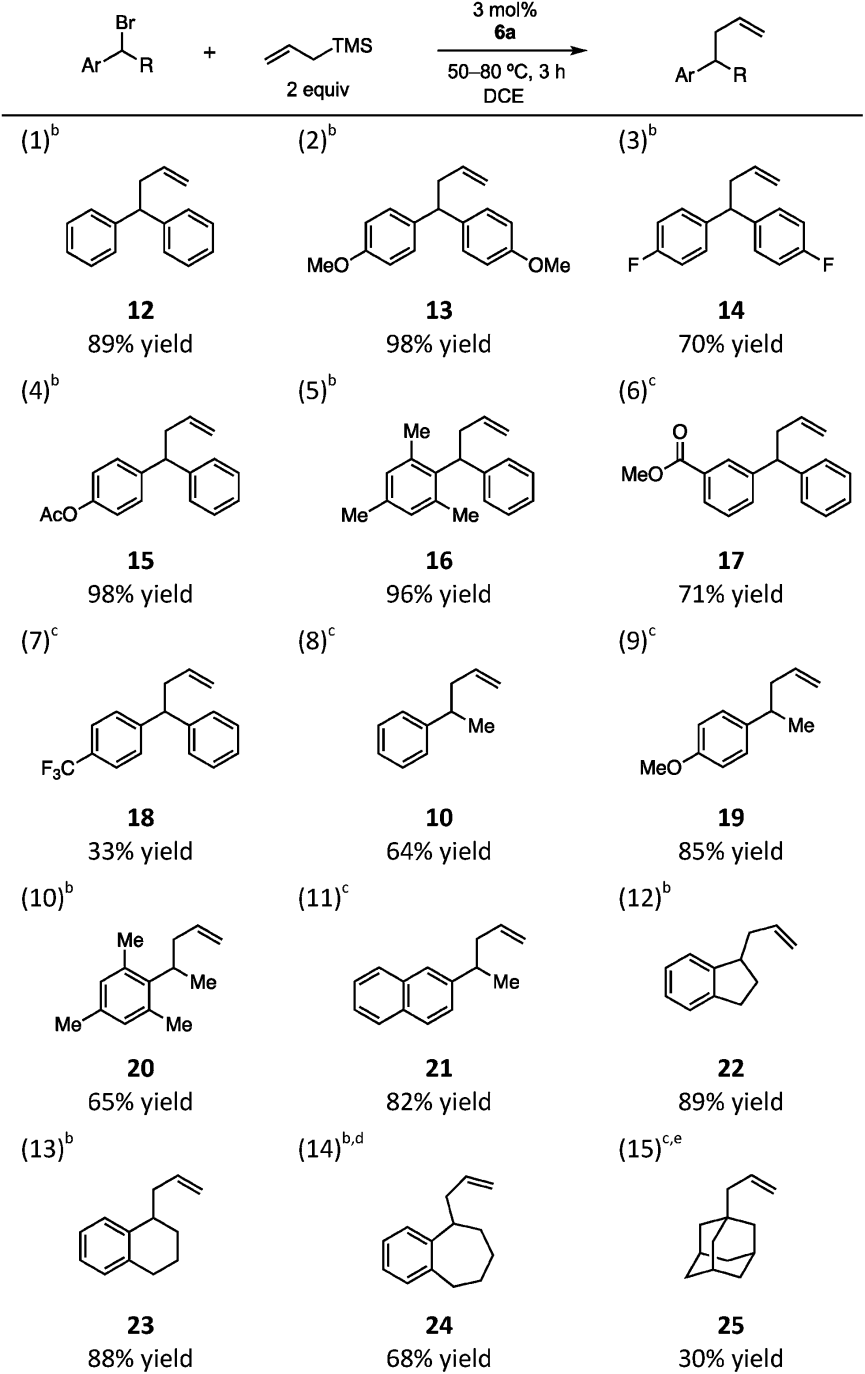

^*a*^Reactions conditions: bromide (0.1 mmol), allyltrimethylsilane (0.2 mmol), **6a** (0.003 mmol), 1 mL DCE.

^*b*^Reaction run at 50 °C.

^*c*^Reaction run at 80 °C.

^*d*^Elimination product also observed (17%).

^*e*^A 2 : 1 ratio of **25** and the internal olefin regioisomer was obtained in a combined yield of 30%; see ESI for details.

**Fig. 5:**
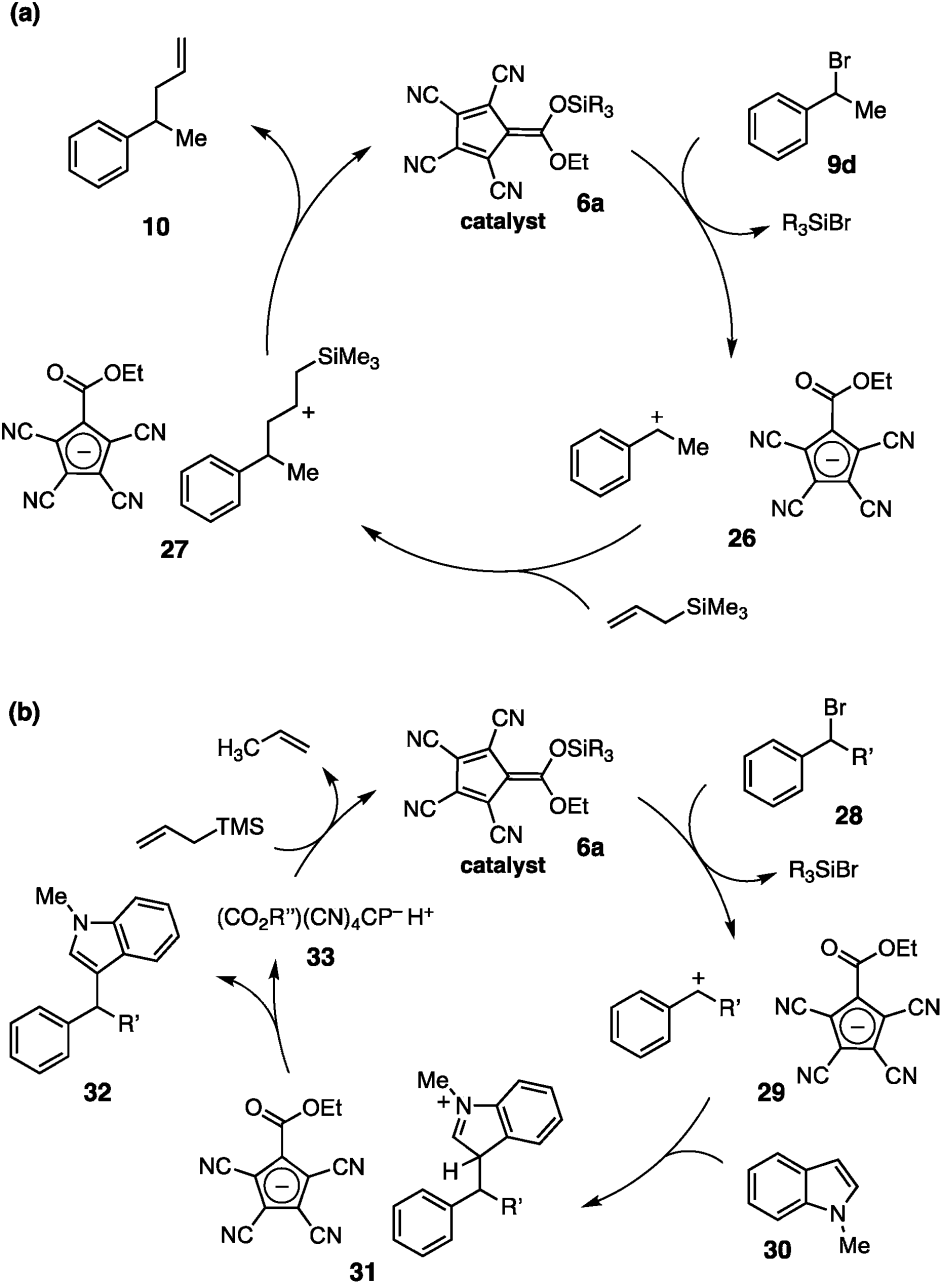
Mechanistic rationale for silyl CP-catalyzed allylation and arylation.

The mechanistic rationale for the catalytic allylation reaction is shown in Fig. 5a. Reaction of catalyst **6a** with benzylic bromide **9d** leads to ionization *via* silyl-induced halide abstraction. The resulting carbenium-cyclopentadienyl salt, **26**, then undergoes attack by the allylsilane to produce intermediate **27**. Desilylation of this species produces the allylated adduct **10** and regenerates the silyl catalyst, **6a**, completing the catalytic cycle.

Because of the potent electrophilicity of the carbocationic intermediates, we anticipate that this system should be applicable to a range of other substitutions with silylated nucleophiles. Non-silylated nucleophiles are also expected to react, but in these cases the silyl catalyst would not be regenerated after the addition step (Fig. 5b). For example, reaction with an aryl nucleophile, such as *N*-methylindole (**30**), would proceed *via* the expected pathway to afford product **32**, along with the CP acid **33**. Regeneration of the silyl catalyst, **6a**, might be achieved *via* protodesilylation of a sacrificial silyl source,5f,^15^ such as allyltrimethylsilane; however, the feasibility of this approach would require the arene (**30**) to outcompete the allylsilane for reaction with the carbocation intermediate.

Indeed, we found this sacrificial silane approach to be viable. A brief survey of the substrate scope of this process in the context of the diphenylmethylation of arenes is shown in Table 2. We found that *N*-benzyl and *N*-allyl indole reacted in high yield, as did unprotected indole, albeit in more modest yield (entries 1–3). Alkyl substitution on the indole was tolerated, as reaction of both 1,2-dimethylindole (entry 4) and 1,3-dimethylindole (entry 5) resulted in good yields of the alkylated products. A similarly productive reaction was observed with 1,3-dimethoxybenzene as the nucleophile (entry 6), although in this case an 8 : 1 mixture of mono- and dialkylated products was obtained. In terms of heteroaromatics, although furan was not reactive, 2-methylfuran was a very efficient reaction partner, leading to product in nearly quantitative yield (entry 7). *N*-Phenylpyrrole readily participated in the reaction, affording **41** in good yield as a 4 : 1 mixture of the 2- and 3-substituted pyrroles (entry 8). Finally, reaction of 1,2,5-trimethylpyrrole led to the alkylated product **42**, though in modest yield (entry 9). Our scope studies did reveal some limitations to the transformation: substrates bearing electron-withdrawing substitutents (Ac, Ts) on the nitrogen are unreactive (not shown). Moreover, anisole is insufficiently nucleophilic to outcompete the allylsilane.^16^

**Table 2:** Silyl CP-catalyzed Friedel–Crafts alkylation^*a*^

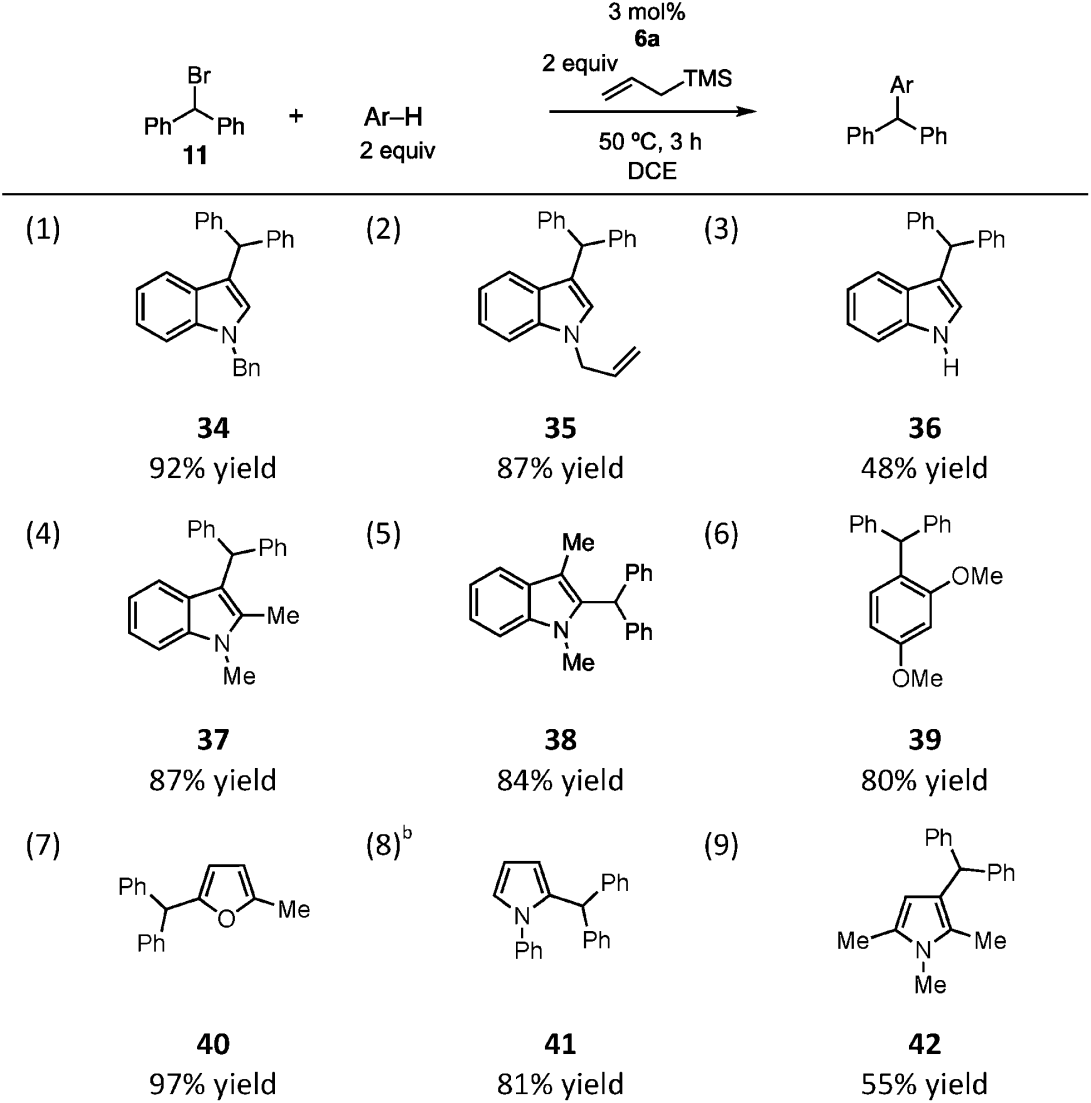

^*a*^Reaction conditions: **11** (0.1 mmol), allyltrimethylsilane (0.2 mmol), arene (0.2 mmol), **6a** (0.003 mmol), 1 mL DCE.

^*b*^Isolated as a 4 : 1 mixture of 2- and 3-benzhydrylpyrroles.

## Conclusions

In conclusion, we have demonstrated that silylated electron-deficient CPs, including pentacarboxycyclo pentadienes (**5**) and monocarboxytetracyanocyclopentadienes (**6**), can serve as effective silicon Lewis acid catalysts. The latter in particular was found to offer a favorable balance between reactivity and solubility. Importantly, the carboxy group retains the potential for diversification of the cyclopentadienyl framework, which we expect may prove useful for a variety of applications.

## Conflicts of interest

There are no conflicts to declare.

## Supplementary Material

Supplementary information
