# Supplementary material for: Silylated cyclopentadienes as competent silicon Lewis acid catalysts
Source: Chem Sci. 2018 Jun 29;9(30):6406–10. doi: 10.1039/c8sc02279h (PMC6115689; doi:10.1039/c8sc02279h)
Supplement: Supplementary file 1 [file SC-009-C8SC02279H-s001.pdf]

Electronic Supplementary Material (ESI) for Chemical Science.
This journal is © The Royal Society of Chemistry 2018

SUPPORTING INFORMATION

Silylated Cyclopentadienes as Competent Silylium Catalysts

M. Alex Radtke[a] and Tristan H. Lambert[a,b*]

[a]Department of Chemistry, Columbia University, New York, NY 10027, USA, [b]Department of Chemistry and Chemical Biology, Cornell University, Ithaca, NY, 14853, USA

e-mail: Tristan.Lambert@cornell.edu

Table of Contents:

1. General Information 2
2. Substrate Synthesis 2
3. Catalyst Preparation 5
4. ^{29}Si-Shift Determination 10
5. Catalytic Reactions 11
6. References 22
7. Spectra 23

1. General Information

All reactions were performed in a glovebox or using standard air-free techniques. Organic solutions were concentrated using a Buchi rotary evaporator. Methylene chloride, diethyl ether, benzene and toluene were dried using a J.C. Meyer solvent purification system and stored over activated 3Å molecular sieves. Deuterated solvents were stored over activated 3Å molecular sieves. All other solvents were purchased as anhydrous and used as received. All other commercial reagents were used as provided. Flash column chromatography was performed employing 40-63 μm silica gel (SiliaFlash® P60 from Silicycle). Thin-layer chromatography (TLC) was performed on 250 μm glass-backed silica plates (SiliaPlate™ G TLC from Silicycle). ^{1}H and ^{13}C NMR spectra were recorded on Bruker DRX-300, DRX-400 or DRX-500 spectrometers as noted. Data for ^{1}H NMR are reported as follows: chemical shift (δ ppm), multiplicity (s = singlet, br s = broad singlet, d = doublet, t = triplet, q =quartet, p = pentet, dd = doublet of doublets, td = triplet of doublets, m = multiplet), coupling constant (Hz), integration, and assignment. All ^{1}H-NMR experiments were measured relative to the signals of tetramethylsilane (TMS 0.00 ppm) or residual solvent (chloroform 7.26 ppm, methanol 3.31 ppm, acetonitrile 1.94 ppm). Data for ^{13}C are reported in terms of chemical shift relative to the deuterated solvent (chloroform: 77.16 ppm, methanol: 49.00 ppm, acetonitrile: 118.26 ppm). High-resolution mass spectra were obtained from the Columbia University Mass Spectrometry Facility on a Waters XEVO G2XSQToF mass spectrometer equipped with a UPC2 SFC inlet, electrospray ionization (ESI) probe, atmospheric pressure chemical ionization (APCI) probe, and atmospheric solids analysis probe (ASAP). IR spectra were collected on a Perkin Elmer Spectrum Two FT-IR spectrometer.

2. Substrate Synthesis

Bromide synthesis general procedure: To a solution of the benzylic alcohol (1 eq.) in dry benzene (5 mL per mmol of alcohol substrate) was added acetyl bromide (1.5 eq.). The reaction was allowed to stand overnight. The reaction was quenched by slow addition into a beaker of with 15 mL of cold (0 ºC) saturated $NaHCO_3$. The mixture was extracted with 2 portions of ether (2 mL per mL of benzene used) and the ethereal extracts washed with brine. The organic layer was then dried with $MgSO_4$ and then the solvent removed to give the desired bromide. Some bromides were then purified as specified.

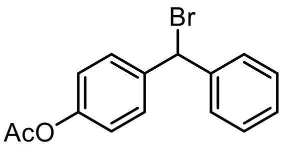

1-phenyl-4'-acetoxybenzyl bromide (S1): Reaction done with 177 mg of alcohol (0.73 mmol). Yield: 193 mg (0.63 mmol, 87% yield)

^{1}H NMR (500 MHz, $CDCl_3$) δ 7.47 (m, 4 H, Ar**H**), 7.38-7.27 (m, 3 H, Ar**H**), 7.07 (m, 2H, Ar**H**), 6.29 (s, 1 H, C**H**BrAr$_2$), 2.30 (s, 3 H, C**H$_3$**CO$_2$). ^{13}C{^{1}H} NMR (126 MHz, $CDCl_3$) δ 169.4, 150.3, 140.8, 138.7, 129.7, 128.7, 128.5, 128.3, 121.7, 54.7, 21.3. IR (thin film, cm^{-1}) 3064, 3028, 1759, 1604, 1503, 1493, 1451, 1368, 1191, 1166, 1017, 911, 862, 748, 696. HRMS (ASAP$^+$) exact mass calc'd for $C_{15}H_{14}O_2Br$ $[M+H]^+$, requires *m/z* 305.0177, found *m/z* 305.0169.

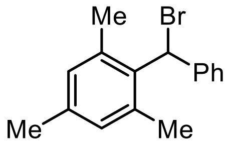

2-(1-bromo-1-phenylmethyl)mesitylene (S2): Reaction done with 453 mg of alcohol (2.00 mmol). Crude product was purified by column chromatography (hexanes). Yield: 428 mg (1.48 mmol, 74% yield)

^{1}H NMR (500 MHz, $CDCl_3$) δ 7.42-7.38 (m, 2H, ArH), 7.31-7.21 (m, 3H, ArH), 6.90-6.85 (overlapping s, 3H, MesH and C**H**Ar$_2$), 2.29 (s, 3H, *p*-MesC**H$_3$**), 2.21 (br s, 6H, *o*-MesC**H$_3$**). ^{13}C{^{1}H} NMR (126 MHz, $CDCl_3$) δ 139.9, 138.3, 134.7, 130.4 (br), 128.5, 128.4, 127.9, 127.2, 52.4, 21.1, 20.9. IR (thin film, cm^{-1}) 3031, 2968, 2917, 2858, 1609, 1492, 1444, 1377, 1163, 1030, 843, 791, 755, 727, 695, 670, 614. HRMS (ASAP$^+$) exact mass calc'd for $C_{16}H_{16}Br$ $[M+H]^+$, requires *m/z* 287.0435, found *m/z* 287.0437.

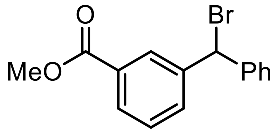

3'-(methoxycarbonyl)benzyl bromide (S3): Reaction done with 121 mg of alcohol (0.50 mmol). Crude product was purified by column chromatography with silica gel (5-15% EtOAc/hexanes) Yield: 112 mg (0.37 mmol, 73% yield)

^{1}H NMR (500 MHz, $CDCl_3$) δ 8.18 (s, 1 H, Ar**H**), 7.99 (d, 1 H, Ar**H**), 7.69 (d, 1 H, Ar**H**), 7.50-7.28 (m, 6H, Ar**H**), 6.33 (s, 1 H, C**H**Br), 3.94 (s, 3 H, C**H**$_3$$CO_2$). $^{13}C\{^{1}H\}$ NMR (126 MHz, $CDCl_3$) δ 166.7, 141.7, 140.7, 133.0, 130.7, 129.6, 129.4, 128.9, 128.8, 128.5, 128.4, 54.5, 52.4. IR (thin film, cm^{-1}) 3031, 2950, 2825, 1720, 1607, 1433, 1286, 1197, 1175, 1106, 1084, 750, 699. HRMS ($ASAP^{+}$) exact mass calc'd for $C_{15}H_{14}O_2Br$ $[M+H]^{+}$, requires *m/z* 305.0177, found *m/z* 305.0175.

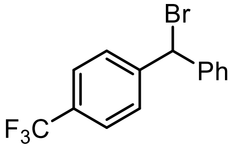

1-phenyl-4-(trifluoromethyl)benzyl bromide (S4): Reaction done with 126 mg of alcohol (0.50 mmol). Yield: 129 mg (0.41 mmol, 82% yield)

^{1}H NMR (400 MHz, $CDCl_3$) δ 7.62 (m, 4 H, CF_3Ar**H**), 7.49-7.31 (m, 5 H, Ar**H**), 6.31 (s, 1 H, C**H**Ar_2). $^{13}C\{^{1}H,^{19}F\}$ NMR NMR (101 MHz, $CDCl_3$) δ 145.1, 140.3, 130.3, 129.0, 128.9, 128.6, 128.5, 125.7, 124.0, 53.9. IR (thin film, cm^{-1}) 2978, 2934, 1780, 1596, 1388, 1360, 1324, 1306, 1278, 1268, 1225, 1202, 1190, 1161, 1106, 1083, 948, 910, 895, 811, 747, 691. HRMS ($ASAP^{+}$) exact mass calc'd for $C_{14}H_9F_3Br$ $[M+H]^{+}$, requires *m/z* 312.9840, found *m/z* 312.9836.

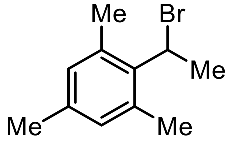

2-(1-bromoethyl)mesitylene (S5): Reaction done with 328 mg of alcohol (2.0 mmol). Crude product was purified by column chromatography with silica gel (hexanes). Yield: 285 mg (1.26 mmol, 63% yield)

^{1}H NMR (500 MHz, $CDCl_3$) δ 6.88 (s, 1 H, Ar**H**), 6.84 (s, 1 H, Ar**H**), 5.75 (q, 1 H, C**H**BrCH_3), 2.61 (s, 3 H, *o*-**C**$\mathbf{H_{3a}}$Ar), 2.38 (s, 3 H, *o*-**C**$\mathbf{H_{3b}}$Ar), 2.27 (s, 3H, *p*-CH3Ar), (d, 3H, CHBrC$\mathbf{H_3}$). $^{13}C\{^{1}H\}$ NMR (126 MHz, $CDCl_3$) δ 138.3, 137.9, 135.5, 135.3, 131.8, 129.3, 46.7, 25.3, 21.4, 20.9, 20.7. IR (thin film, cm^{-1}) 3011, 2968, 2918, 1862, 1610, 1450, 1376, 1216, 1189, 1075, 1044, 1030, 975, 850, 721, 616. HRMS ($ASAP^{+}$) exact mass calc'd for $C_{11}H_{14}Br$ $[M+H]^{+}$, requires *m/z* 225.0279, found *m/z* 225.0275

3. Catalyst Preparation:

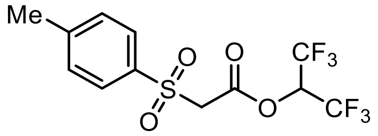

Hexafluoroisopropyl 2-tosylacetate (S6): 2.10 mL of HFIP (20 mmol) was added to 15 mL of dry DCM, along with 1.74 mL (20 mmol) of bromoacetyl bromide. The solution was cooled to 0 °C on an ice bath. Then 2.00 mL of pyridine (25 mmol) in 10 mL of dry DCM was added and the reaction stirred for 1 hr at 0 °C. The reaction was then allowed to warm to r.t. and stirred for an additional 2 hr. The slightly yellow reaction mixture was quenched with 1 M HCl and the aqueous mixture extracted three times with 20 mL of DCM. The organic extracts were washed with sat. $NaHCO_3$, dried with Na_2SO_4 and the solvent removed carefully at ~120 torr. The crude product (~12 mmol from NMR analysis) is the desired bromoacetate with small amounts of HFIP and DCM and was used without further purification.

The bromoacetate was dissolved in 30 mL of DMSO and sodium p-toluenesulfinate (5.34 g, 30 mmol) was added. The reaction was stirred at r.t. overnight. The mixture was diluted with 120 mL of water and extracted 4 times with 30 mL of Et_2O. The combined ethereal extracts were washed 3 times with water and once with brine. The organic layer was then dried with Na_2SO_4 and then the solvent removed via rotovap. The crude product was dissolved in 10 mL of DCM passed through a short silica plug, eluting with ~100 mL of DCM and collecting ~50 mL of product-containing solution. The solvent was removed via rotovap and the solid dried on hi-vac, yielding 2.69 g (7.38 mmol, 37% yield over two steps) of desired product.

^{1}H NMR (500 MHz, $CDCl_3$) δ 7.82 (d, 2 H, Ar**H**), 7.39 (d, 2 H, Ar**H**), 5.68 (m, 1 H, C**H(**$CF_3)_2$), 4.28 (s, 2 H, C**H₂**Ts), 2.47 (s, 3 H, ArC**H₃**). ^{13}C{^{1}H} NMR (126 MHz, $CDCl_3$) δ 160.1, 146.3, 135.2, 130.3, 128.7, 120.0 (J_{CF} = 286 Hz), 67.5 (J_{CF} = 35 Hz), 60.1, 21.9. IR (thin film, cm^{-1}) 2978, 2934, 1780, 1596, 1388, 1360, 1324, 1306, 1278, 1268, 1225, 1202, 1190, 1161, 1106, 1083, 948, 910, 895, 811, 747, 691. HRMS ($ASAP^+$) exact mass calc'd for $C_{12}H_{11}O_4F_6S$ $[M+H]^+$, requires *m/z* 365.0282, found *m/z* 365.0271

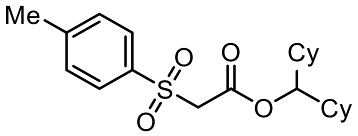

Dicyclohexylmethyl 2-tosylacetate (S7): To a flame-dried 100mL round bottom flask under an argon atmosphere was added 40 mL of dry DCM, 392 mg of dicyclohexylmethanol (2.0 mmol), and 350 µL of Hünig's base (2.0 mmol). The solution was cooled to 0 ºC in an ice bath and 175 µL of bromoacetyl bromide (2.0 mmol) was added slowly dropwise over 30 minutes. The reaction was stirred at 0 ºC for 1 hr, then at r.t. for 1 hr. The reaction was then cooled to 0 ºC and quenched with sat. NH_4Cl. The layers were separated and the aqueous layer was extracted 3 times with 10 mL of DCM. The combined organic layers were washed successively with 1M HCl and sat. $NaHCO_3$, dried with Na_2SO_4 and concentrated to give the crude bromoacetate which was used without purification.

The bromoacetate was dissolved 20 mL of DMF and 713 mg of sodium p-toluenesulfinate (4.0 mmol) added. The reaction was stirred for 4 h. The mixture was diluted with 50 mL of water and extracted 4 times with 20 mL of Et_2O. The combined ethereal extracts were washed 3 times with water and once with sat. NaCl. The organic layer was then dried with $MgSO_4$ and then the solvent removed via rotovap to give 760mg (1.94 mmol, 97% yield over 2 steps) of the desired product.

^{1}H NMR (500 MHz, $CDCl_3$) δ 7.83 (d, 2 H, Ar**H**), 7.36 (d, 2 H, Ar**H**), 4.61 (t, 1 H, C**H**Cy_2), 4.13 (s, 2 H, C**H$_2$**Ts), 2.45 (s, 3 H, ArC**H$_3$**), 1.78-1.46 (m, 12 H), 1.29-0.82 (m, 10 H). ^{13}C{^{1}H} NMR (126 MHz, $CDCl_3$) δ 162.6, 145.4, 136.1, 129.9, 128.8, 84.5, 60.8, 38.3, 29.8, 27.4, 26.4, 26.3, 26.1, 21.9. IR (thin film, cm^{-1}) 2995, 2918, 2849, 1734, 1598, 1449, 1327, 1323, 1290, 1147, 1129, 1088, 1061, 970, 939, 898, 886, 807, 726, 641. HRMS (ESI$^-$) exact mass calc'd for $C_{22}H_{31}O_4S$ [M-H]$^-$, requires *m/z* 391.1943, found *m/z* 391.1952

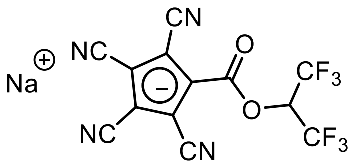

Sodium tetracyano(hexafluoroisopropoxycarbonyl)cyclopentadienide (S8): To a flame-dried, 6-dram vial was added tosylacetate **S6** (364 mg, 1.0 mmol) and 5 mL of dry THF. The solution was cooled to 0 ºC in an ice bath and 60% sodium hydride (120 mg, 3.0 mmol) was added in one portion. The mixture was stirred at 0 ºC for 30 min, then allowed to warm to r.t. and stirred for an additional 30 min. Then a solution of tetracyanodithiin (220 mg, 1.0 mmol) in 5 mL of dry THF was added over approximately 10 minutes. The dark red mixture was then heated at 60 °C for 3 hr. The reaction was then quenched slowly (CAUTION: vigorous gas evolution) with brine (~ 15 mL). The mixture was diluted with 20 mL of EtOAc and 40 mL of brine and the layers separated. The aqueous layer was further extracted two times with 20 mL of EtOAc. The organic layers were washed once with brine, then dried with Na_2SO_4, and the solvent removed via rotovap. The crude product was purified via column chromatography with acidic alumina (10–30% MeCN/EtOAc) to give 96 mg (0.25 mmol, 25% yield) of desired product as a yellow-orange solid.

^{1}H NMR (400 MHz, CD_3CN) δ 6.30 (m, 1 H, C**H**(CF_3)$_2$). ^{13}C{^{1}H,^{19}F} NMR (101 MHz, CD_3CN) δ 158.5, 121.8, 118.5, 114.9, 114.6, 105.1, 102.7, 67.2. IR (thin film, cm^{-1}) 2972, 2929, 2855, 2781, 2227, 1732, 1631, 1563, 1473, 1381, 1292, 1240, 1198, 1105, 1057, 904, 681. HRMS (ESI$^-$) exact mass calc'd for $C_{13}HF_6N_4O_2$ [M-Na]$^-$, requires *m/z* 359.0004, found *m/z* 359.0010.

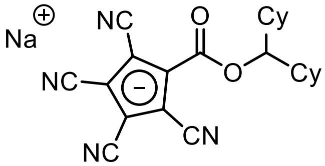

Sodium tetracyano(dicyclohexylmethoxycarbonyl)cyclopentadienide (S9): To a flame-dried, 6-dram vial was added 60% sodium hydride (120 mg, 3.0 mmol) and 3 mL of dry THF and the mixture was cooled to 0 ºC in an ice bath. A solution of tosylacetate **S7** (393 mg, 1.0 mmol) in 2 mL of dry THF was added slowly dropwise. The mixture became very viscous, and 5 mL of dry THF were added to aid stirring. The mixture was stirred at 0 ºC for 40 min. Then a solution of

tetracyanodithiin (216 mg, 1.0 mmol) in 4 mL of dry THF was added over approximately 5 mins, during which the reaction became dark red. The mixture was stirred at 0 ºC for 1 hr and then allowed to warm to r.t. and stirred for an additional 1 hr. The reaction was then quenched slowly (CAUTION: vigorous gas evolution) with brine. The mixture was poured into a separatory funnel, diluted with 20 mL of EtOAc and 20 mL of brine and the layers separated. The aqueous layer was further extracted three times with 20 mL of EtOAc. The organic layers were washed twice with brine, then dried with Na_2SO_4, and the solvent removed via rotovap. The crude product was purified via column chromatography with silica gel (0–1% MeCN/EtOAc) to give 278 mg (0.68 mmol, 68% yield) of desired product.

^{1}H NMR (500 MHz, CD_3CN) δ 4.82 (t, 1 H, C**H**Cy_2), 1.77-1.56 (m, 12H, alkyl CH), 1.34-0.98 (m, 10H, alkyl H). ^{13}C{^{1}H} NMR (126 MHz, CD_3CN) δ 162.3, 124.1, 116.0, 115.2, 103.6, 101.0, 82.8, 39.2, 30.5, 28.4, 27.1, 26.9, 26.7. IR (thin film, cm^{-1}) 2928, 2851, 2221, 1689, 1471, 1448, 1274, 1131, 1096, 933, 894, 783. HRMS (ESI$^-$) exact mass calc'd for $C_{23}H_{23}O_2N_4$ [M-Na]$^-$, requires *m/z* 387.1821, found *m/z* 387.1832

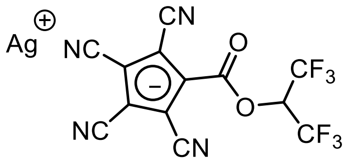

Silver tetracyano(hexafluoroisopropoxycarbonyl)cyclopentadienide (S10): Na salt **S8** (38 mg, 0.10 mmol) was dissolved in 1.0 mL of acetone in a 2-dram vial wrapped in tin foil. To this, a solution of $AgNO_3$ (68 mg, 0.40 mmol) in 0.50 mL of water was added and the reaction stirred overnight. The solution was then filtered and the tan solid washed with water. The solid was collected and dried on hi-vac to give 36 mg (0.077 mmol, 77% yield) of the desired product.

^{1}H NMR (400 MHz, CD_3CN) δ 6.30 (m, 1 H, C**H**$(CF_3)_2$). ^{13}C{^{1}H,^{19}F} NMR (101 MHz, CD_3CN) δ 158.5, 121.7, 118.6, 114.9, 114.5, 105.1, 102.6, 67.2. IR (thin film, cm^{-1}) 2933, 2853, 2236, 2222, 1739, 1476, 1362, 1241, 1193, 1102, 1059, 924, 892, 766, 716, 686. HRMS (ASAP$^+$) exact mass calc'd for $C_{13}HF_6N_4O_2$, requires *m/z* 359.0004, found *m/z* 359.0007

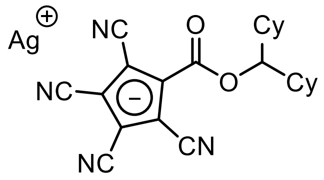

Silver tetracyano(dicyclohexylmethoxycarbonyl)cyclopentadienide (S11): Na salt **S9** (113 mg, 0.28 mmol) was dissolved in 2.5 mL of acetone in a 2-dram vial wrapped in tin foil. To this, a solution of $AgNO_3$ (148 mg, .87 mmol) in 0.50 mL of water was added and the reaction stirred for 3 days. The very fine precipitate was collected over celite. The brown solid was then dissolved in MeCN and the solution collected. The MeCN was removed via rotovap and the material dried on hi vac to give 98 mg (0.20 mmol, 72% yield) of the desired product.

^1H NMR (500 MHz, CD_3CN) δ 4.82 (t, 1 H, C**H**Cy_2), 1.77-1.57 (m, 12H, alkyl CH), 1.34-0.98 (m, 10H, alkyl H). $^{13}C\{^1H\}$ NMR (126 MHz, CD_3CN) δ 162.2, 124.2, 116.0, 115.2, 103.6, 101.0, 82.8, 39.2, 30.5, 28.4, 27.1, 26.9, 26.7. IR (thin film, cm^{-1}). 2925, 2851, 2224, 1708, 1474, 1447, 1254, 1117, 1094, 1065, 961, 934, 893. HRMS (ESI^-) exact mass calc'd for $C_{23}H_{23}O_2N_4$ $[M-Ag]^-$, requires *m/z* 387.1821, found *m/z* 387.1825.

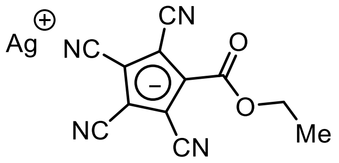

Silver tetracyano(ethoxycarbonyl)cyclopentadienide (S12): Sodium tetracyano-(ethoxycarbonyl)cyclopentadienide[1] (92 mg, 0.32 mmol) was dissolved in 2.0 mL of acetone in a 6-dram vial wrapped in tin foil. To this, a solution of $AgNO_3$ (82 mg, 0.482 mmol) in 0.50 mL of water was added and the reaction stirred overnight. The solution was then filtered and the tan solid washed with water and then washed with acetone. The solid was collected and dried on hi-vac to give 110 mg (0.32 mmol, 99% yield) of the desired product.

^1H NMR (500 MHz, CD_3CN) δ 4.30 (q, 2 H, C**H_2**CH_3), 1.33 (t, 3H, CH_2C**H_3**). $^{13}C\{^1H\}$ NMR (126 MHz, CD_3CN) δ 161.9, 124.1, 115.9, 115.1, 103.5, 100.9, 61.5, 14.4. IR (thin film, cm^{-1}) 2982, 2772, 2239, 2217, 1712, 1702, 1482, 1468, 1445, 1386, 1261, 1135, 1107, 1012, 780, 653. HRMS (ESI^-) exact mass calc'd for $C_{12}H_5O_2N_4$ $[M-Ag]^-$, requires *m/z* 237.0413, found *m/z* 237.0412.

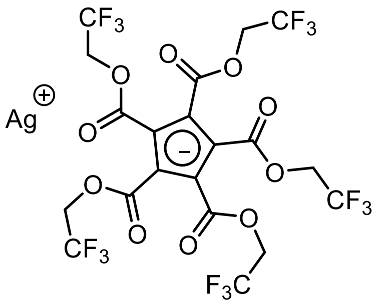

Silver penta(trifluoroethoxycarbonyl)cyclopentadienide (S13): NMe_4 penta(trifluoroethoxy-carbonyl)cyclopentadienide[2] (140 mg, 0.18 mmol) was added to a solution of AgOTf (70.0 mg, 0.27 mmol) in 2 mL of diethyl ether in a 2-dram vial wrapped in tin foil. The reaction was stirred overnight. The mixture was then cooled to –14 ºC in a freezer and filtered. The solution was then washed with water, dried with Na_2SO_4 and evaporated to give 125 mg (0.16 mmol, 86% yield) of the desired product as an off-white solid.

^{1}H NMR (400 MHz, CD_3CN) δ 4.59 (q, 10 H, C**H**$_2$CF$_3$). ^{13}C{^{1}H} NMR (101 MHz, CD_3CN) δ 164.69, 124.71, 117.03, 60.92. IR (thin film, cm^{-1}). 2975, 1695, 1661, 1446, 1413, 1277, 1256, 1149, 1047, 958, 650, 628. HRMS (ESI$^-$) exact mass calc'd for $C_{20}H_{10}O_{10}F_{15}$ [M-Ag]$^-$, requires *m/z* 695.0034, found *m/z* 695.0042

4. ^{29}Si-shift Determination

Triisopropylsilyl synthesis general procedure: The silver salt (0.01 mmol) was added to a 1-dram vial with 200 µL of dry acetonitrile and trityl chloride (2.78mg, 0.01 mmol). The mixture immediately turned yellow-orange and a white precipitate was generated. The mixture was stirred for 5 minutes and then filtered. The acetonitrile was removed in vacuo. The material was then washed with 450 µL of pentane/benzene (2:1) and then dried on high vac. The trityl salt was used immediately thereafter.

The trityl salt was dissolved in 600 µL of d_4-1,2-dichlorobenzene. 1 eq. of triisopropylsilane (2.0 µL, 0.01 mmol) was added and the mixture became colorless over the course of several minutes (5-15 minutes). ^{1}H,^{29}Si-HMBC spectra were collected with a delay optimized for 6 Hz coupling.

Triisopropylsilyl penta(methoxycarbonyl)cyclopentadienide (5a): ^{29}Si δ 35 ppm

Triisopropylsilyl penta(trifluoroethoxycarbonyl)cyclopentadienide (5b): ^{29}Si δ 42 ppm

Triisopropylsilyl tetracyano(ethoxycarbonyl)cyclopentadienide (6a): ^{29}Si δ 30 ppm

Triisopropylsilyl tetracyano(dicyclohexylmethoxycarbonyl)cyclopentadienide (6b): ^{29}Si δ 30 ppm

Triisopropylsilyl tetracyano(hexafluoroisopropoxycarbonyl)cyclopentadienide (6c): ^{29}Si δ 32 ppm

5. Catalytic Reactions

Tert-butyldimethylsilyl (Ethoxycarbonyl)tetracyanocyclopentadienide: Silver salt **S12** (5.1 mg, 0.015 mmol) was dissolved in 200 µL of dry acetonitrile in a flame-dried 1-dram vial. Trityl chloride (4.2 mg, 0.015 mmol) was added and the mixture turned bright yellow-orange with a white precipitate. The mixture was stirred for 5 minutes and then filtered. The vial was washed with 200 µL of acetonitrile and then the solvent removed in vacuo. The crude trityl salt was then dissolved in 1000 µL of dry DCE. To this, tert-butyldimethylsilane (2.5 µL, 0.015 mmol) was added and the solution quickly became colorless. This solution was then used immediately for reactions.

Allylation general procedure: The bromide (0.1 mmol) and allyltrimethylsilane (0.2 mmol) were added to a flame-dried 2-dram vial with 800 µL of dry DCE under an atmosphere of argon. A solution of the catalyst (200 µL, 0.003 mmol) was added and the reaction heated at the specified temperature. After 3 hours, the reaction was quenched with triethylamine and purified.

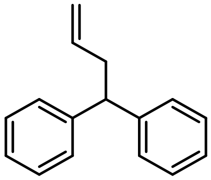

4,4-diphenyl-1-butene (12): Reaction was conducted at 50 °C. Upon completion of the reaction, the reaction was quenched with the addition of triethylamine and concentrated. The crude material was purified via column chromatography (0 → 2% CH_2Cl_2/hexanes). The product was collected as a colorless oil (19 mg, 89% yield). Spectra of the product were consistent with previous reports[3]. ^{1}H-NMR (500 MHz, $CDCl_3$) δ 7.31-7.09 (m, 10 H, Ar**H**), 5.72 (m, 1 H,

C**H**=CH_2), 5.03 (m, 1 H, CH=C**H$_a$**H$_b$), 4.94 (m, 1 H, CH=CH$_a$**H$_b$**), 4.01 (t, 1 H, Ph_2C**H**), 2.82 (m, 1 H, C**H$_2$**CH=CH_2)

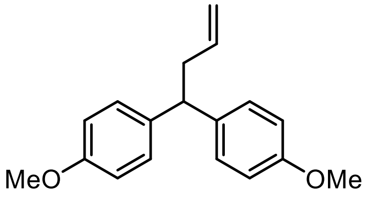

4,4-bis(4-methoxyphenyl)-1-butene (13): Reaction was conducted at 50 °C. Upon completion of the reaction, the reaction was quenched with the addition of triethylamine and concentrated. The crude material was purified via column chromatography (10 → 20% CH_2Cl_2/hexanes). The product was collected as a colorless oil (26 mg, 98% yield). Spectra of the product were consistent with previous reports[3]. ^{1}H NMR (500 MHz, $CDCl_3$) δ 7.19-7.14 (m, 4 H, ArH), 6.87-6.83 (m, 4 H, Ar**H**), 5.75 (m, 1 H, C**H**=CH_2), 5.05 (m, 1 H, CH=C**H$_a$**H$_b$), 4.97 (m, 1 H, CH=CH$_a$**H$_b$**), 3.95 (t, 1 H, Ar_2C**H**), 3.80 (s, 6 H, C**H$_3$**O), 2.83-2.75 (m, 2 H, C**H$_2$**CH=CH_2)

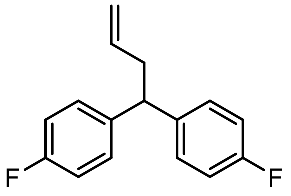

4,4-bis(4-fluorophenyl)-1-butene (14): Reaction was conducted at 50 °C. Upon completion of the reaction, the reaction was quenched with the addition of triethylamine and concentrated. The crude material was purified via column chromatography (hexanes). The product was collected as a colorless oil (17 mg, 70% yield). Spectra of the product were consistent with previous reports[4]. ^{1}H NMR (500 MHz, $CDCl_3$) δ 7.19-7.08 (m, 4 H, ArH), 7.01-6.89 (m, 4 H, ArH), 5.69 (m, 1 H, CH=C**H$_2$**), 5.06-4.89 (m, 2 H, CH=C**H$_2$**), 3.99 (t, 1 H, Ar_2C**H**), 2.79-2.72 (m, 2 H, C**H$_2$**CH=CH_2)

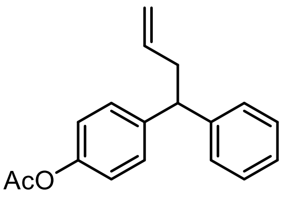

1-acetyl-4-(1-phenylbut-3-enyl)benzene (15): Reaction was conducted at 50 °C. Upon completion of the reaction, the reaction was quenched with the addition of triethylamine and concentrated. The crude material was purified via column chromatography (0 → 5% EtOAc/hexanes). The product was collected as a colorless oil (26 mg, 98% yield). ^{1}H NMR (400 MHz, $CDCl_3$) δ 7.34-7.18 (m, 7H, Ar**H**), 7.02 (m, 2 H, ArH), 5.73 (m, 1 H, C**H**=CH_2), 5.06 (m, 1 H, CH=C**H_a**H_b), 4.98 (m, 1 H, CH=CH_a**H_b**), 4.04 (t, 1 H, ArArC**H**CH_2), 2.83 (td, 2 H, ArArCHC**H_2**), 2.30 (s, 3H, C**H_3**CO_2). $^{13}C\{^1H\}$ NMR (101 MHz, $CDCl_3$) δ 169.5, 148.9, 144.1, 142.1, 136.6, 128.8, 128.5, 128.0, 126.3, 121.4, 116.5, 50.7, 40.0, 21.2. IR (thin film, cm^{-1}) 3064, 3029, 2921, 1760, 1752, 1506, 1369, 1018, 909, 731, 699. HRMS ($ASAP^+$) exact mass calc'd for $C_{18}H_{19}O_2$ $[M+H]^+$, requires *m/z* 267.1385, found *m/z* 267.1395

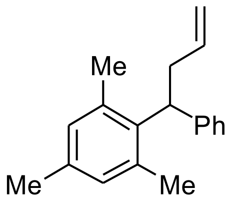

2-(1-phenylbut-3-enyl)mesitylene (16): Reaction was conducted at 50 °C. Upon completion of the reaction, the reaction was quenched with the addition of triethylamine and concentrated. The crude material was purified via column chromatography (hexanes). The product was collected as a colorless oil (24 mg, 96% yield). ^{1}H NMR (400 MHz, $CDCl_3$) δ 7.38-7.16 (m, 5 H, ArH), 6.87 (s, 2 H, ArH), 5.81 (m, 1 H, C**H**=CH2), 5.81 (m, 1 H, C**H**=CH_2), 5.16 (m, 1 H, CH=C**H_a**H_b), 5.01 (m, 1 H, CH=CH_a**H_b**), 4.67 (t, 1 H, ArArC**H**CH_2), 3.15 (m, 1 H, ArArCHC**H_a**H_b), 2.85 (m, 1 H, ArArCHC**H_a**H_b) 2.32 (s, 3 H, *p*-ArC**H_3**), 2.20 (br s, 6 H, *o*-ArC**H_3**). $^{13}C\{^1H\}$ NMR (101 MHz, $CDCl_3$) δ 144.1, 138.1, 137.3, 136.9, 135.5, 129.9, 128.1, 127.2, 125.5, 115.7, 43.4, 35.8, 21.4, 20.8. IR (thin film, cm^{-1}) 3064, 3013, 2978, 2920, 2862, 1639, 1611, 1601, 1495, 1447, 1031, 995, 909, 848, 765, 731, 696, 650, 629. HRMS ($ASAP^+$) exact mass calc'd for $C_{19}H_{23}$ $[M+H]^+$, requires *m/z* 251.1800, found *m/z* 251.1786

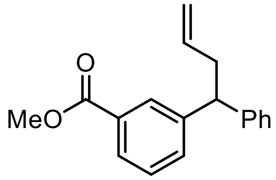

methyl 3-(1-phenylbut-3-enyl)benzoate (17): Reaction was conducted at 80 °C. Upon completion of the reaction, the reaction was quenched with the addition of triethylamine and concentrated. The crude material was purified via column chromatography (0 → 10% CH_2Cl_2/hexanes). The product was collected as a colorless oil (19 mg, 71% yield). ^{1}H NMR (400 MHz, $CDCl_3$) δ 7.96 (s, 1 H, Ar**H**), 7.87 (m, 1 H, Ar**H**), 7.46-7.15 (m, 7H, Ar**H**) 5.71 (m, 1 H, C**H**=CH_2), 5.04 (m, 1 H, CH=C$\mathbf{H_a}$$H_b$), 4.96 (m, 1 H, CH=C$H_a$$\mathbf{H_b}$), 4.08 (t, 1H, C**H**$Ar_2$), 3.91 (s, 3H, C$\mathbf{H_3}$$CO_2$), 2.85 (t, 1 H, Ar_2CHC$\mathbf{H_2}$). ^{13}C{^{1}H} NMR (101 MHz, $CDCl_3$) δ 167.3, 145.0, 144.0, 136.5, 132.8, 130.4, 129.2, 128.7, 128.6, 128.0, 127.7, 126.6, 116.8, 52.2, 51.2, 39.9. IR (thin film, cm^{-1}) 3064, 3028, 2951, 2843, 1718, 1643, 1602, 1587, 1496, 1445, 1433, 1279, 1196, 1106, 1084, 993, 915, 748, 702. HRMS (ASAP$^+$) exact mass calc'd for $C_{18}H_{19}O_2$ $[M+H]^+$, requires *m/z* 267.1385, found *m/z* 267.1392

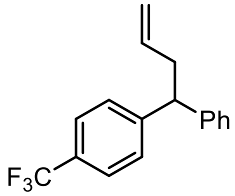

4-phenyl-4-(4-trifluoromethylphenyl)-1-butene (18): Reaction was conducted at 80 °C. Upon completion of the reaction, the reaction was quenched with the addition of triethylamine and concentrated. The crude material was purified via column chromatography (0 → 2% CH_2Cl_2/hexanes). The product was collected as a colorless oil (9.2 mg, 33% yield). Spectra of the product were consistent with previous reports[3]. ^{1}H NMR (400 MHz, $CDCl_3$) δ 7.52 (d, 2 H, CF3-Ar**H**), 7.38-7.17 (m, 7 H, Ar**H**), 5.70 (m, 1 H, C**H**=CH_2), 5.09-4.93 (m, 1 H, CH=C$\mathbf{H_2}$), 4.07 (t, 1H, C**H**Ar_2), 2.91-2.75 (m, 2 H, C$\mathbf{H_2}$CH=CH_2)

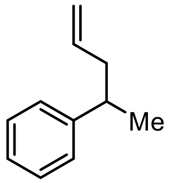

4-phenyl-1-pentene (10): Reaction was conducted at 80 °C. Upon completion of the reaction, the reaction was quenched with the addition of triethylamine and concentrated. The crude

material was purified via column chromatography (pentane). The product was collected as a colorless oil (9.4 mg, 64% yield). Spectra of the product were consistent with previous reports[5]. ^{1}H-NMR (500 MHz, $CDCl_3$) δ 7.33-7.14 (m, 10 H, Ar**H**), 5.72 (m, 1 H, C**H**=CH_2), 5.03-4.92 (m, 2 H, CH=C**H_2**), 2.79 (m, Ar_2C**H**), 2.44-2.35 (m, 1 H, C**H_a**H_bCH=CH_2), 2.33-2.24 (m, 1 H, CH_a**H_b**CH=CH_2), 1.26 (d, 3 H, CHC**H_3**)

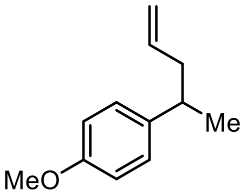

4-(4-methoxyphenyl)pent-1-ene (19): Reaction was conducted at 80 °C. Upon completion of the reaction, the reaction was quenched with the addition of triethylamine and concentrated. The crude material was purified via column chromatography (0 → 20% CH_2Cl_2/hexanes). The product was collected as a colorless oil (15 mg, 85% yield). Spectra of the product were consistent with previous reports[5]. ^{1}H NMR (400 MHz, $CDCl_3$) δ 7.14-7.09 (m, 2 H, Ar**H**), 6.87-6.82 (m, 2 H, Ar**H**), 5.75-5.66 (m, 1 H, C**H**=CH_2), 5.02-4.92 (m, 2 H, CH=C**H_2**), 3.79 (s, 3 H, C**H_3**O), 2.75 (m, 1 H, ArC**H**(allyl)CH_3), 2.39-2.31 (m, 1 H, C**H_a**CH_bCH=CH_2), 2.30-2.22 (m, 1 H, CH_aC**H_b**CH=CH_2), 1.23 (s, 3 H, ArCH(allyl)C**H_3**)

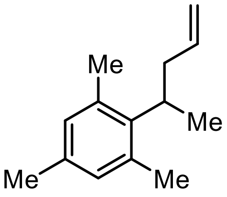

2-(1-methylbut-3-enyl)mesitylene (20): Reaction was conducted at 50 °C. Upon completion of the reaction, the reaction was quenched with the addition of triethylamine and concentrated. The crude material was purified via column chromatography (hexanes). The product was collected as a colorless oil (12 mg, 65% yield). Spectra of the product were consistent with previous reports[6]. ^{1}H NMR (500 MHz, $CDCl_3$) δ 6.82 (s, 2 H, Ar**H**), 5.80-5.69 (m, 1 H, C**H**=CH_2), 5.04 (m, 1 H, CH=C**H_a**H_b), 4.95 (m, 1 H, CH=CH_a**H_b**), 3.29 (m, 1 H, ArC**H**(allyl)CH_3), 2.47 (m, 2 H, C**H_2**CH=CH overlapped), 2.36 (s br, 6 H, *o*-ArC**H_3**), 2.25 (s, 3 H, *p*-ArC**H_3**), 1.31 (d, 3 H, ArCH(allyl)C**H_3**)

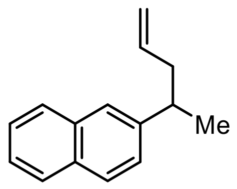

2-(1-methylbut-3-enyl)naphthalene (21): Reaction was conducted at 80 °C. Upon completion of the reaction, the reaction was quenched with the addition of triethylamine and concentrated. The crude material was purified via column chromatography (0 → 2% CH_2Cl_2/hexanes). The product was collected as a colorless oil (16 mg, 82% yield). Spectra of the product were consistent with previous reports[5]. ^{1}H NMR (500 MHz, $CDCl_3$) δ 7.92-7.35 (m, 7 H, ArH), 5.77 (m, 1 H, CH=C**H₂**), 5.11-4.94 (m, 2 H, CH=C**H₂**), 2.99 (m, 1 H, ArC**H**CH_3), 2.57-2.36 (m, 2 H, C**H₂**CH=CH_2), 1.36 (d, 1 H, ArC**H**C**H₃**)

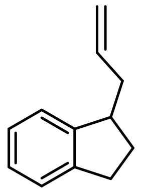

1-allylindane (22): Reaction was conducted at 80 °C. Upon completion of the reaction, the reaction was quenched with the addition of triethylamine and concentrated. The crude material was purified via column chromatography (pentane). The product was collected as a colorless oil (17 mg, 89% yield). Spectra of the product were consistent with previous reports[7]. ^{1}H NMR (500 MHz, $CDCl_3$) δ 7.27-7.12 (m, 4 H, Ar**H**), 5.87 (m, 1 H, C**H**=CH_2), 5.14-5.02 (m, 2 H, CH=C**H₂**), 3.22 (m, 1 H, ArC**H**(allyl)), 2.98-2.80 (m, 2 H, ArC**H₂**), 2.58 (m, 1 H, alkylH), 2.30-2.20 (m, 2 H, alkyl H), 1.80-1.70 (m, 1 H, alkyl H)

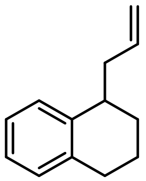

1,2,3,4-tetrahydro-1-allylnaphthalene (23): Reaction was conducted at 80 °C. Upon completion of the reaction, the reaction was quenched with the addition of triethylamine and concentrated. The crude material was purified via column chromatography (pentane). The product was collected as a colorless oil (18 mg, 88% yield). Spectra of the product were

consistent with previous reports[5]. ^{1}H NMR (500 MHz, $CDCl_3$) δ 7.25-7.06 (m, 4 H, ArH), 5.86 (m, 1 H, C**H**=CH_2), 5.12-5.02 (m, 2 H, CH=C**H_2**), 2.93-2.71 (m, 3 H, alkyl H), 2.55-2.47 (m, 1 H, X), 2.39-2.30 (m, 1 H, X), 1.90-1.68 (m, 4 H, alkyl H)

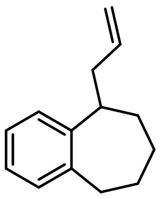

1-allylbenzosuberan (24): Reaction was conducted at 80 °C. Upon completion of the reaction, the reaction was quenched with the addition of triethylamine and concentrated. The crude material was purified via column chromatography (pentane). The product was collected as a colorless oil (4:1 product:elimination) (16 mg, 68% yield). ^{1}H NMR (500 MHz, $CDCl_3$) δ 7.23-7.10 (m, 4 H, ArH), 5.87 (m, 1 H, C**H**=CH_2), 5.11 (m, 1 H, CH=C**H_a**H_b), 5.06 (m, 1 H, CH=CH_a**H_b**), 2.89 (m, 1 H, ArC**H**(allyl)CH_2), 2.92-2.85 (m, 2 H, ArC**H_2**CH_2), 2.63 (m, 1 H, CH_2=CHC**H_a**CH_b), 2.46 (m, 1 H, CH_2=CHCH_aC**H_b**), 2.46-1.42 (m, 6 H, alkyl H) $^{13}C\{^1H\}$ NMR (126 MHz, $CDCl_3$) 145.4, 142.8, 138.0, 129.7 (2 peaks overlapping), 126.1, 126.0, 116.1, 37.8, 36.3, 36.2, 32.7, 32.6, 28. IR (thin film, cm^{-1}) 3074, 3017, 2920, 2851, 1640, 1490, 1448, 994, 909, 781, 755, 745. HRMS ($ASAP^+$) exact mass calc'd for $C_{14}H_{19}$ $[M+H]^+$, requires *m/z* 187.1487, found *m/z* 187.1490

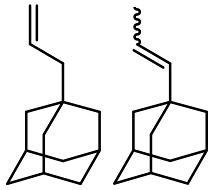

1-allyladamantane (25a) and 1-(1-propenyl)adamantane (25b): Reaction was conducted at 80 °C. Upon completion of the reaction, the reaction was quenched with the addition of triethylamine and concentrated. The crude material was purified via column chromatography (hexanes). The products were collected as a colorless oil as a 2:1 mixture (**25a**:**25b**). (5.3 mg, 30% combined yield). Spectra of the products were consistent with previous reports[5,8]. ^{1}H NMR (500 MHz, $CDCl_3$) δ 5.82 (m, 1 H, C**H**=CH_2), 5.05-4.95 (m, 2 H, CH=C**H_2**), 1.98-1.42 (m, 17 H,

alkylH and C**H**$_2$CH=CH$_2$) **25b:** 5.31-5.19 (m, 2 H, C**H**=C**H**CH$_3$), 1.98-1.42 (m, 18 H, alkyl**H** and CH=CHC**H**$_3$),

Aryl nucleophile scope general procedure: Diphenylmethyl bromide (0.1 mmol), allyltrimethylsilane (0.2 mmol) and the corresponding arene (0.2 mmol) was added to a flame-dried 2-dram vial with 800 μL of dry DCE under an atmosphere of argon. A solution of the catalyst (200 μL, 0.003 mmol) was added and the reaction heated at 50 °C. After 3 hours, the reaction was quenched with triethylamine and purified.

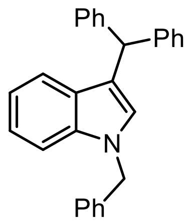

N-benzyl-3-benzhydrylindole (34): Upon completion of the reaction, the reaction was quenched with the addition of triethylamine and concentrated. The crude material was purified via column chromatography (10 → 20% CH_2Cl_2/hexanes). The product was collected as a colorless oil (34 mg, 92% yield). ^{1}H NMR (500 MHz, $CDCl_3$) δ 7.36-7.21 (m, 15 H, ArH), 7.15 (t, 1 H, ArH), 7.09 (m, 2 H, ArH), 7.00 (m, 1 H, ArH), 6.57 (s, 1 H, indole C2-H), 5.27 (s, 2 H, N-C**H**$_2$Ph). ^{13}C{^{1}H} NMR (126 MHz, $CDCl_3$) δ 144.1, 137.9, 137.2, 129.1, 128.8, 128.5, 128.4, 127.8, 127.6, 126.5, 126.3, 122.0, 120.3, 119.2, 119.1, 109.9, 50.1, 49.0. IR (thin film, cm^{-1}) 3059, 3027, 1711, 1658, 1614, 1600, 1494, 1466, 1454, 1355, 1278, 1176, 1077, 1030, 740, 698. HRMS (ASAP$^+$) exact mass calc'd for $C_{28}H_{24}N$ $[M+H]^+$, requires *m/z* 374.1090, found *m/z* 374.1904

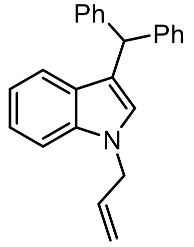

N-allyl-3-benzhydrylindole (35): Upon completion of the reaction, the reaction was quenched with the addition of triethylamine and concentrated. The crude material was purified via column

chromatography (10 → 20% CH_2Cl_2/hexanes). The product was collected as a colorless oil (28 mg, 87% yield). ^{1}H NMR (500 MHz, $CDCl_3$) δ 7.38-7.19 (m, 13 H, ArH), 7.02 (t, 1 H, ArH), 6.52 (s, 1 H, indole C_2-H), 5.99 (m, 1 H, C**H**=CH_2), 5.73 (s, 1 H, Ar_3CH), 5.20 (m, 1 H, CH=C$\mathbf{H_a}$$H_b$), 5.10 (m, 1 H, CH=CH$_a$$\mathbf{H_b}$), 4.68 (m, 2 H, N-C$\mathbf{H_2}$CHCH$_2$). ^{13}C{^{1}H} NMR (126 MHz, $CDCl_3$) δ 144.2, 137.0, 133.8, 129.1, 128.4, 127.9, 127.8, 126.3, 121.8, 120.2, 119.1, 118.8, 117.1, 109.70, 49.9, 48.9. IR (thin film, cm^{-1}) 3058, 3025, 2924, 2858, 1600, 1549, 1493, 1466, 1449, 1389, 1333, 1191, 1078, 1031, 1014, 990, 920, 738, 698. HRMS (ASAP$^+$) exact mass calc'd for $C_{24}H_{22}N$ [M+H]$^+$, requires *m/z* 324.1752, found *m/z* 324.1747.

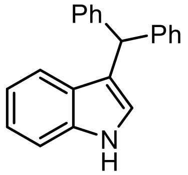

3-benzhydrylindole (36): Upon completion of the reaction, the reaction was quenched with the addition of triethylamine and concentrated. The crude material was purified via column chromatography (10 → 30% CH_2Cl_2/hexanes). The product was collected as a colorless oil (14 mg, 48% yield). Spectra of the product were consistent with previous reports[9]. NMR (500 MHz, $CDCl_3$) δ 7.90 (s br, 1 H, indole N**H**), 7.36-7.11 (m, 13 H, Ar**H**), 7.02-6.95 (m, 1 H, Ar**H),** 6.55 (s, 1 H, indole C2-**H**), 5.67 (s, 1 H, Ph_2C**H**)

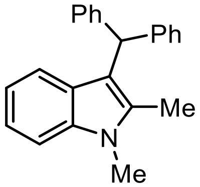

3-benzhydryl-1,2-dimethylindole (37): Upon completion of the reaction, the reaction was quenched with the addition of triethylamine and concentrated. The crude material was purified via column chromatography (0 → 5% CH_2Cl_2/hexanes). The product was collected as a colorless oil (27 mg, 87% yield). ^{1}H NMR (500 MHz, $CDCl_3$) δ 7.36-7.19 (m, 11 H, ArH), 7.13 (t, 1 H, ArH), 7.02 (d, 1 H, ArH), 6.91 (t, 1 H, ArH), 5.80 (s, 1 H, Ar_3C**H**), 3.68 (m, 3 H, N-C$\mathbf{H_3}$), 2.28 (s, 3 H, indole C2-C$\mathbf{H_3}$). ^{13}C{^{1}H} NMR (126 MHz, $CDCl_3$) δ 144.21, 136.83, 134.00, 129.28, 128.27, 127.55, 126.11, 120.42, 119.73, 118.88, 113.61, 108.63, 48.18, 29.65, 10.87. IR (thin

film, cm^{-1}) 3058, 3024, 2925, 2854, 1601, 1494, 1470, 1447, 1367, 1333, 1031, 739, 699. HRMS ($ASAP^+$) exact mass calc'd for $C_{23}H_{22}N$ $[M+H]^+$, requires *m/z* 312.1752, found *m/z* 312.1758.

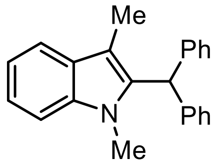

2-benzhydryl-1,3-dimethylindole (38): Upon completion of the reaction, the reaction was quenched with the addition of triethylamine and concentrated. The crude material was purified via column chromatography (0 → 5% CH_2Cl_2/hexanes). The product was collected as a white solid (26 mg, 84% yield). ^1H NMR (500 MHz, $CDCl_3$) δ 7.58-7.53 (m, 1 H, ArH), 7.36-7.10 (m, 13 H, ArH), 5.94 (s, 1 H, ArC**H**Ph_2), 3.52 (s, 1 H, NC**H**$_3$), 1.88 (s, 3 H, indole C3-C**H**$_3$) $^{13}C\{^1H\}$ NMR (126 MHz, $CDCl_3$) δ 141.3, 136.7, 136.6, 129.3, 128.8, 128.5, 126.6, 121.2, 118.7, 118.3, 108.9, 108.6, 48.3, 30.5, 9.1. IR (thin film, cm^{-1}) 3026, 2939, 2867, 1601, 1494, 1473, 1448, 1363, 1327, 1238, 1077, 1029, 1013, 770, 745, 723, 697, 671. HRMS ($ASAP^+$) exact mass calc'd for $C_{23}H_{22}N$ $[M+H]^+$, requires *m/z* 312.1752, found *m/z* 312.1751

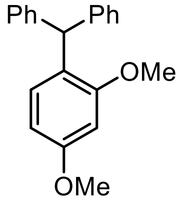

4-benzhydryl-1,3-dimethoxybenzene (39): Upon completion of the reaction, the reaction was quenched with the addition of triethylamine and concentrated. The crude material was purified via column chromatography (10 → 20% CH_2Cl_2/hexanes). The product was collected as a colorless oil and as an 8:1 mixture of the mono- and di-alkylated products (26 mg, 80% yield). Spectra of the product were consistent with previous reports[10]. ^1H NMR (500 MHz, $CDCl_3$) δ 7.32-7.08 (m, 10 H, Ar**H**), 6.77 (d, 1 H, ArH), 6.49 (d, 1 H, Ar**H**), 6.42 (dd, 1 H, Ar**H**), 5.86 (s, 1 H, Ph_2C**H**), 3.81 (s, 3 H, C**H**$_3$O), 3.72 (s, 3 H, C**H**$_3$O)

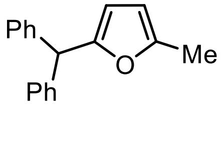

4-benzhydryl-1,3-dimethoxybenzene (40): Upon completion of the reaction, the reaction was quenched with the addition of triethylamine and concentrated. The crude material was purified via column chromatography (0 → 5% EtOAc/hexanes). The product was collected as a colorless oil. (24 mg, 97% yield). Spectra of the product were consistent with previous reports[10]. ^{1}H NMR (500 MHz, $CDCl_3$) 7.31-7.14 (m, 10 H, ArH), 5.88-5.85 (m, 1H, furan**H**), 5.74 (d, 1 H, furan**H**), 5.39 (s, 1 H, Ph_2C**H**), 2.24 (s, 3H, C**H_3**)

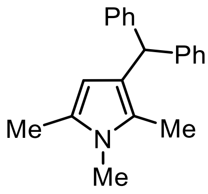

3-benzhydryl-1,2,5-trimethylpyrrole (42): Upon completion of the reaction, the reaction was quenched with the addition of triethylamine and concentrated. The crude material was purified via column chromatography (0 → 5% EtOAc/Hexanes buffered with 1% NEt_3). The product was collected as a colorless oil (15 mg, 55% yield). ^{1}H NMR (500 MHz, $CDCl_3$) δ 7.30-7.18 (m, 10 H, ArH), 5.47 (s, 1 H, C**H**Ph_2), 5.34 (s, 1 H, pyrrole Ar**H**), 3.39 (s, 3 H, NC**H_3**), 2.17 (s, 3 H, 5-pyrrole C**H_3**), 2.07 (s, 3 H, 2-pyrrole C**H**$_3$). $^{13}C\{^1H\}$ NMR (126 MHz, $CDCl_3$) δ 145.6, 129.2, 129.0, 128.0, 127.9, 125.6, 120.4, 48.9, 30.2, 12.5, 10.3. IR (thin film, cm^{-1}) 3023, 2919, 2857, 1599, 1493, 1448, 1395, 1347, 1032, 763, 741, 700 HRMS (ASAP$^+$) exact mass calc'd for $C_{20}H_{22}N$ $[M+H]^+$, requires *m/z* 276.1752, found *m/z* 276.1740.

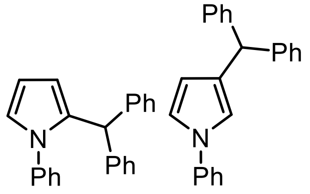

2-benzhydryl-1-phenylpyrrole: Upon completion of the reaction, the reaction was quenched with the addition of triethylamine and concentrated. The crude material was purified via column chromatography (0 → 10% CH_2Cl_2/hexanes). The product was collected as a colorless solid as a mixture (4:1) of the 2- and 3-benzhydrylpyrroles (25 mg, 81% yield). ^{1}H NMR (500 MHz, $CDCl_3$) δ 7.40-7.00 (m, 16 H, ArH + pyrrole C2-H minor), 6.77 (dd, 1 H, pyrrole C5-H major), 6.63 (m, 1 H, pyrrole C-5H minor), 6.22 (t, 1 H, pyrrole C4-H major), 6.13 (dd, 1 H, pyrrole C4-minor), 5.82 (dd, 1 H, pyrrole C-2 major), 5.40 (s, 1 H, Ph_2C**H** minor), 5.30 (s, 1 H, Ph_2C**H**

major) $^{13}C\{^1H\}$ NMR (126 MHz, $CDCl_3$) δ (major) 143.3, 140.2, 135.9, 129.6, 129.1, 128.9, 128.3, 127.0, 126.4, 122.5, 110.2, 107.9, 49.3 (minor) 145.0, 140.8, 129.4, 128.4, 127.5, 126.3, 125.4, 120.1, 119.4, 118.5, 111.6, 50.3 (one peak unobserved). IR (thin film, cm^{-1}) 3084, 3063, 3026, 1600, 1500, 1452, 1325, 1079, 1032, 766, 697. HRMS ($ASAP^+$) exact mass calc'd for $C_{23}H_{20}N$ $[M+H]^+$, requires *m/z* 310.1596, found *m/z* 310.1594.

6. References:

1) Sakai, T.; Seo, S.; Matsuoka, J.; Mori, Y. *J. Org. Chem.* **2013**, 78, 10978.
2) Gheewala, C. D.; Radtke, M. A.; Hui, J.; Hon, A. B.; Lambert, T. H. *Org. Lett.* **2017**, 19, 4227
3) Lebleu, T.; Paquin, J.-F. *Tetrahedron Lett.* **2017**, 58, 442.
4) Okajima, M.; Soga, K.; Nokami, T.; Suga, S.; Yoshida, J.-I. *Org. Lett.* **2006**, 8, 5005.
5) Onodera, G.; Yamamoto, E.; Tonegawa, S.; Iezumi, M.; Takeuchi, R. *Adv. Synth. Catal.* **2011**, 353, 2013.
6) Mayr, H.; Ofial, A.R.; Schimmel, H. *Macromolecules.* **2005**, 38, 33.
7) Hupe, E.; Calaza, M.I.; Knochel, P. *Chem. Eur. J.* **2003**, 9, 2789.
8) Wang, G.-Z.; Jiang, J.; Bu, X.-S.; Dai, J.-J.; Xu, J.; Fu, Y.; Xu, H.-J. *Org. Lett.* **2015**, 17, 3682.
9) Yasuda, M.; Somyo, T.; Baba, A. *Angew. Chem. Int. Ed.* **2006**, 45, 793.
10) Sato, U.; Aoyama, T.; Takido, T.; Kodomari, M. *Tetrahedron* **2012**, 68, 7077.

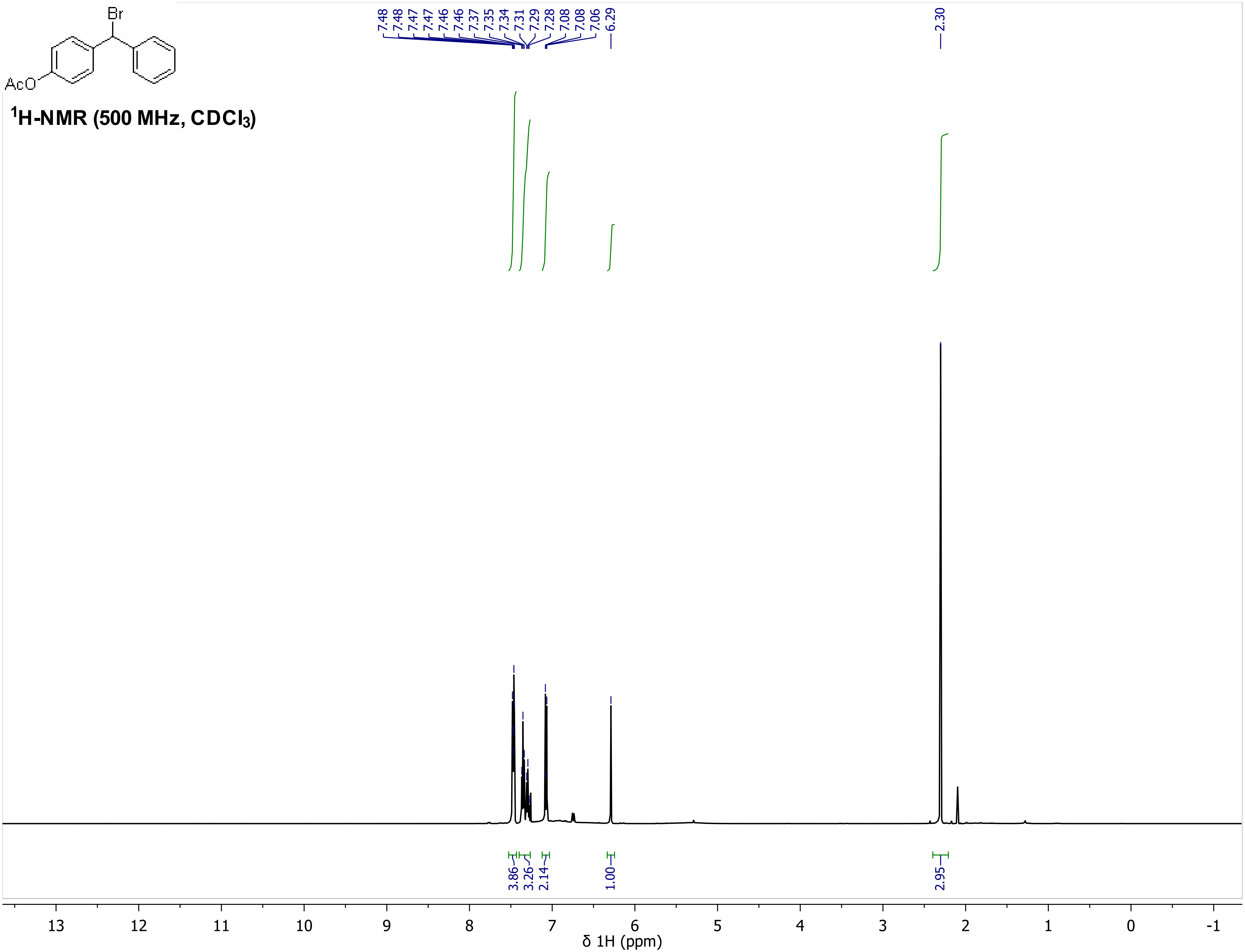
Br
AcO
1H-NMR (500 MHz, CDCl3)
7.48
7.48
7.47
7.47
7.46
7.46
7.37
7.35
7.34
7.31
7.29
7.28
7.08
7.08
7.06
6.29
2.30
3.86
3.26
2.14
1.00
2.95
13
12
11
10
9
8
7
6
5
4
3
2
1
0
-1
δ 1H (ppm)

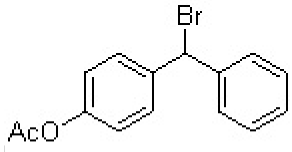

$^{13}C\{^{1}H\}$-NMR (126 MHz, $CDCl_3$)

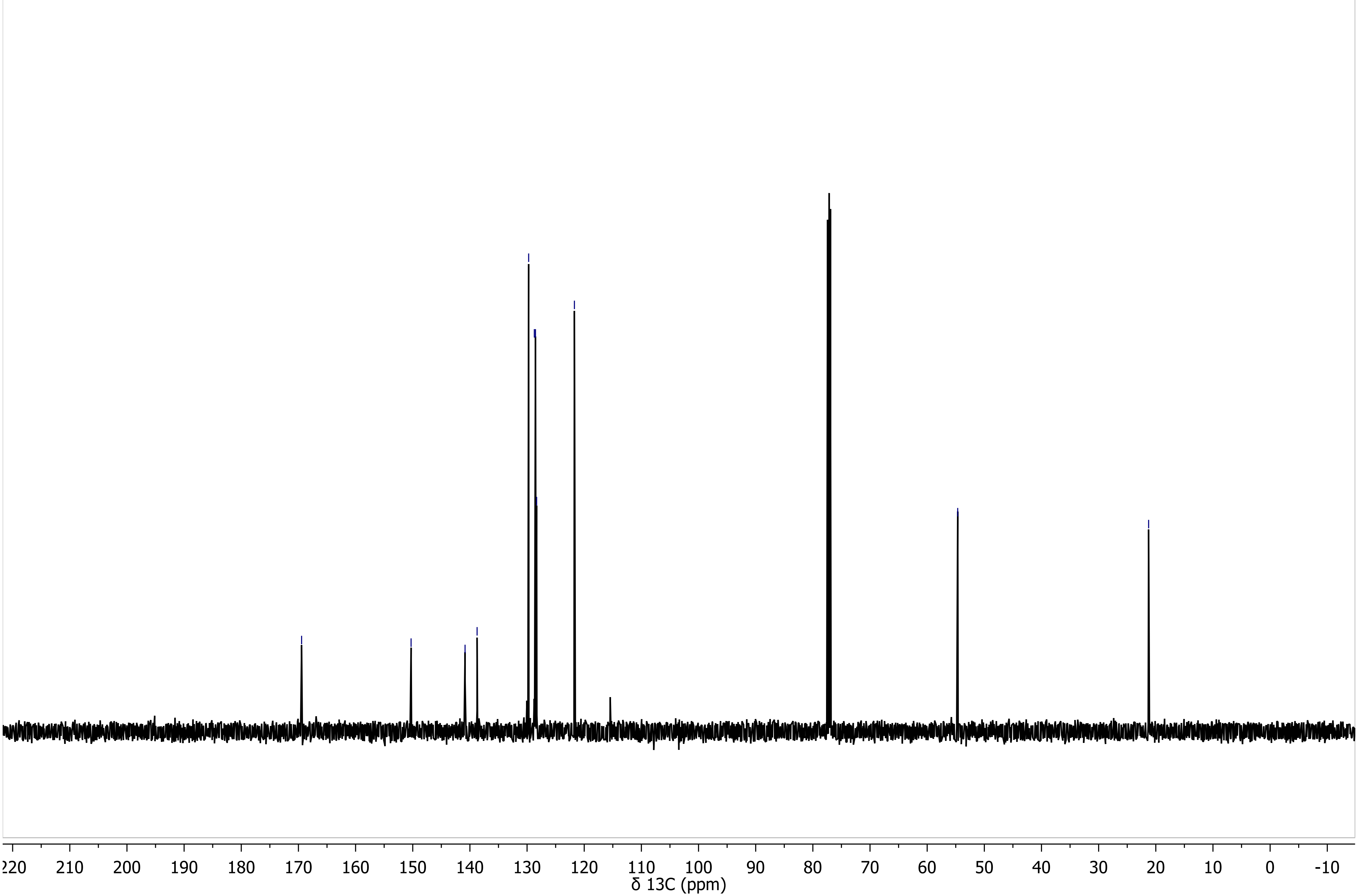

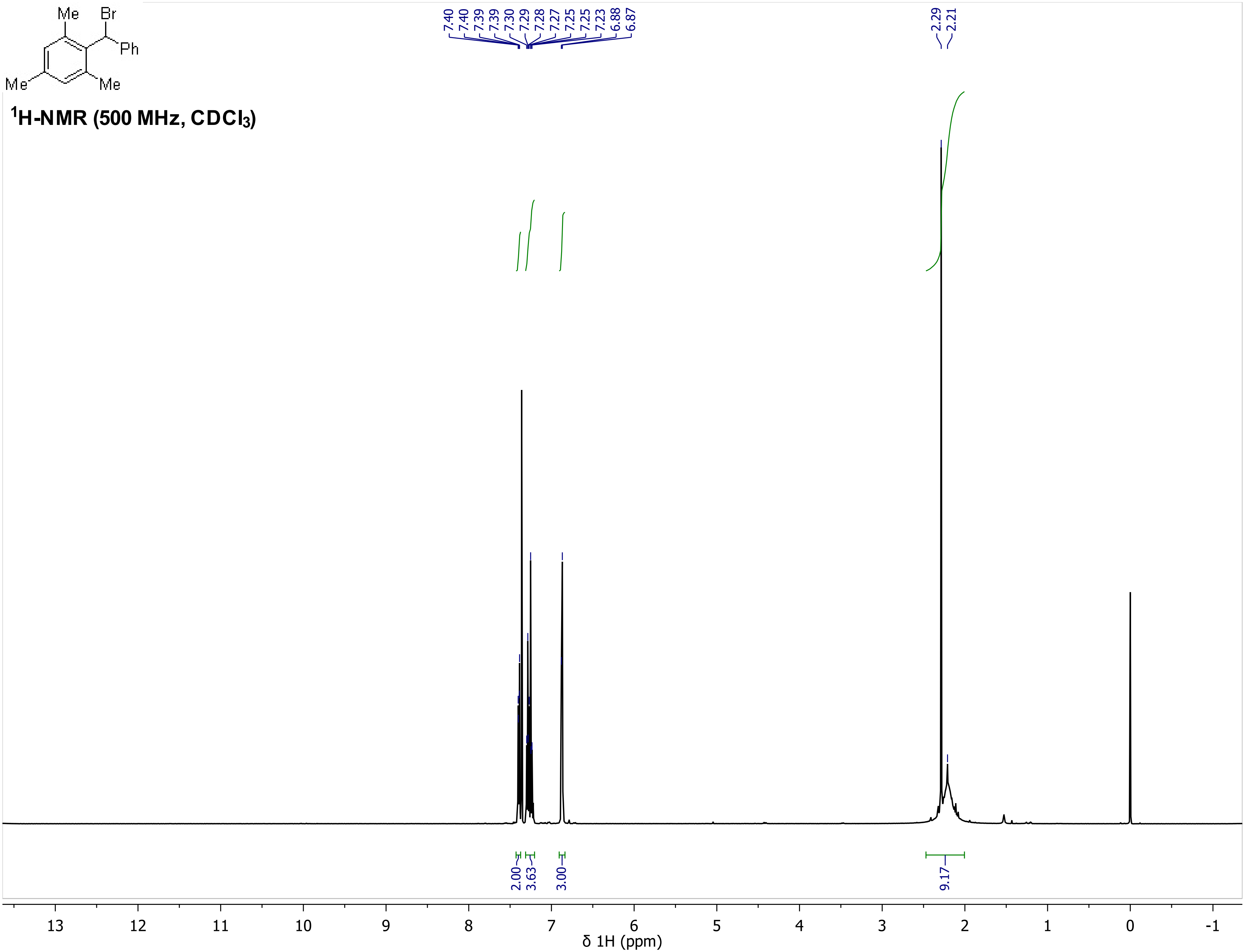

Me
Br
Ph
Me
Me
1H-NMR (500 MHz, CDCl3)
7.40
7.40
7.39
7.39
7.30
7.29
7.28
7.27
7.25
7.25
7.23
6.88
6.87
2.29
2.21
2.00
3.63
3.00
9.17
13
12
11
10
9
8
7
6
5
4
3
2
1
0
-1
δ 1H (ppm)

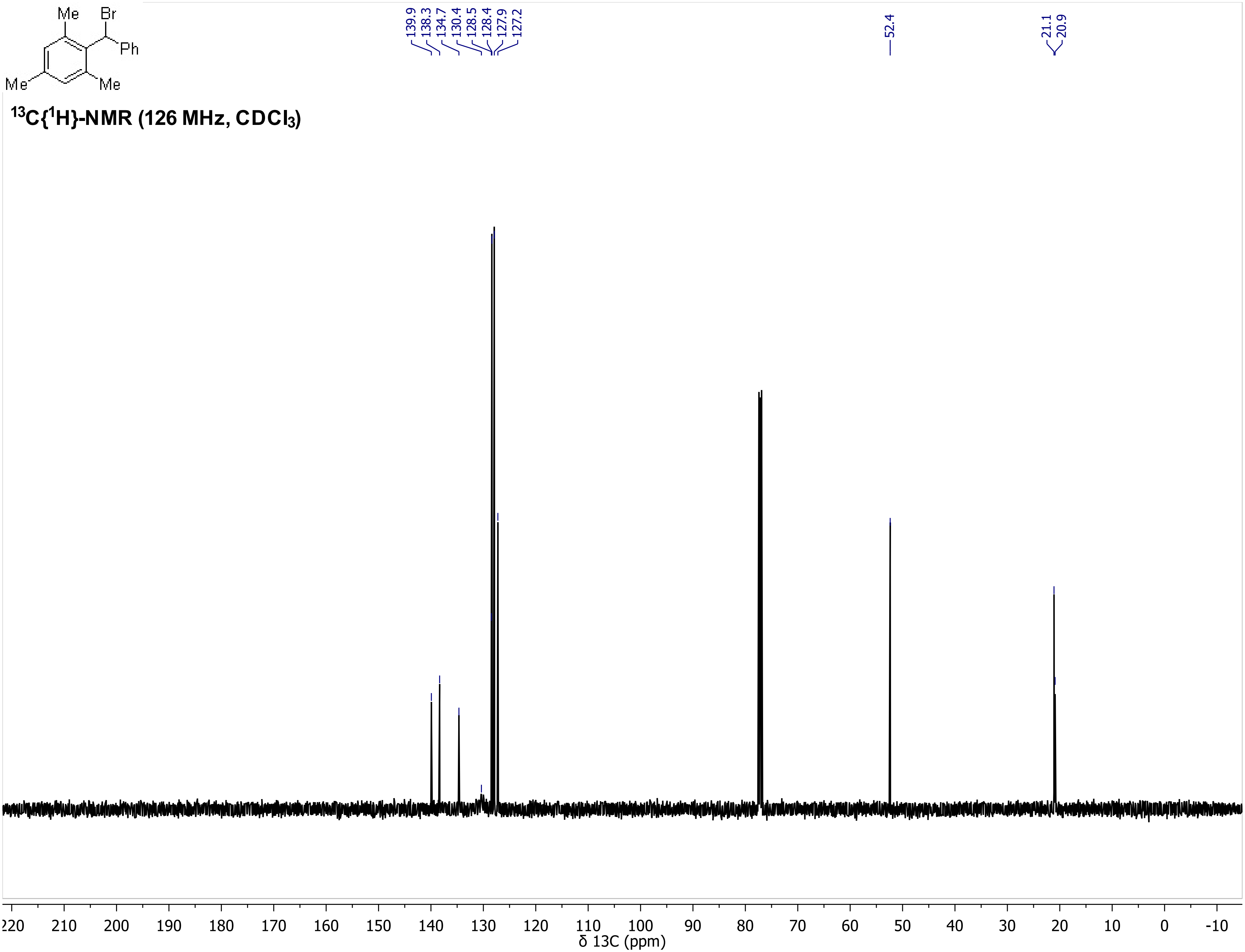

Me
Br
Ph
Me
Me
$^{13}C\{^1H\}$-NMR (126 MHz, $CDCl_3$)
139.9
138.3
134.7
130.4
128.5
128.4
127.9
127.2
52.4
21.1
20.9
220 210 200 190 180 170 160 150 140 130 120 110 100 90 80 70 60 50 40 30 20 10 0 -10
δ 13C (ppm)

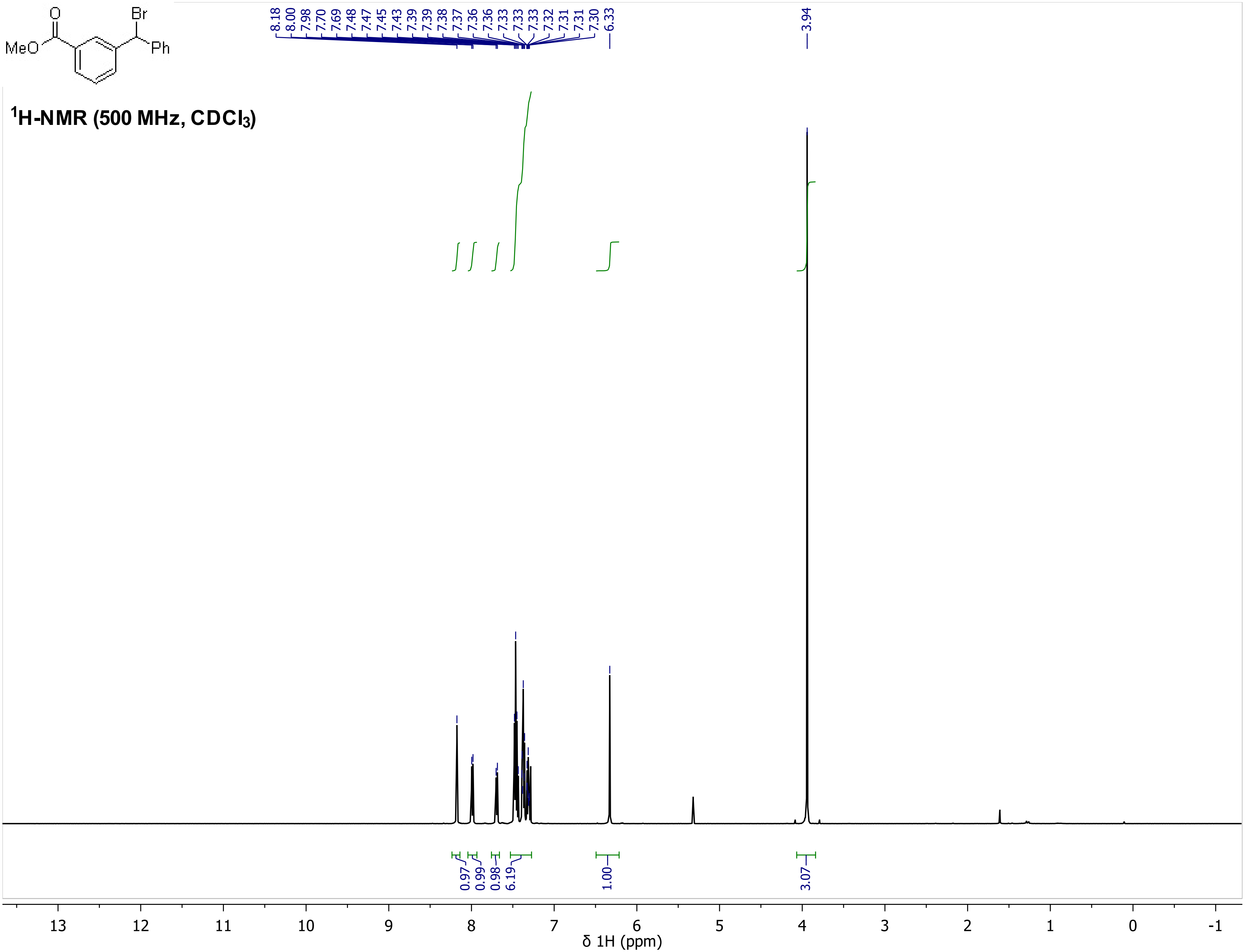

Br
MeO
Ph
1H-NMR (500 MHz, CDCl3)
8.18
8.00
7.98
7.70
7.69
7.48
7.47
7.45
7.43
7.39
7.39
7.38
7.37
7.36
7.36
7.33
7.33
7.33
7.32
7.31
7.31
7.30
6.33
3.94
0.97
0.99
0.98
6.19
1.00
3.07
δ 1H (ppm)

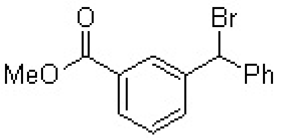

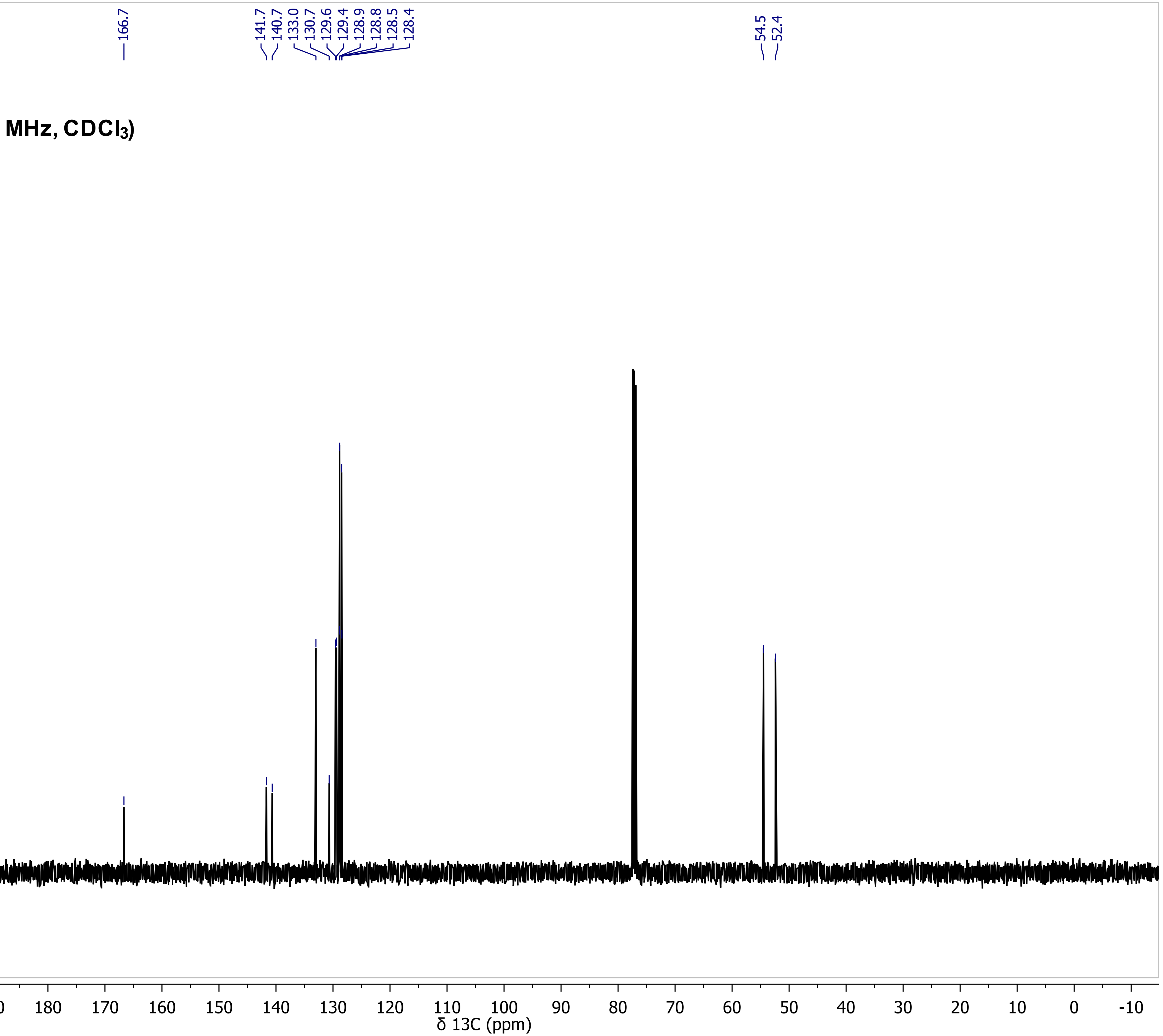

$^{13}C\{^1H\}$-NMR (126 MHz, $CDCl_3$)

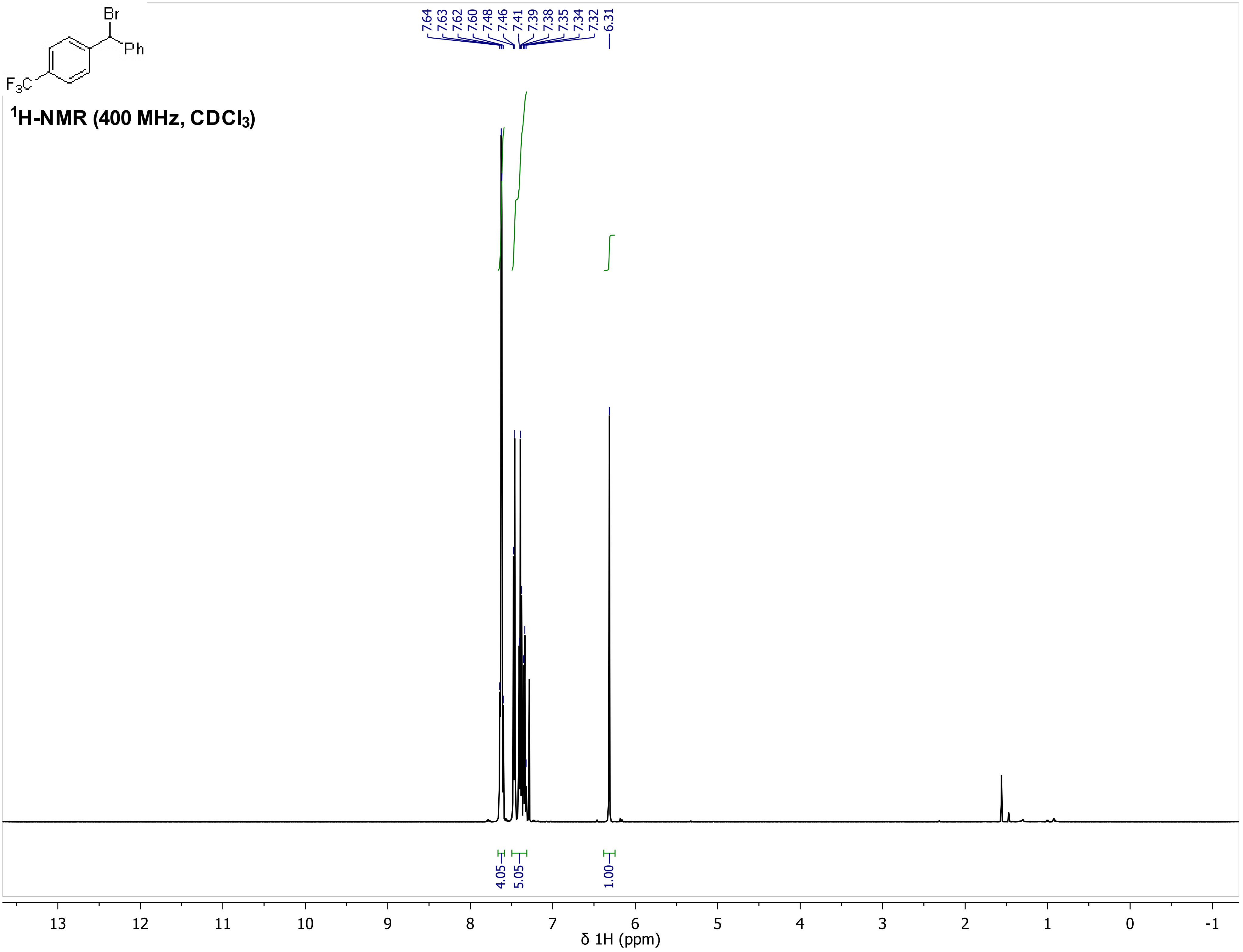

Br
Ph
F_3C
^{1}H-NMR (400 MHz, $CDCl_3$)
7.64
7.63
7.62
7.60
7.48
7.46
7.41
7.39
7.38
7.35
7.34
7.32
6.31
4.05
5.05
1.00
13 12 11 10 9 8 7 6 5 4 3 2 1 0 -1
δ 1H (ppm)

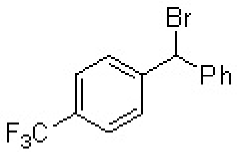

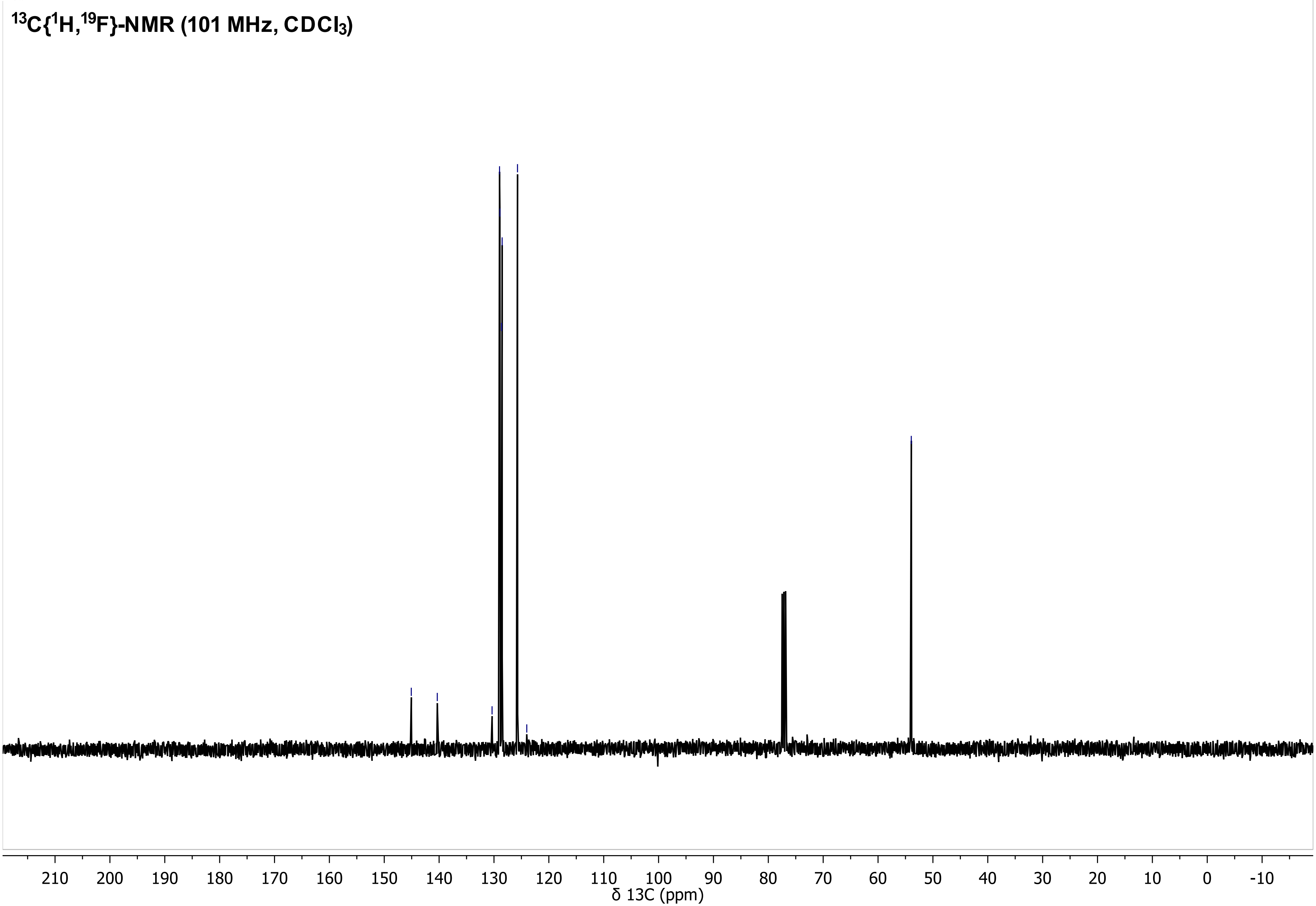

13C{1H,19F}-NMR (101 MHz, CDCl3)
145.07
140.33
130.35
128.99
128.92
128.61
128.51
125.71
124.02
53.94
210
200
190
180
170
160
150
140
130
120
110
100
90
80
70
60
50
40
30
20
10
0
-10
δ 13C (ppm)

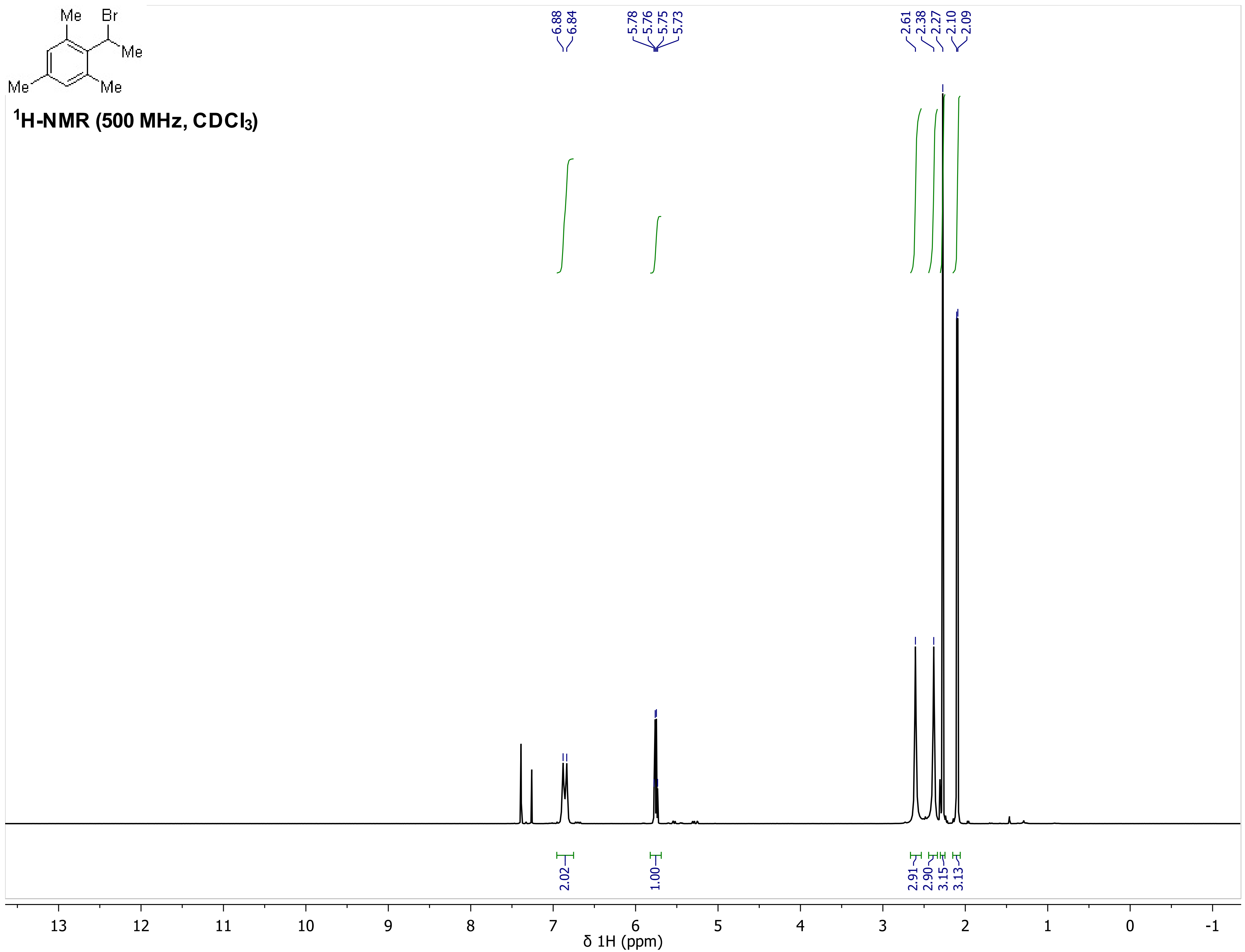

Me
Br
Me
Me
Me
¹H-NMR (500 MHz, CDCl₃)
6.88
6.84
5.78
5.76
5.75
5.73
2.61
2.38
2.27
2.10
2.09
2.02
1.00
2.91
2.90
3.15
3.13
13
12
11
10
9
8
7
6
5
4
3
2
1
0
-1
δ 1H (ppm)

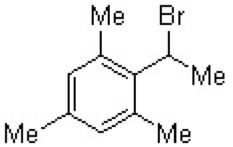

$^{13}C\{^1H\}$-NMR (126 MHz, $CDCl_3$)

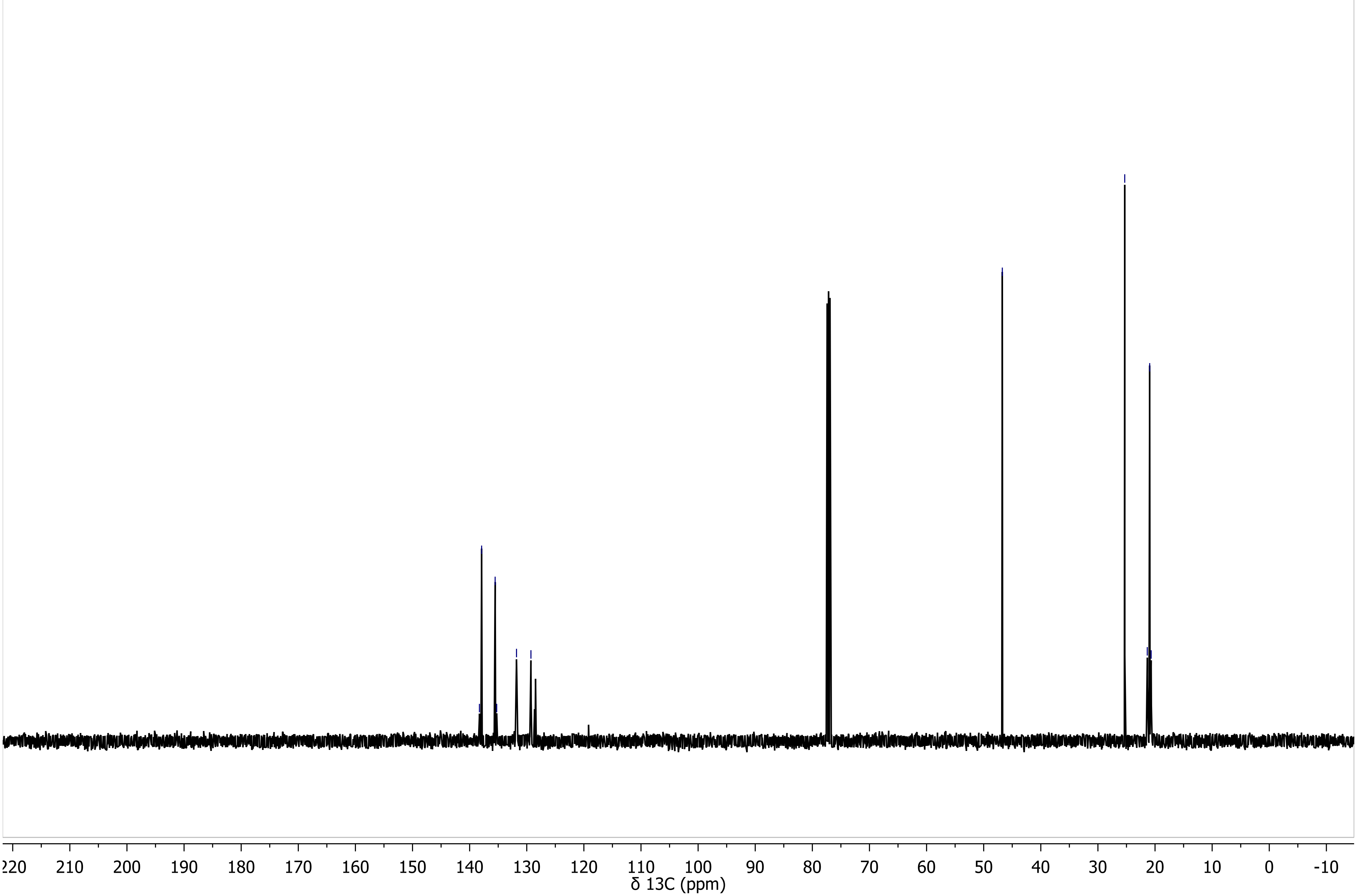

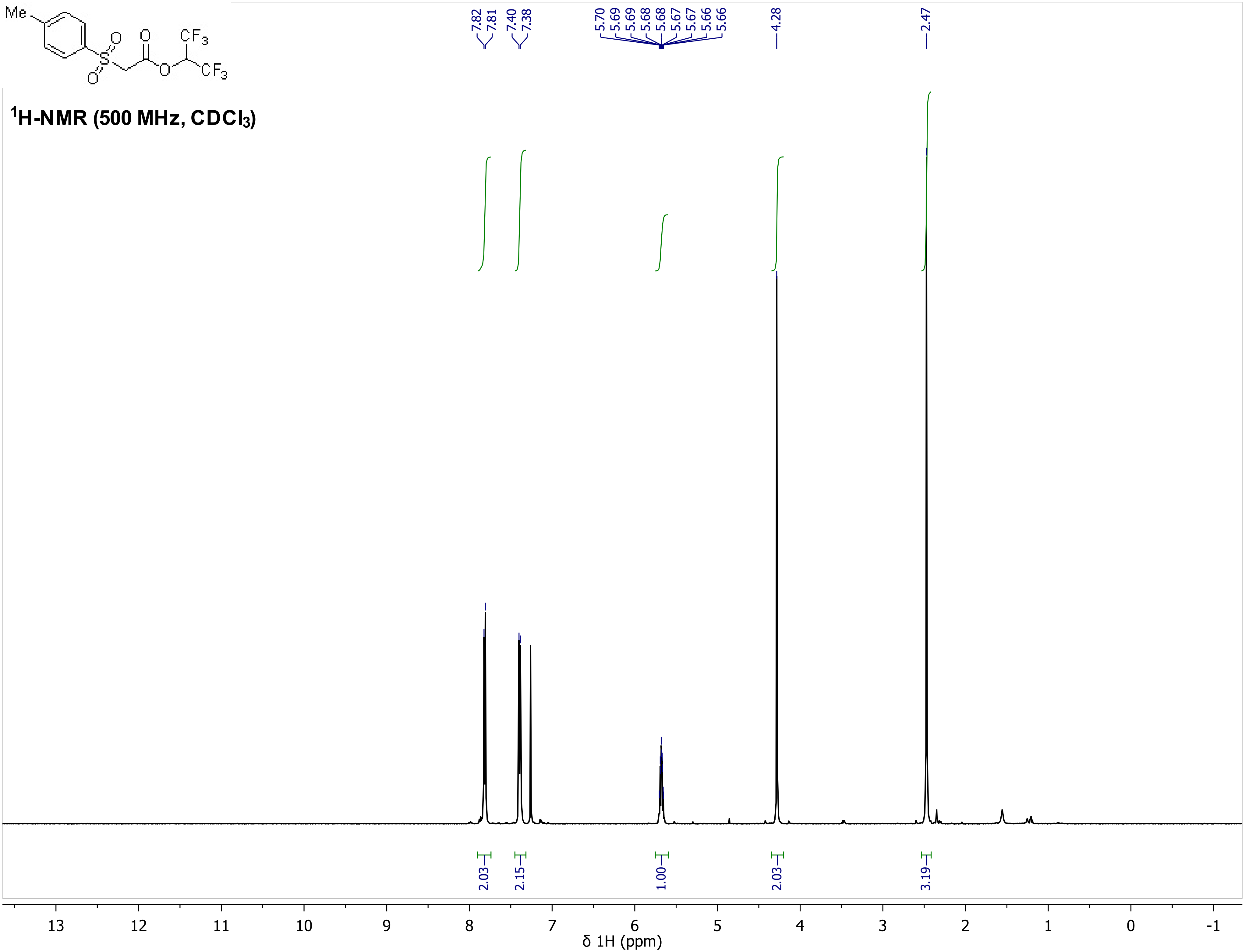

Me
O
O
CF3
CF3
S
O
O
¹H-NMR (500 MHz, CDCl3)
7.82
7.81
7.40
7.38
5.70
5.69
5.69
5.68
5.68
5.67
5.67
5.66
5.66
4.28
2.47
2.03
2.15
1.00
2.03
3.19
13
12
11
10
9
8
7
6
5
4
3
2
1
0
-1
δ 1H (ppm)

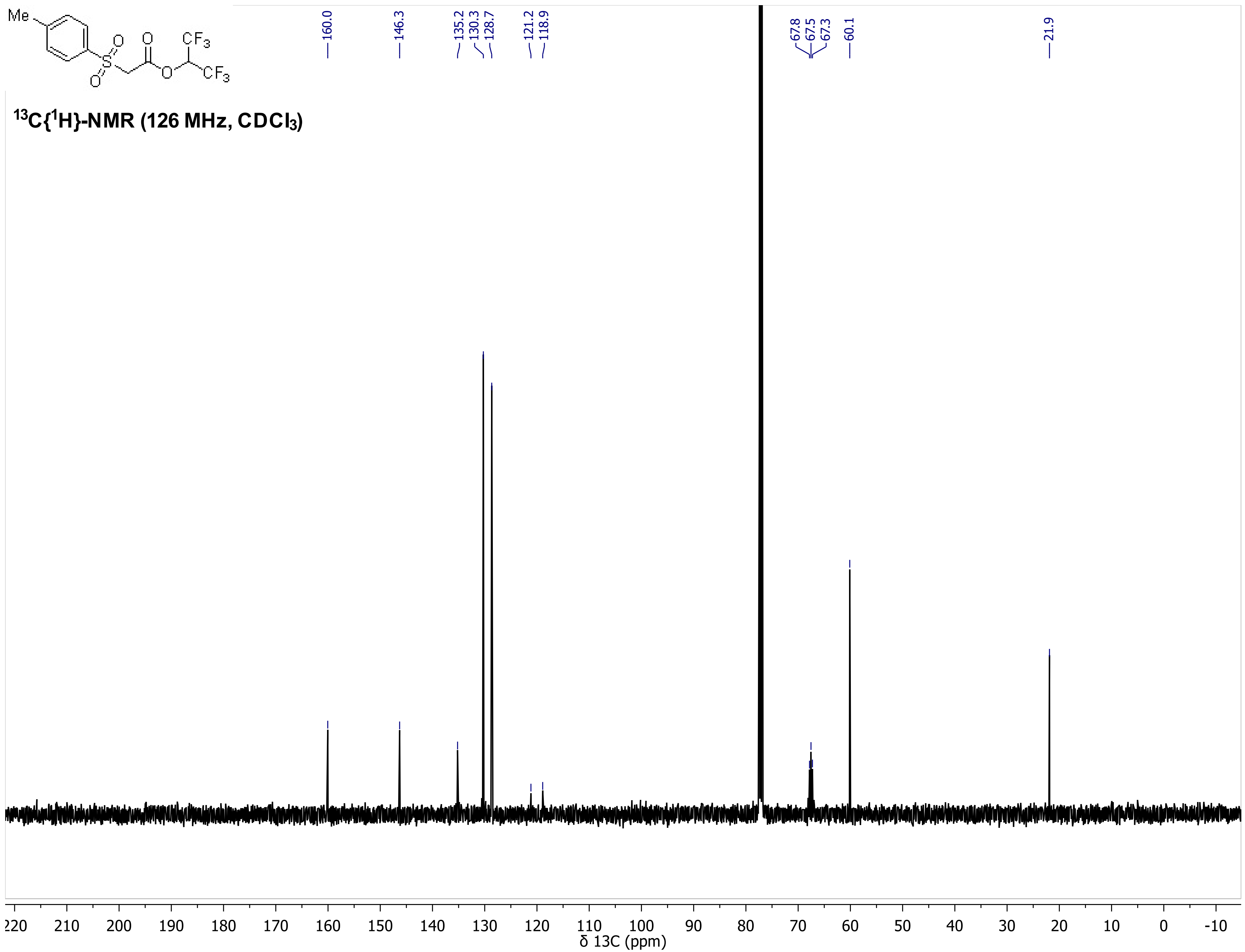

Me
O
O
CF3
CF3
O
S
O
13C{1H}-NMR (126 MHz, CDCl3)
160.0
146.3
135.2
130.3
128.7
121.2
118.9
67.8
67.5
67.3
60.1
21.9
220
210
200
190
180
170
160
150
140
130
120
110
100
90
80
70
60
50
40
30
20
10
0
-10
δ 13C (ppm)

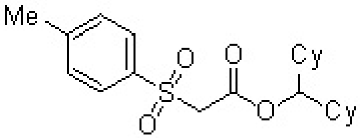

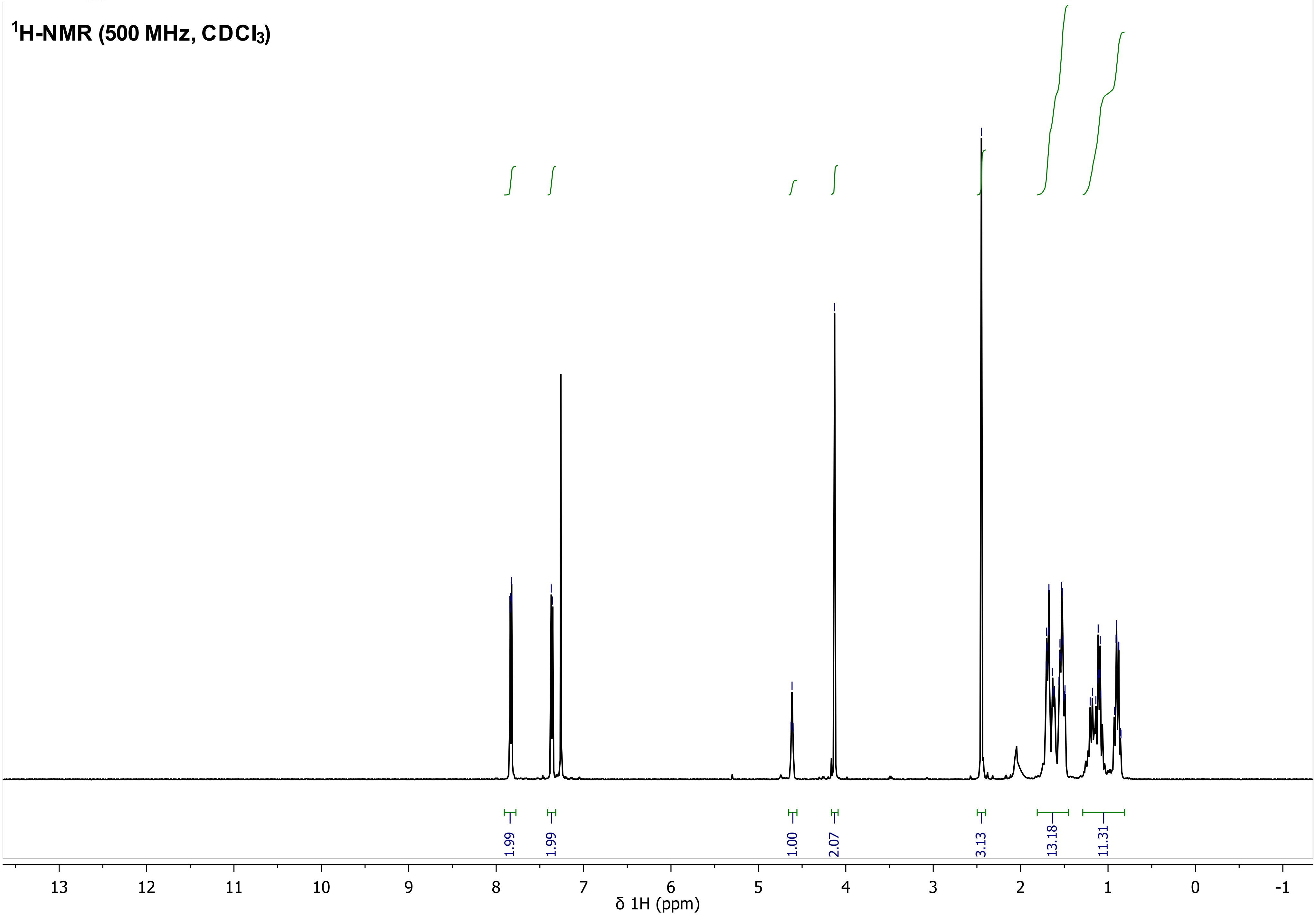

1H-NMR (500 MHz, CDCl3)
δ 1H (ppm)

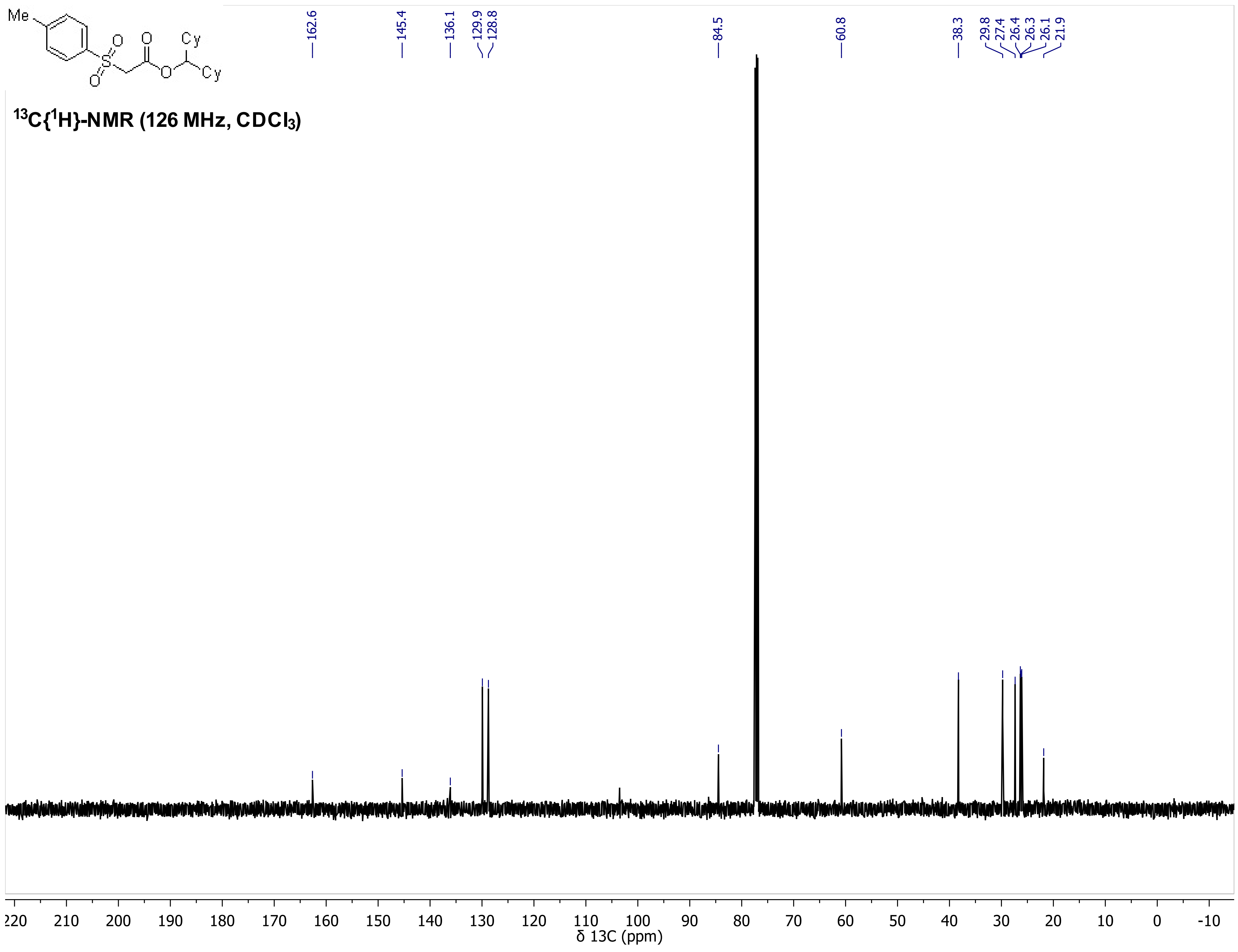

Me
O
O
O
Cy
O
Cy
S
^{13}C{^{1}H}-NMR (126 MHz, CDCl$_3$)
162.6
145.4
136.1
129.9
128.8
84.5
60.8
38.3
29.8
27.4
26.4
26.3
26.1
21.9
220 210 200 190 180 170 160 150 140 130 120 110 100 90 80 70 60 50 40 30 20 10 0 -10
δ 13C (ppm)

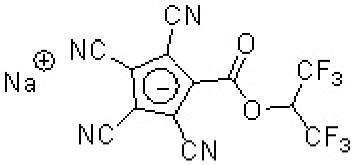

^{1}H-NMR (400 MHz, CD_3CN)

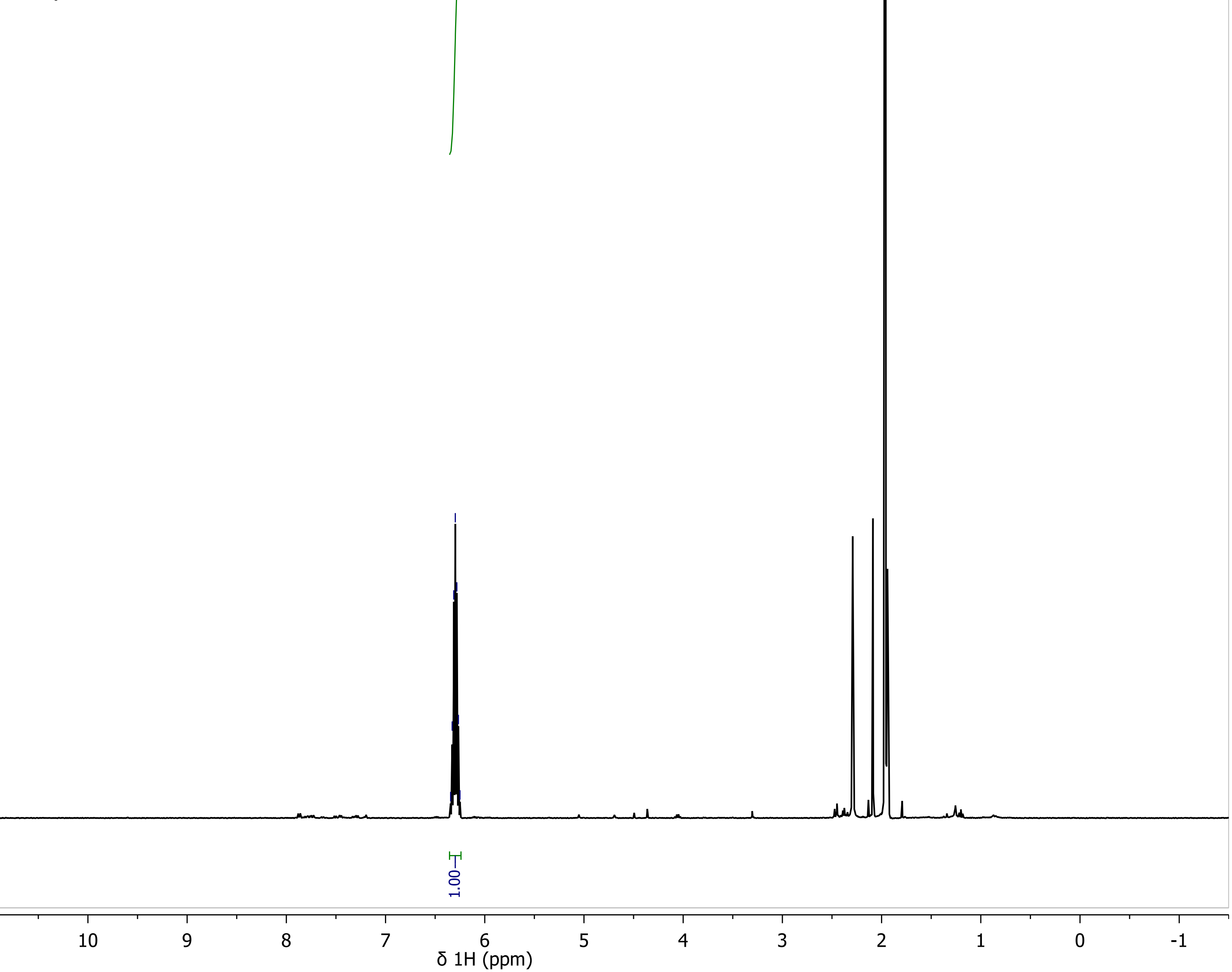

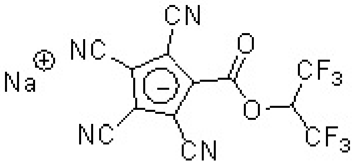

$^{13}C\{^{1}H,^{19}F\}$-NMR (101 MHz, CD_3CN)

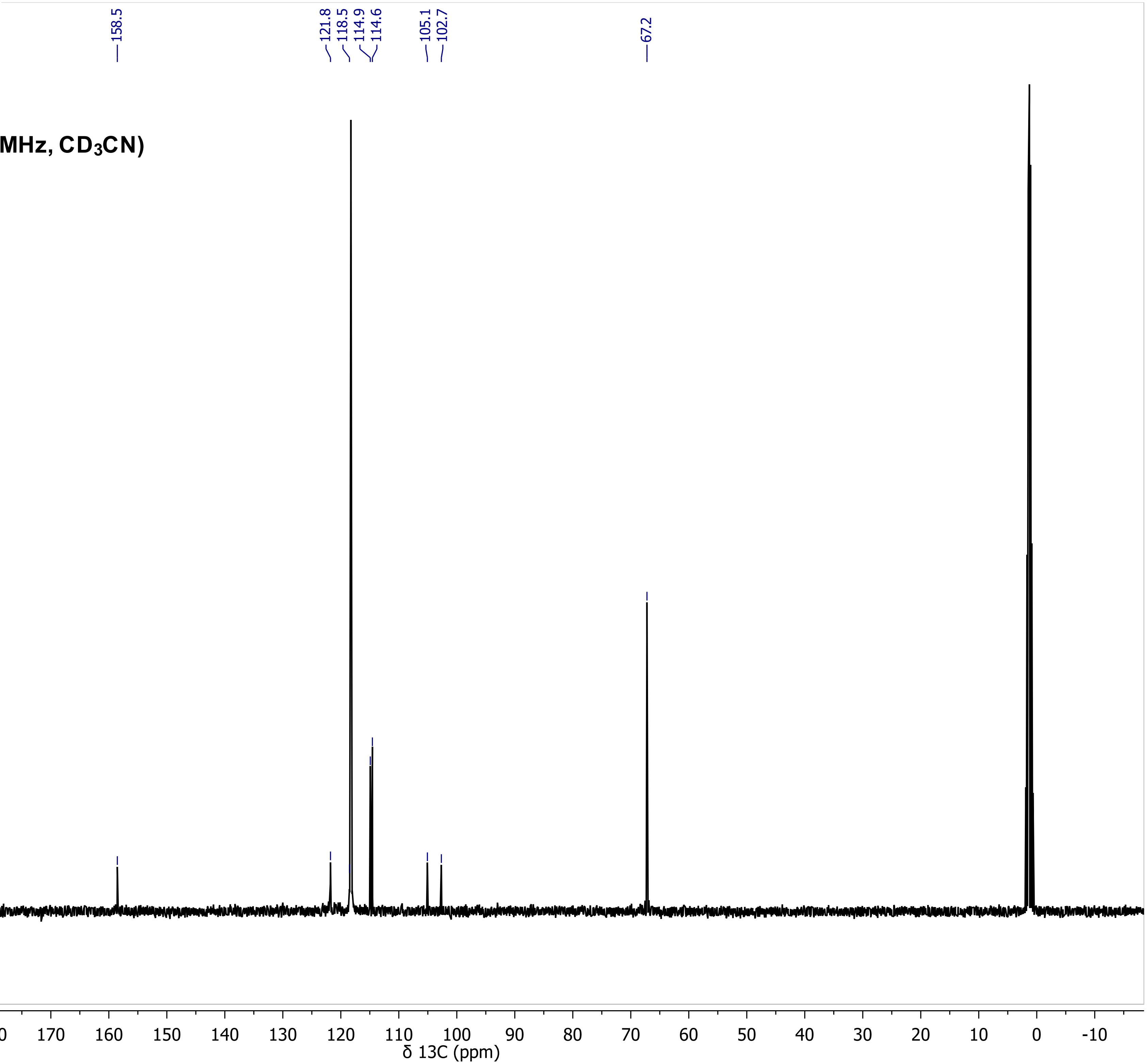

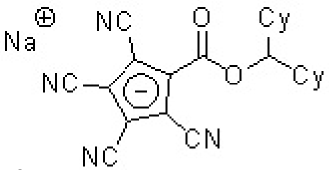

Na
NC
O
Cy
NC
O
Cy
NC
CN

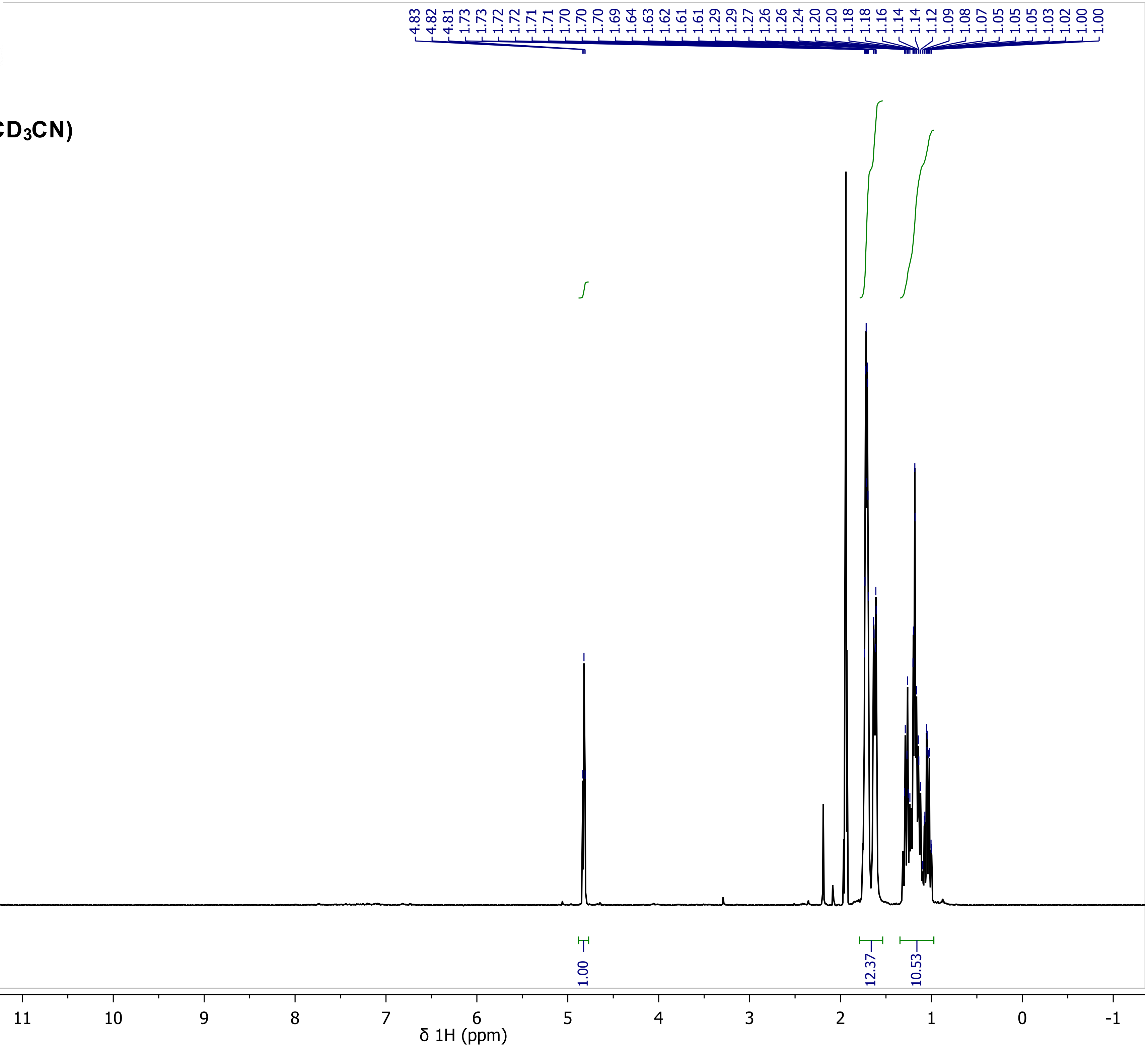

^{1}H-NMR (500 MHz, CD_3CN)
4.83
4.82
4.81
1.73
1.73
1.72
1.72
1.71
1.71
1.70
1.70
1.70
1.69
1.64
1.63
1.62
1.61
1.61
1.29
1.29
1.27
1.26
1.26
1.24
1.20
1.20
1.18
1.18
1.16
1.14
1.14
1.12
1.09
1.08
1.07
1.05
1.05
1.05
1.03
1.02
1.00
1.00
1.00
12.37
10.53
13
12
11
10
9
8
7
6
5
4
3
2
1
0
-1
δ 1H (ppm)

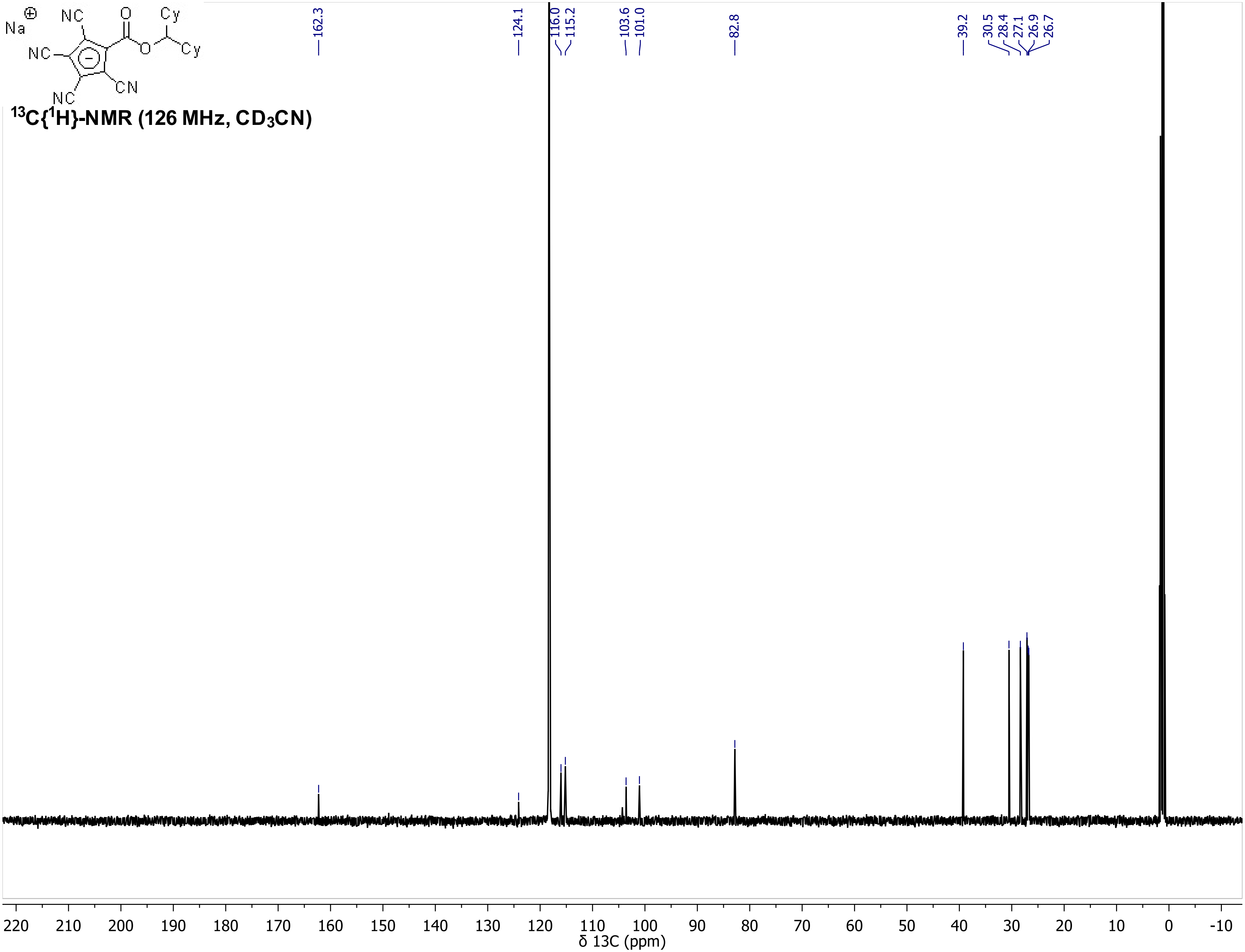

Na⊕
NC
O
Cy
NC
O
Cy
CN
NC
$^{13}C\{^{1}H\}$-NMR (126 MHz, CD_3CN)
162.3
124.1
116.0
115.2
103.6
101.0
82.8
39.2
30.5
28.4
27.1
26.9
26.7
220 210 200 190 180 170 160 150 140 130 120 110 100 90 80 70 60 50 40 30 20 10 0 -10
δ 13C (ppm)

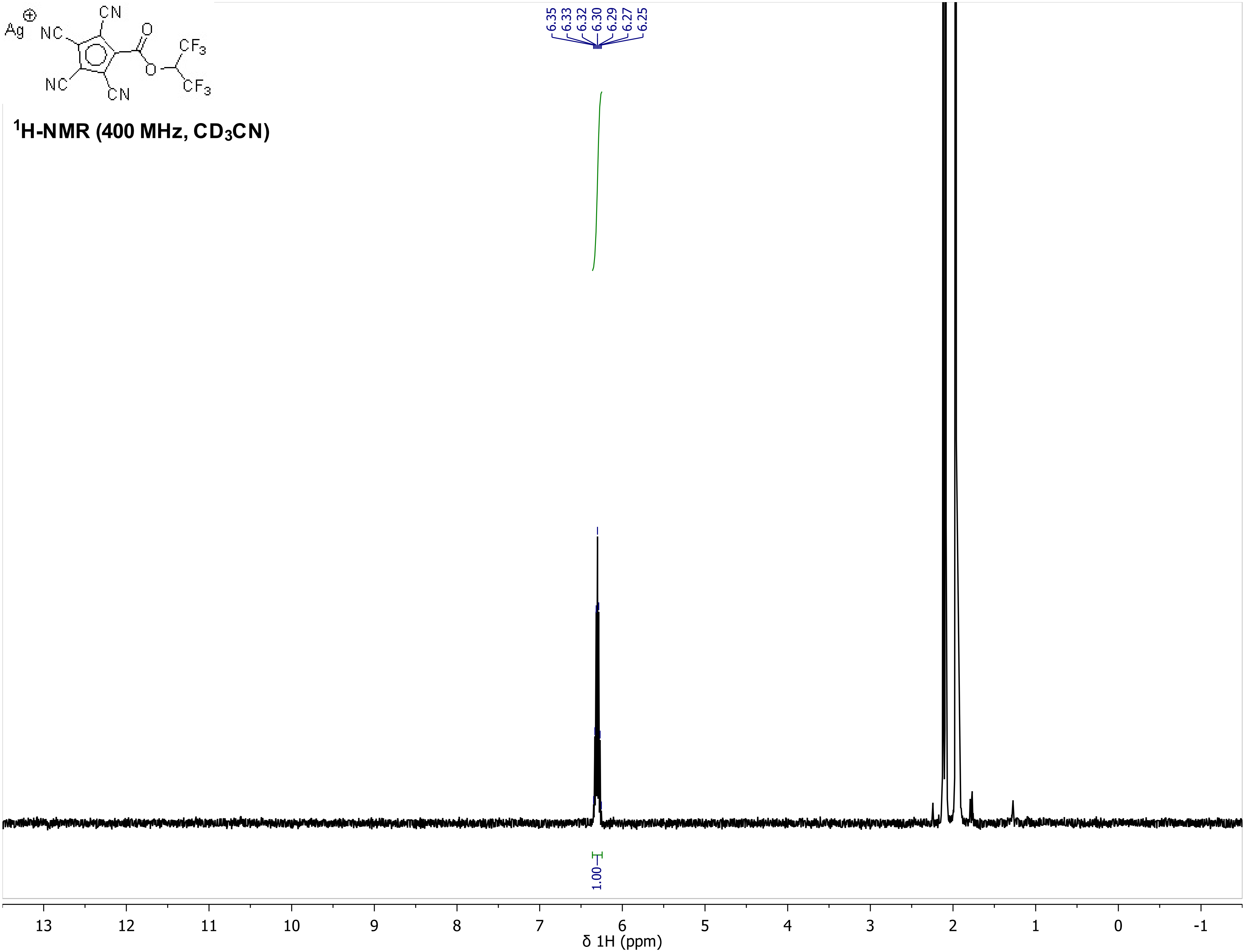

Ag
NC
CN
O
CF3
O
NC
CN
CF3
1H-NMR (400 MHz, CD3CN)
6.35
6.33
6.32
6.30
6.29
6.27
6.25
1.00
13
12
11
10
9
8
7
6
5
4
3
2
1
0
-1
δ 1H (ppm)

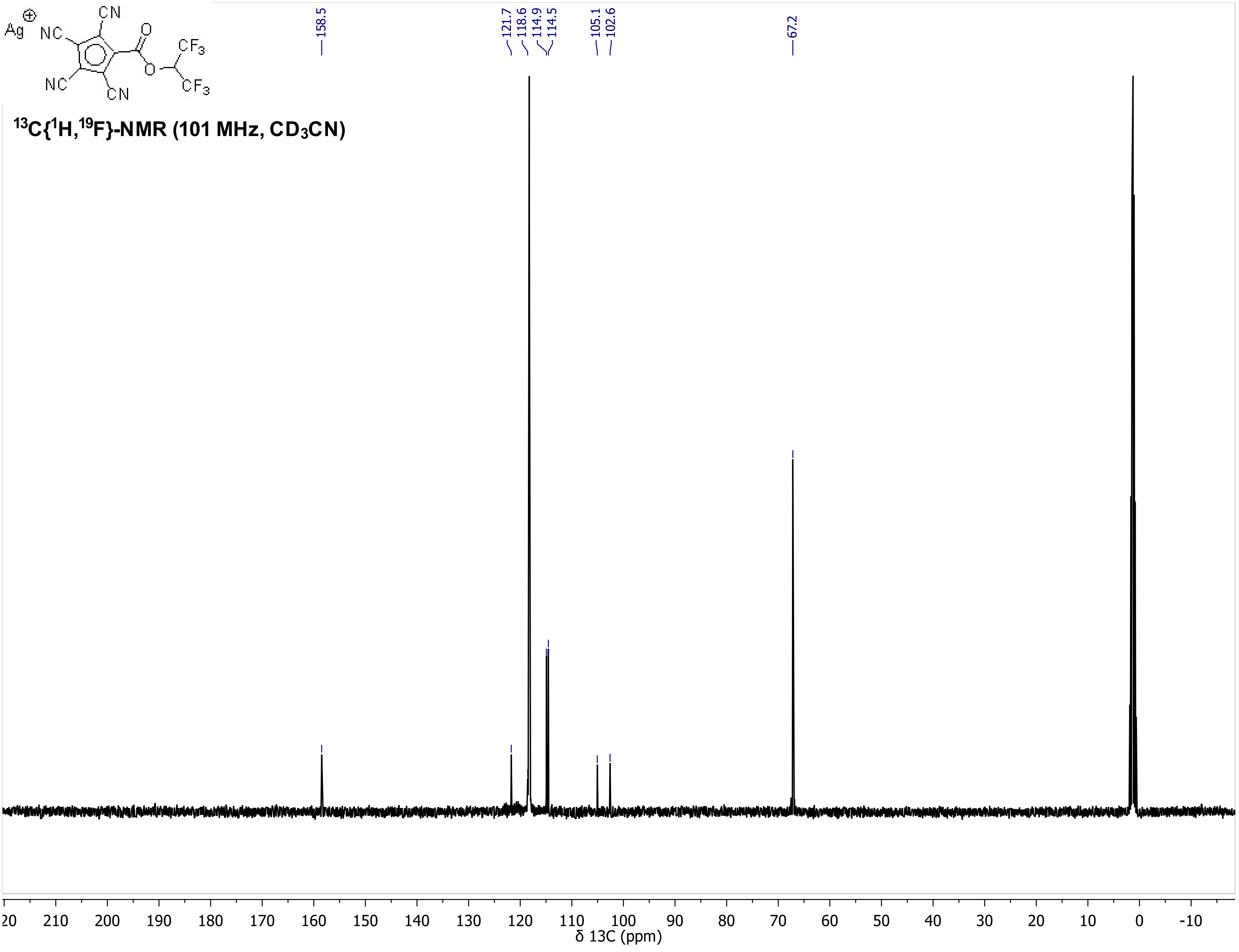

13C{1H,19F}-NMR (101 MHz, CD3CN)
Ag
NC
CN
O
CF3
O
NC
CN
CF3
158.5
121.7
118.6
114.9
114.5
105.1
102.6
67.2
20
210
200
190
180
170
160
150
140
130
120
110
100
90
80
70
60
50
40
30
20
10
0
-10
δ 13C (ppm)

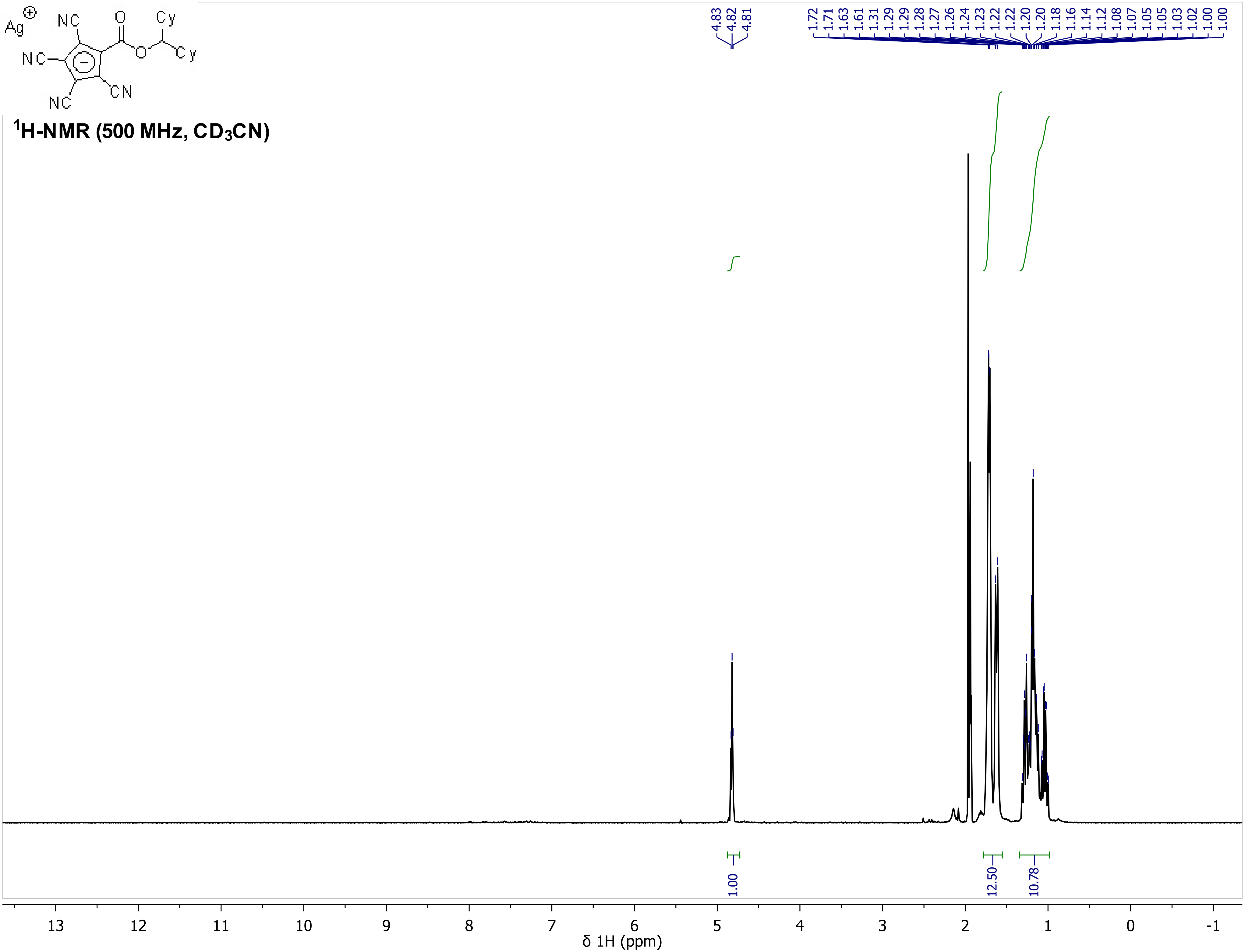

Ag
NC
NC
NC
CN
O
O
Cy
Cy
¹H-NMR (500 MHz, CD₃CN)
4.83
4.82
4.81
1.72
1.71
1.63
1.61
1.31
1.29
1.29
1.28
1.27
1.26
1.24
1.23
1.22
1.22
1.20
1.20
1.18
1.16
1.14
1.12
1.08
1.07
1.05
1.05
1.03
1.02
1.00
1.00
1.00
12.50
10.78
δ 1H (ppm)

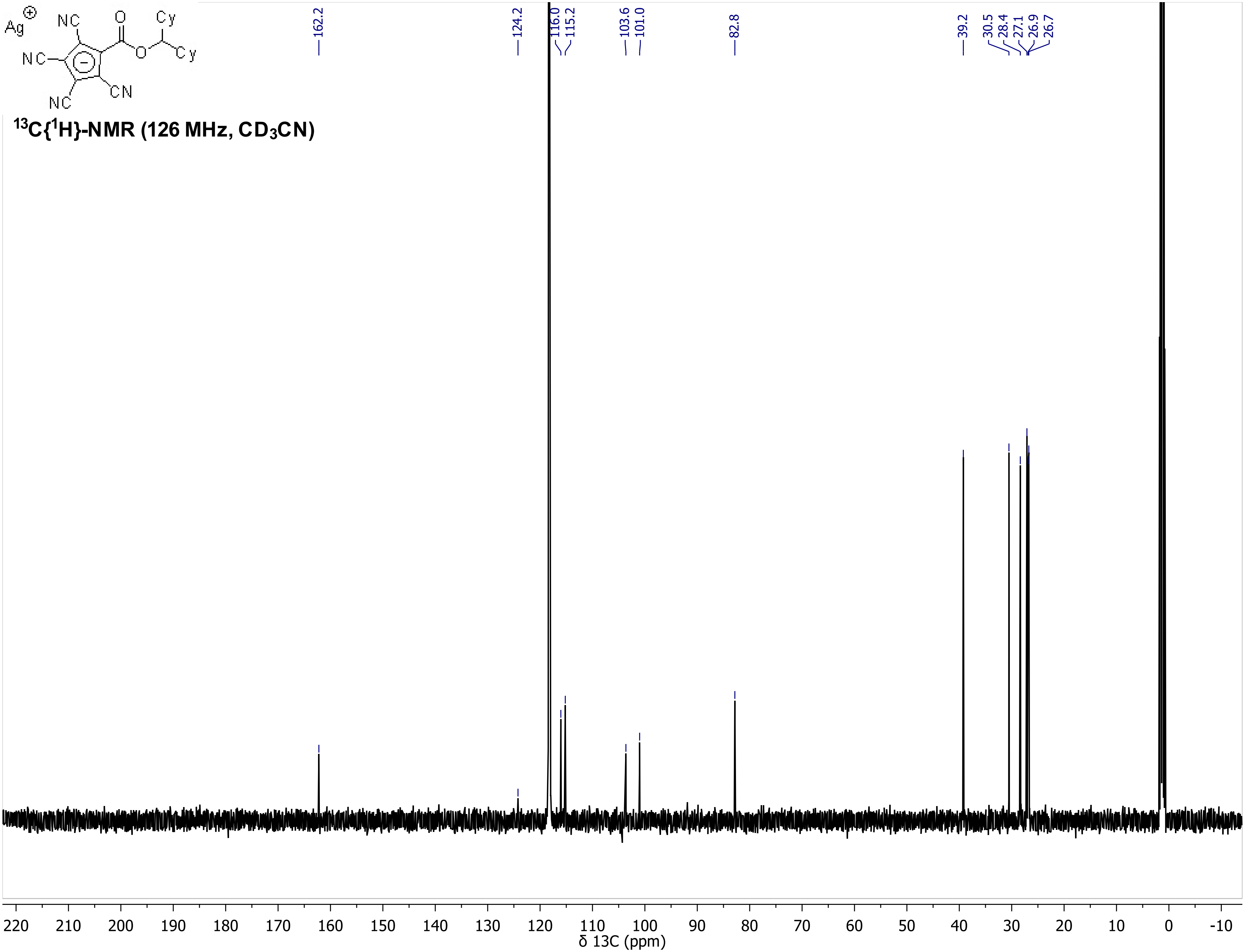

Ag
NC
NC
NC
CN
O
O
Cy
Cy
$^{13}C\{^1H\}$-NMR (126 MHz, CD_3CN)
162.2
124.2
116.0
115.2
103.6
101.0
82.8
39.2
30.5
28.4
27.1
26.9
26.7
220 210 200 190 180 170 160 150 140 130 120 110 100 90 80 70 60 50 40 30 20 10 0 -10
δ 13C (ppm)

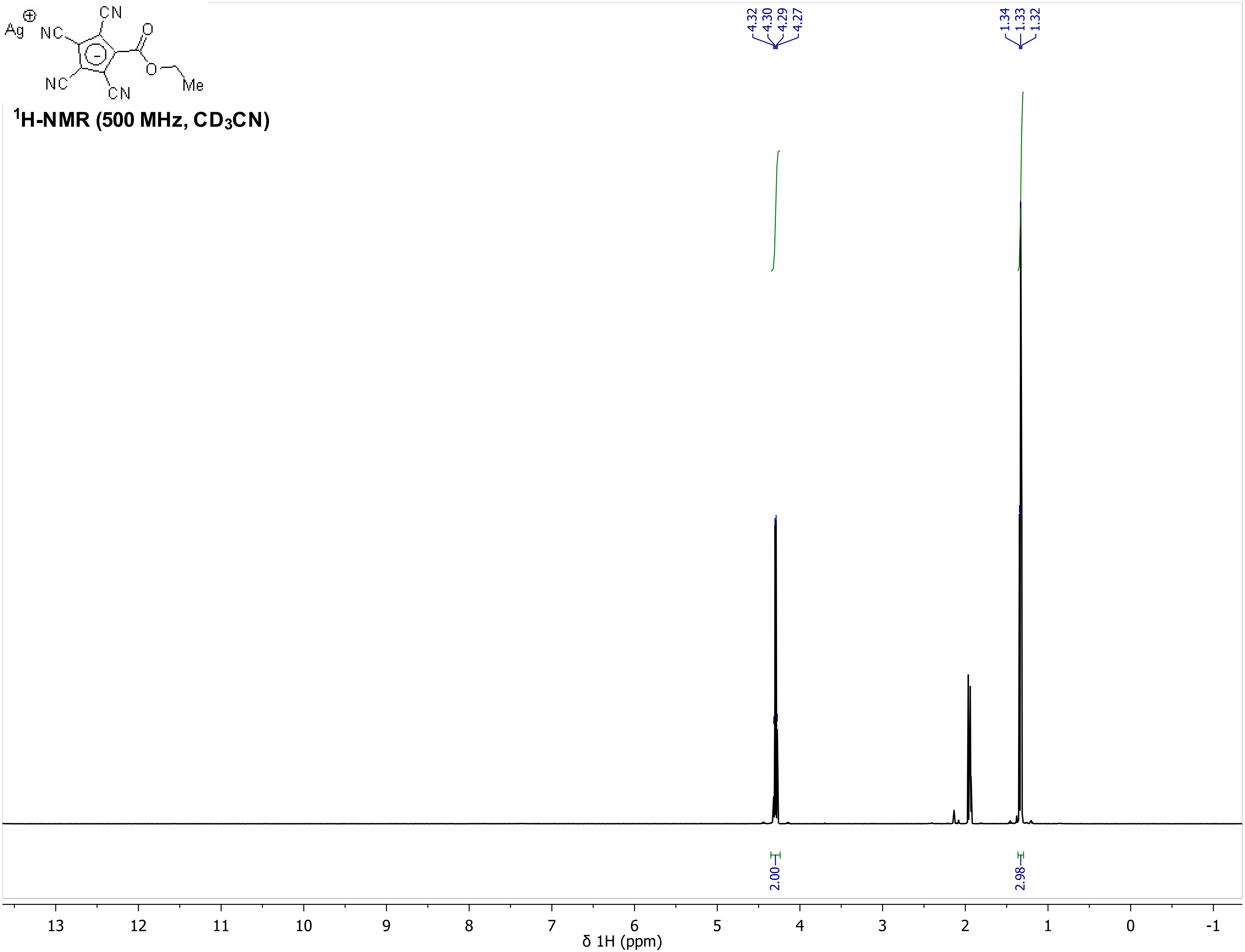

Ag
NC
CN
O
O
Me
NC
CN
1H-NMR (500 MHz, CD3CN)
4.32
4.30
4.29
4.27
1.34
1.33
1.32
2.00
2.98
13
12
11
10
9
8
7
6
5
4
3
2
1
0
-1
δ 1H (ppm)

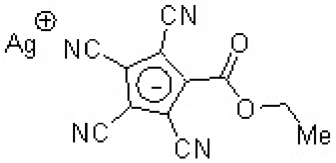

$^{13}C\{^{1}H\}$-NMR (126 MHz, CD_3CN)

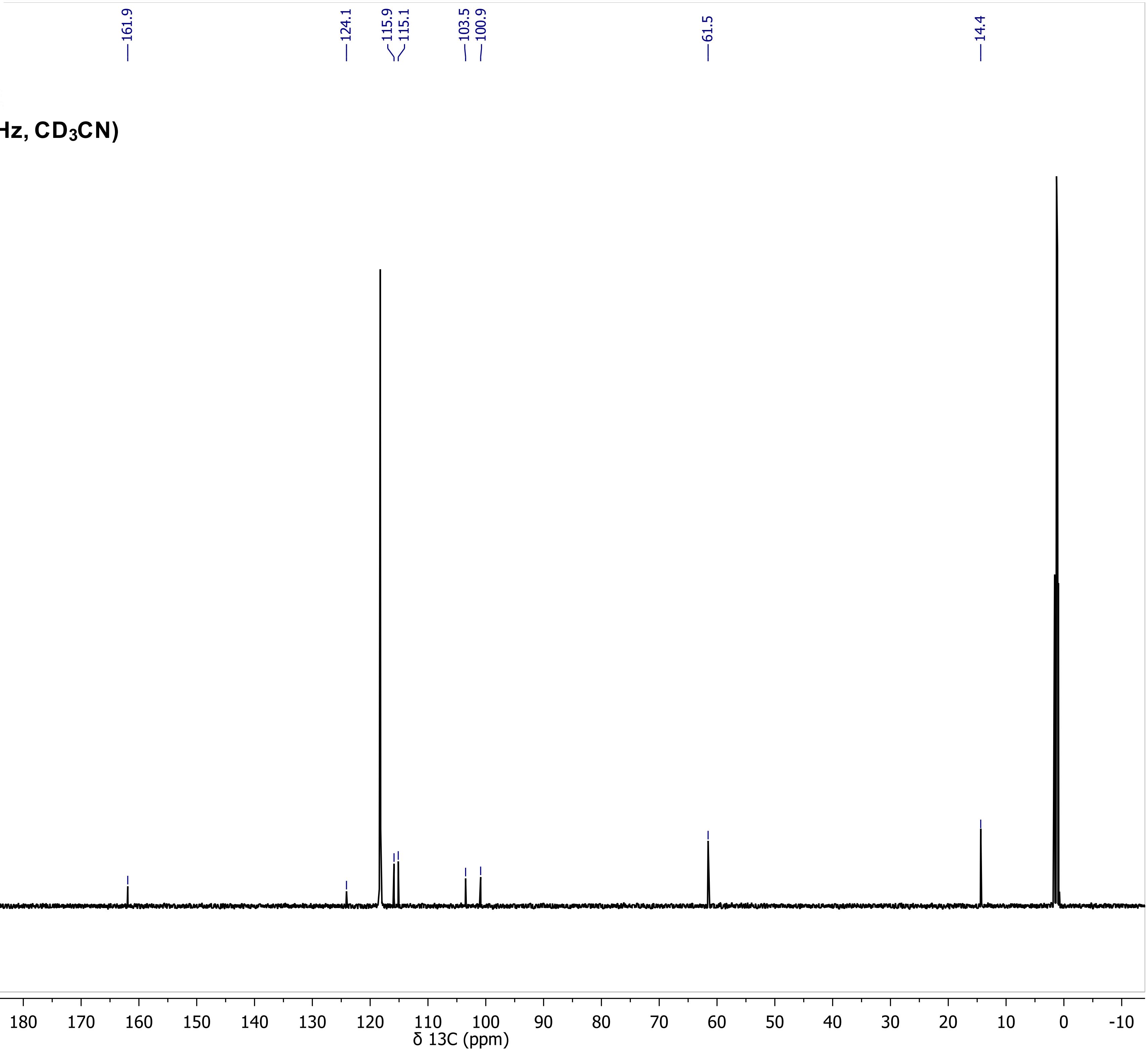

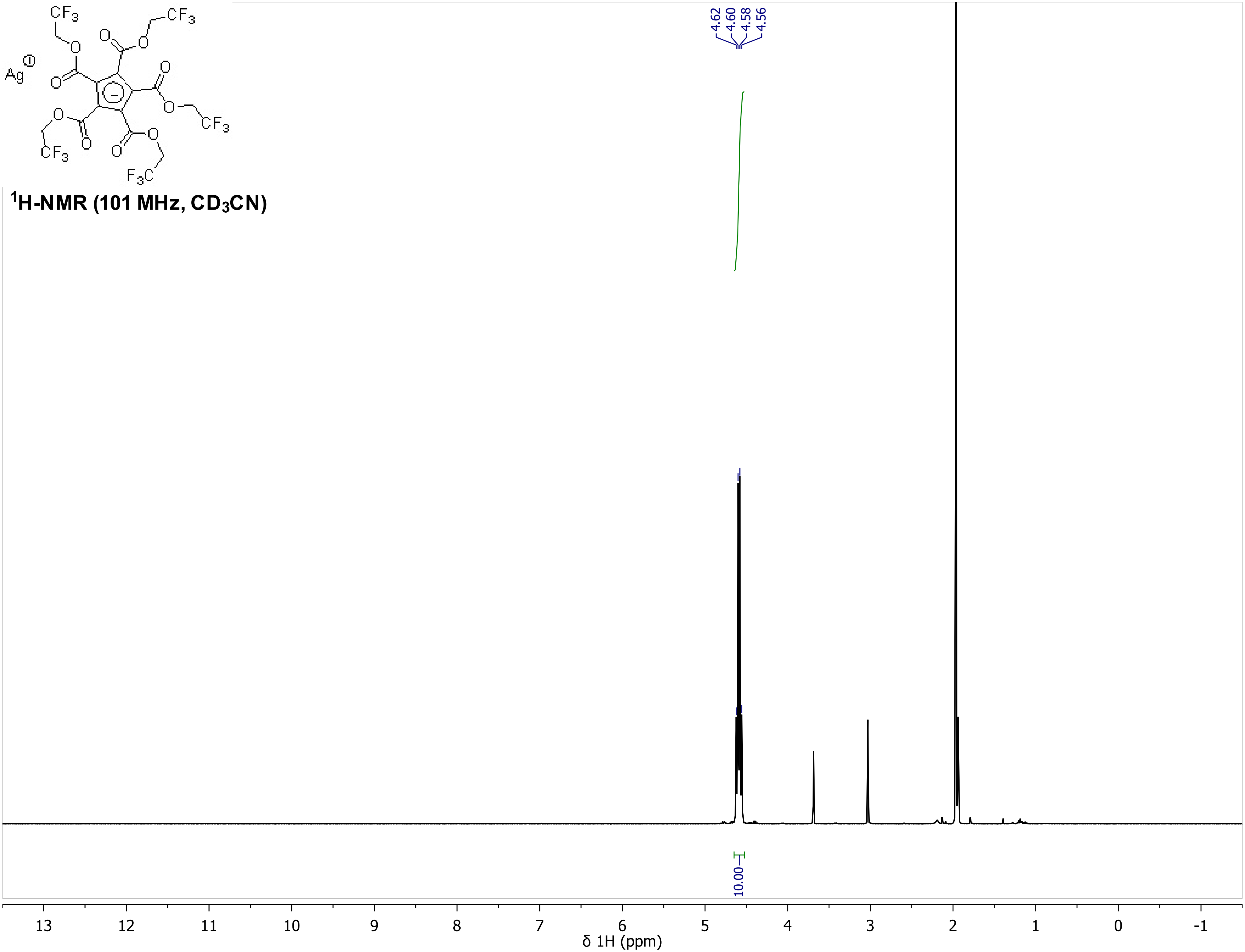

Ag
CF3
O
^{1}H-NMR (101 MHz, CD$_3$CN)
4.62
4.60
4.58
4.56
10.00
13
12
11
10
9
8
7
6
5
4
3
2
1
0
-1
δ 1H (ppm)

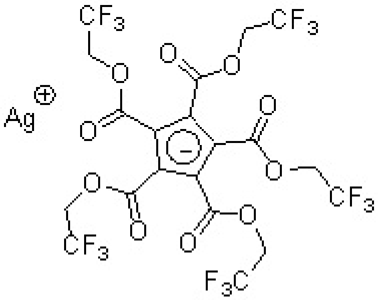

$^{13}C\{^{1}H,^{19}F\}$-NMR (101 MHz, CD_3CN)

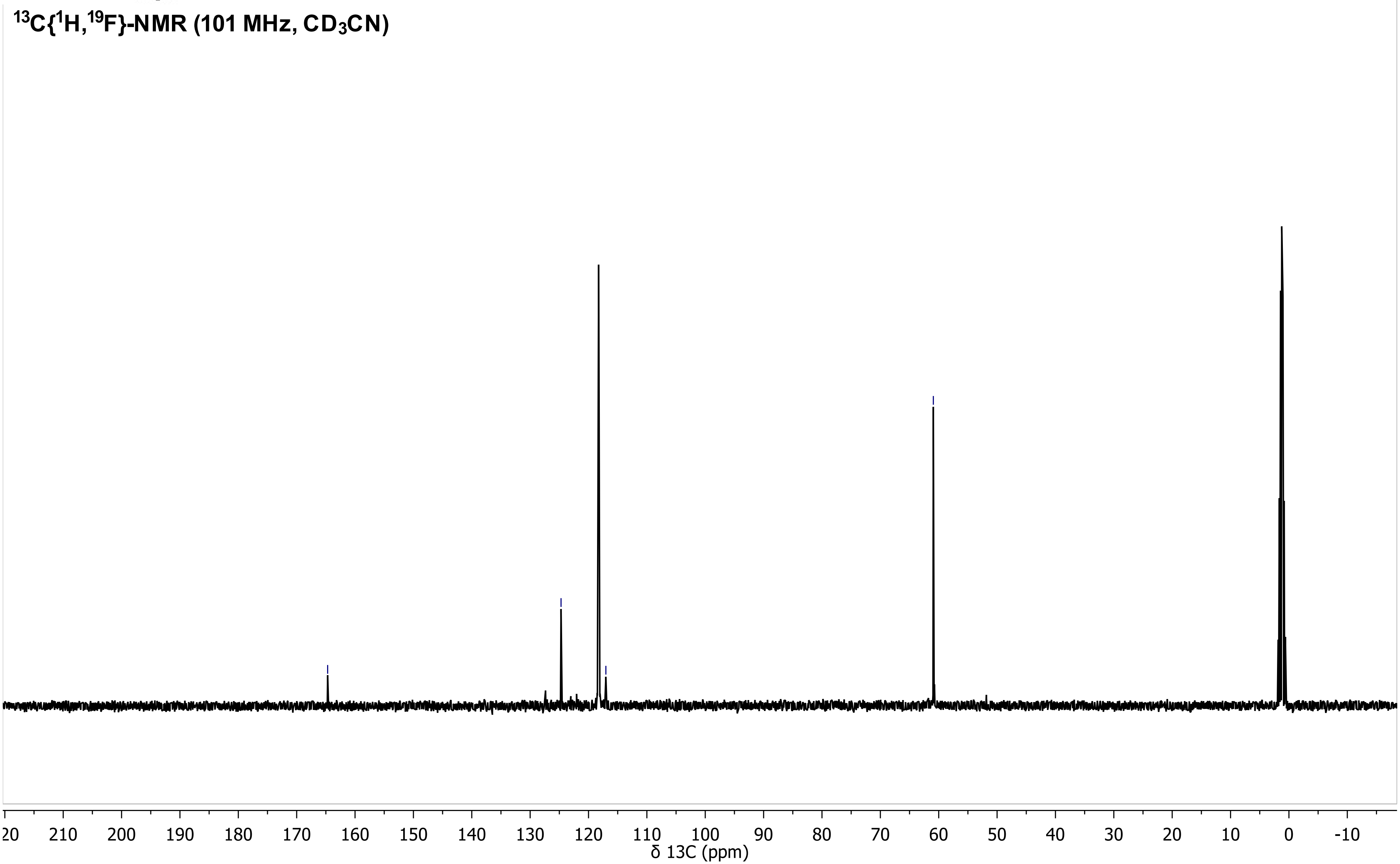

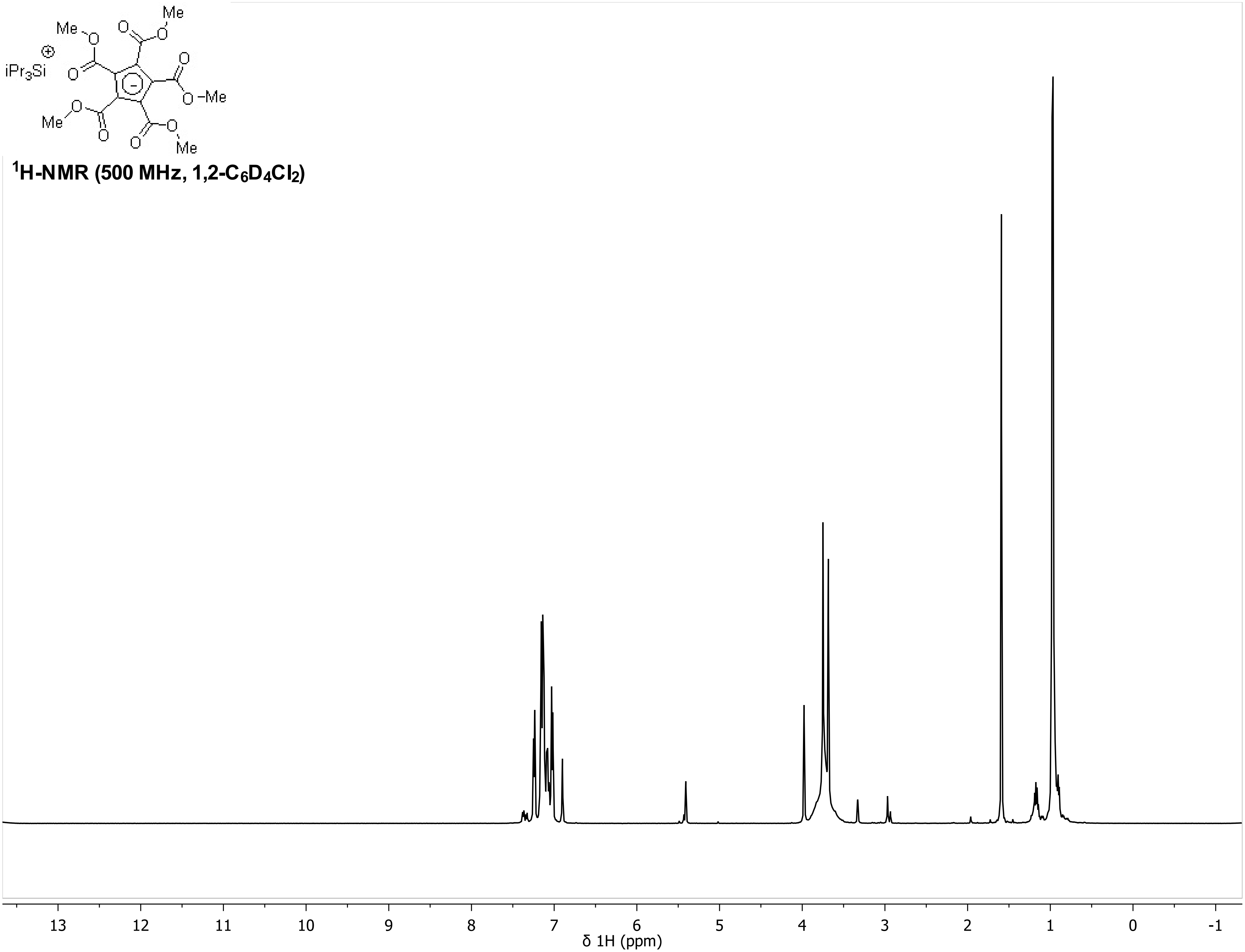

iPr3Si
Me
O
1H-NMR (500 MHz, 1,2-C6D4Cl2)
13
12
11
10
9
8
7
6
5
4
3
2
1
0
-1
δ 1H (ppm)

$^{1}H,^{29}Si$-HMBC (500 MHz, 1,2-$C_6D_4Cl_2$)

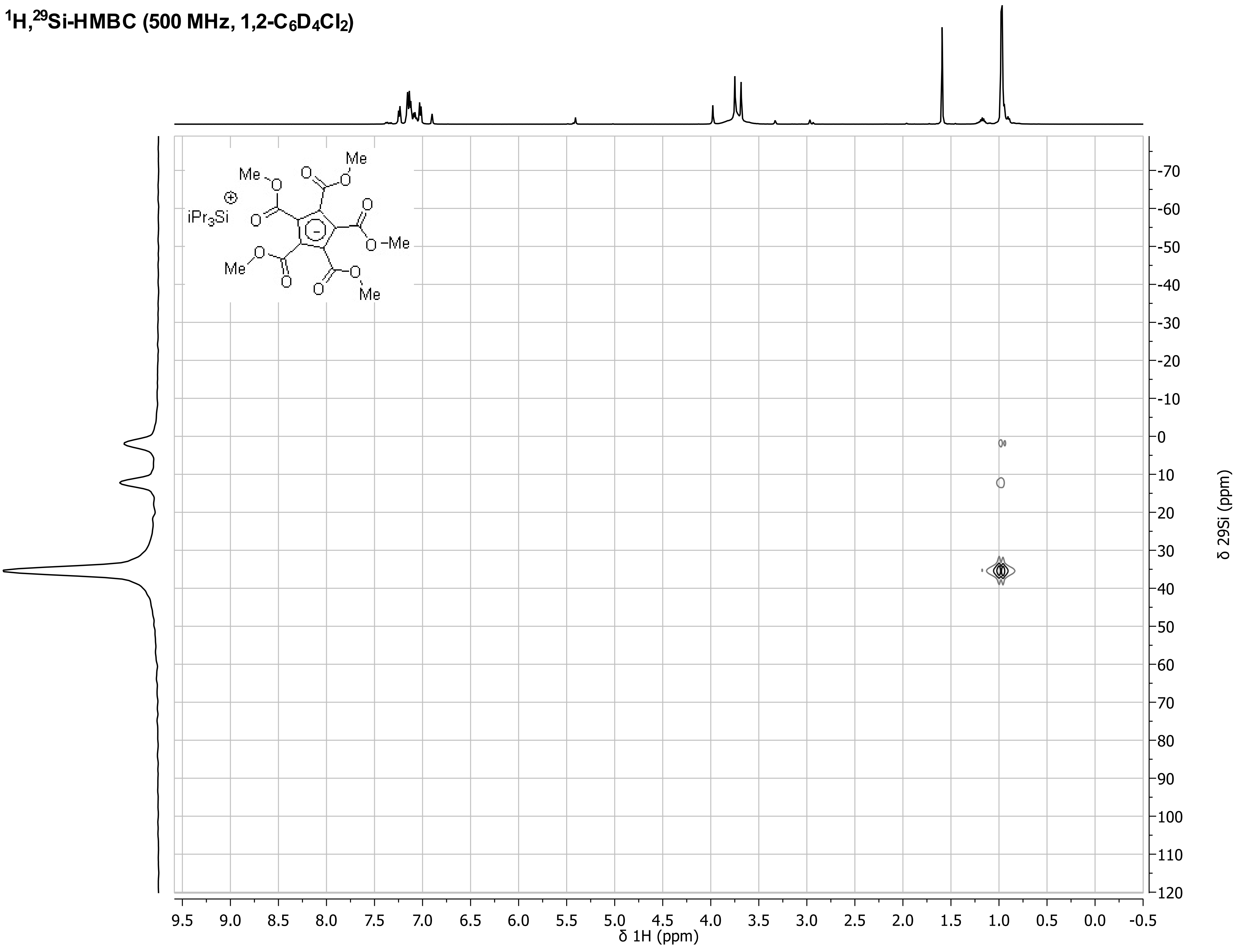

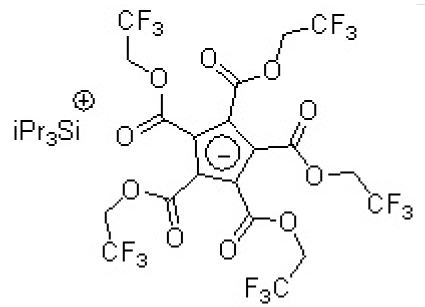

^{1}**H-NMR (500 MHz, 1,2-$C_6D_4Cl_2$)**

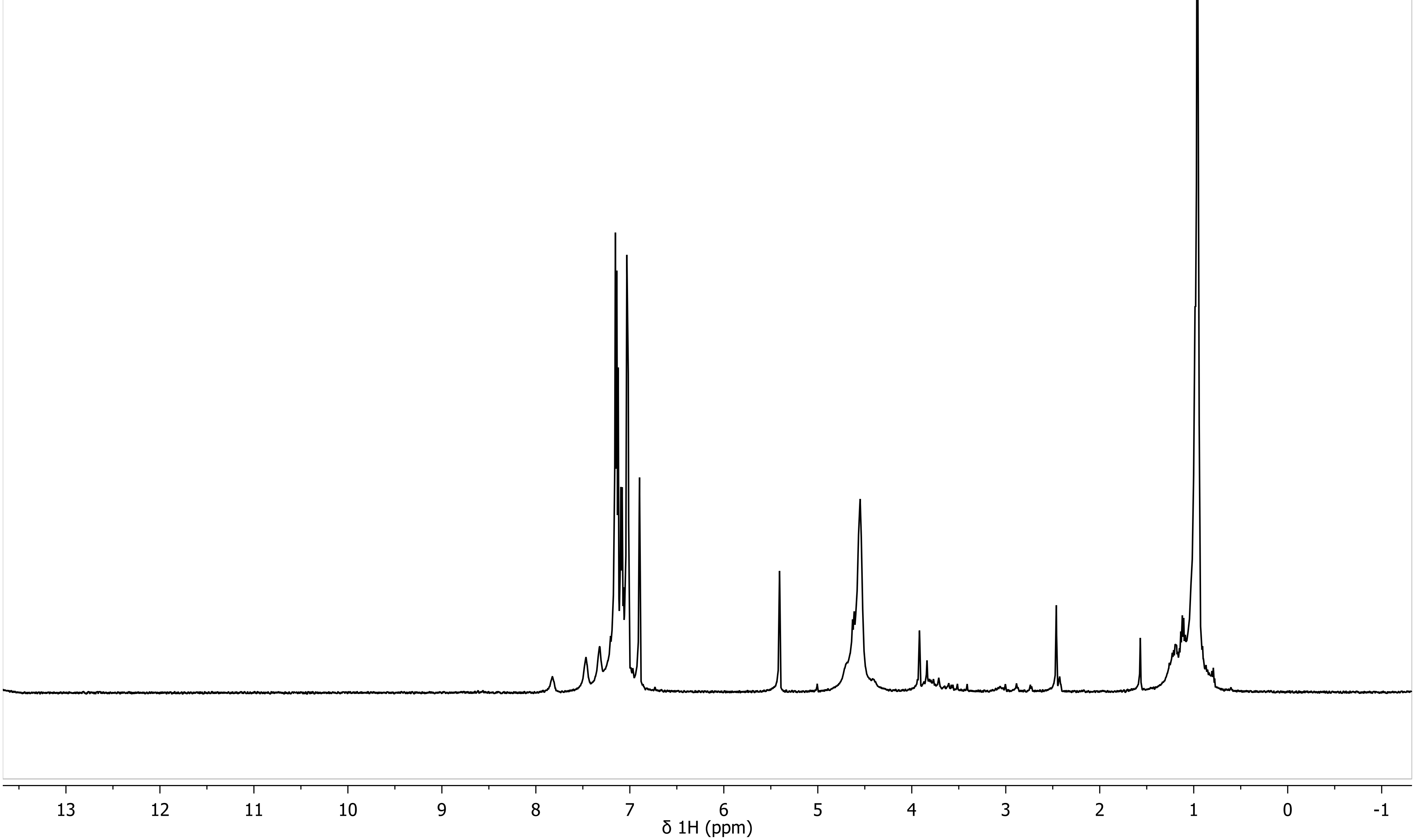

$^{1}H,^{29}Si$-HMBC (500 MHz, 1,2-$C_6D_4Cl_2$)

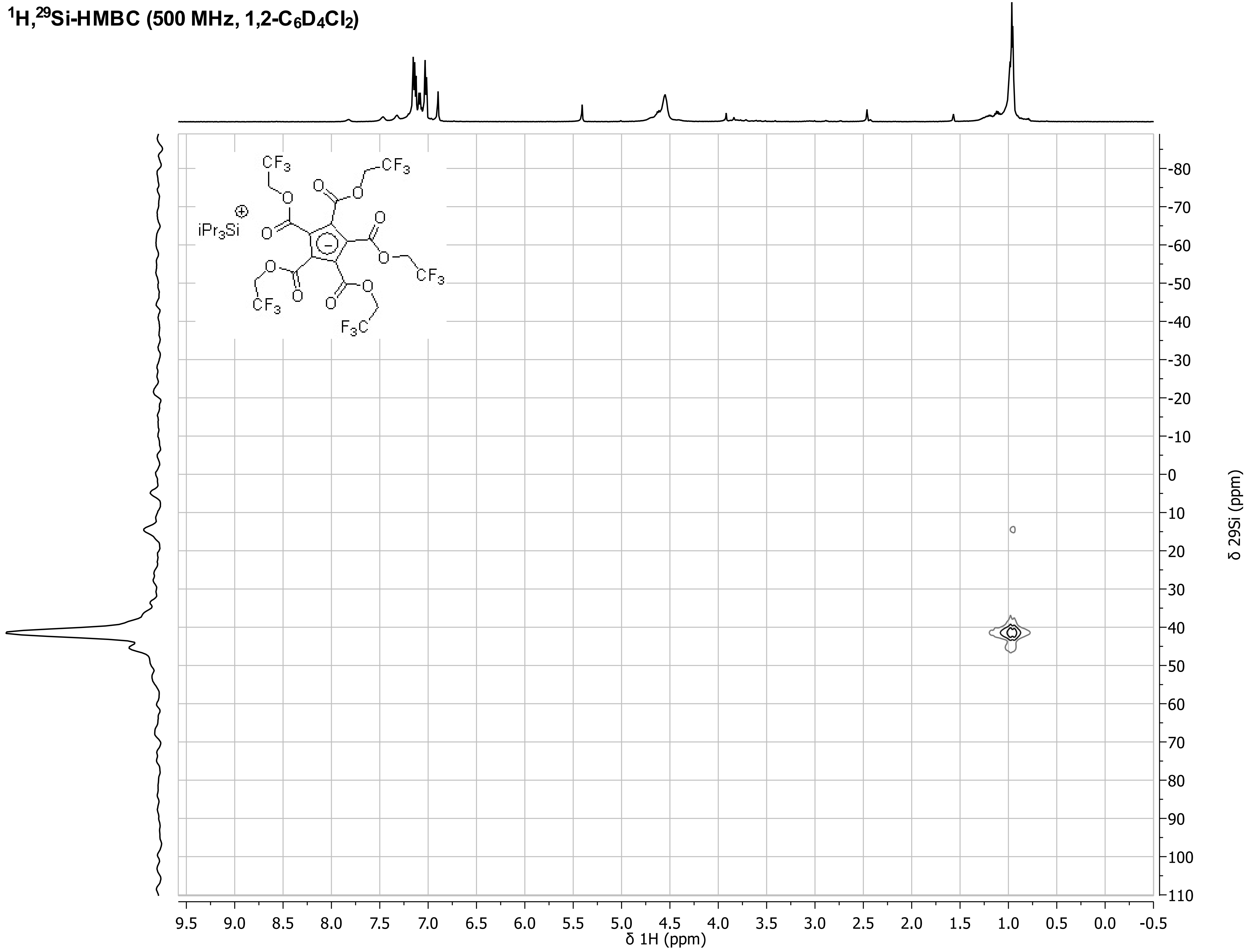

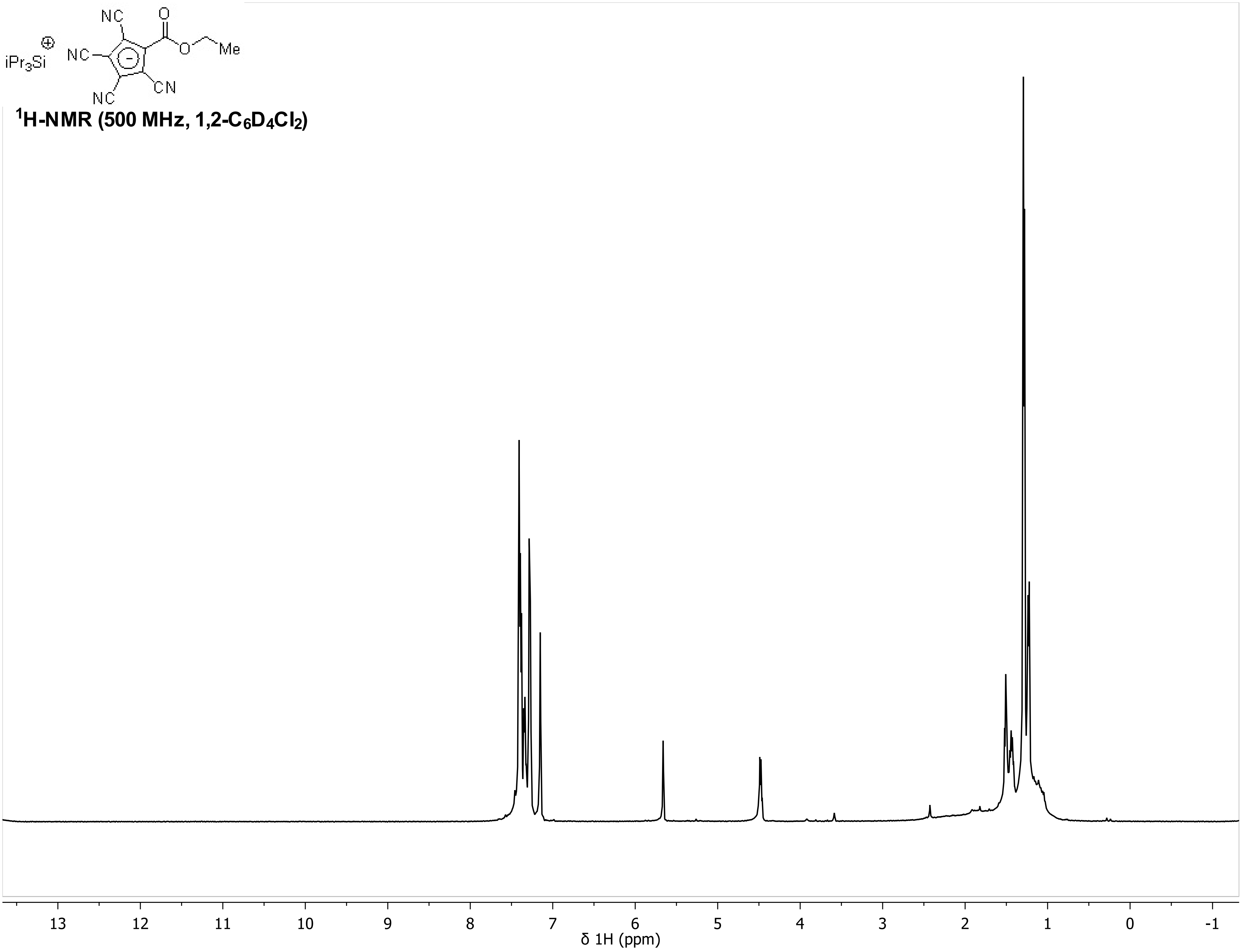
iPr3Si
NC
NC
NC
CN
O
O
Me
1H-NMR (500 MHz, 1,2-C6D4Cl2)
13 12 11 10 9 8 7 6 5 4 3 2 1 0 -1
δ 1H (ppm)

$^{1}H,^{29}Si$-HMBC (500 MHz, 1,2-$C_6D_4Cl_2$)

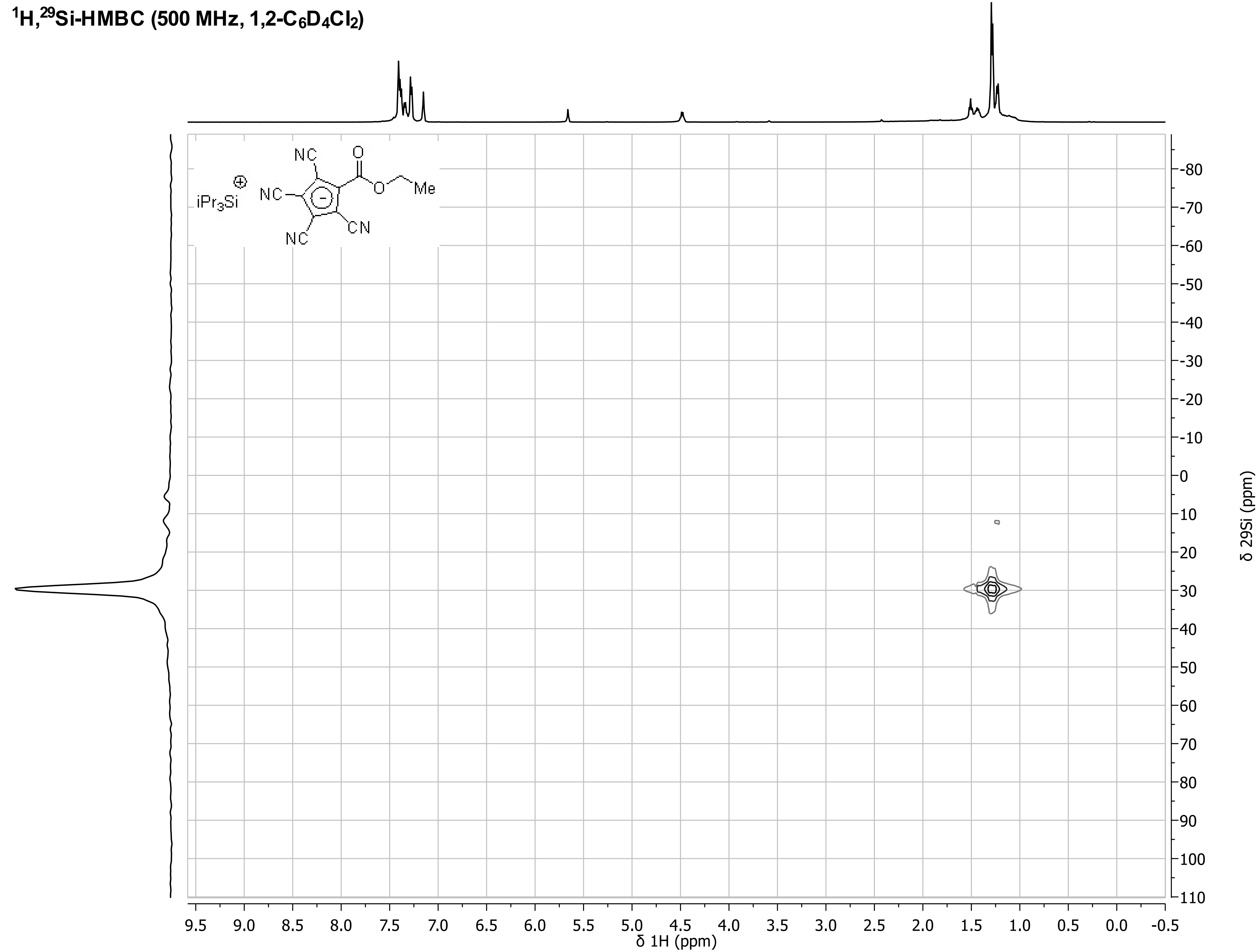

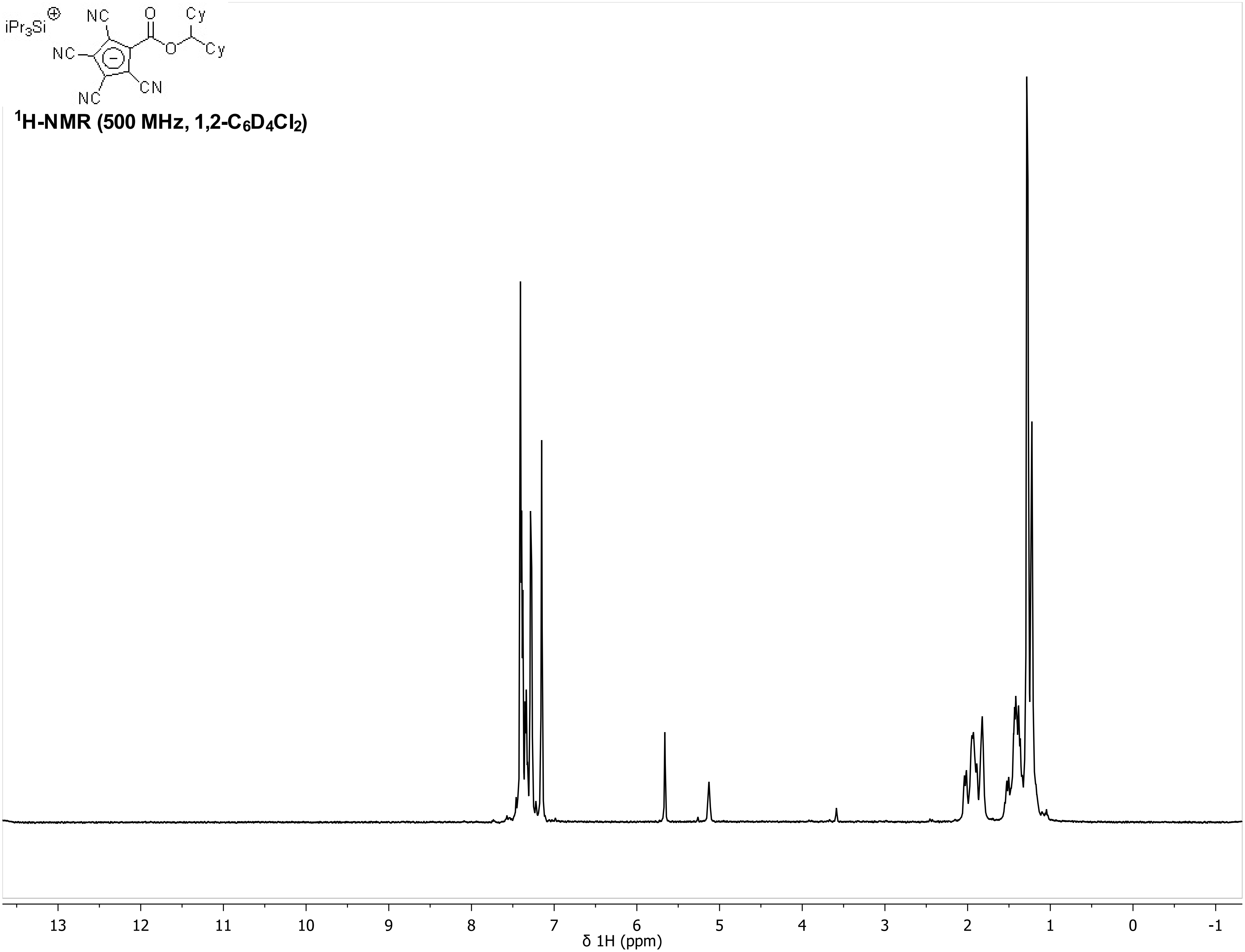

iPr3Si⊕
NC
NC
NC
CN
O
O
Cy
Cy
1H-NMR (500 MHz, 1,2-C6D4Cl2)
13 12 11 10 9 8 7 6 5 4 3 2 1 0 -1
δ 1H (ppm)

$^{1}H,^{29}Si$-HMBC (500 MHz, 1,2-$C_6D_4Cl_2$)

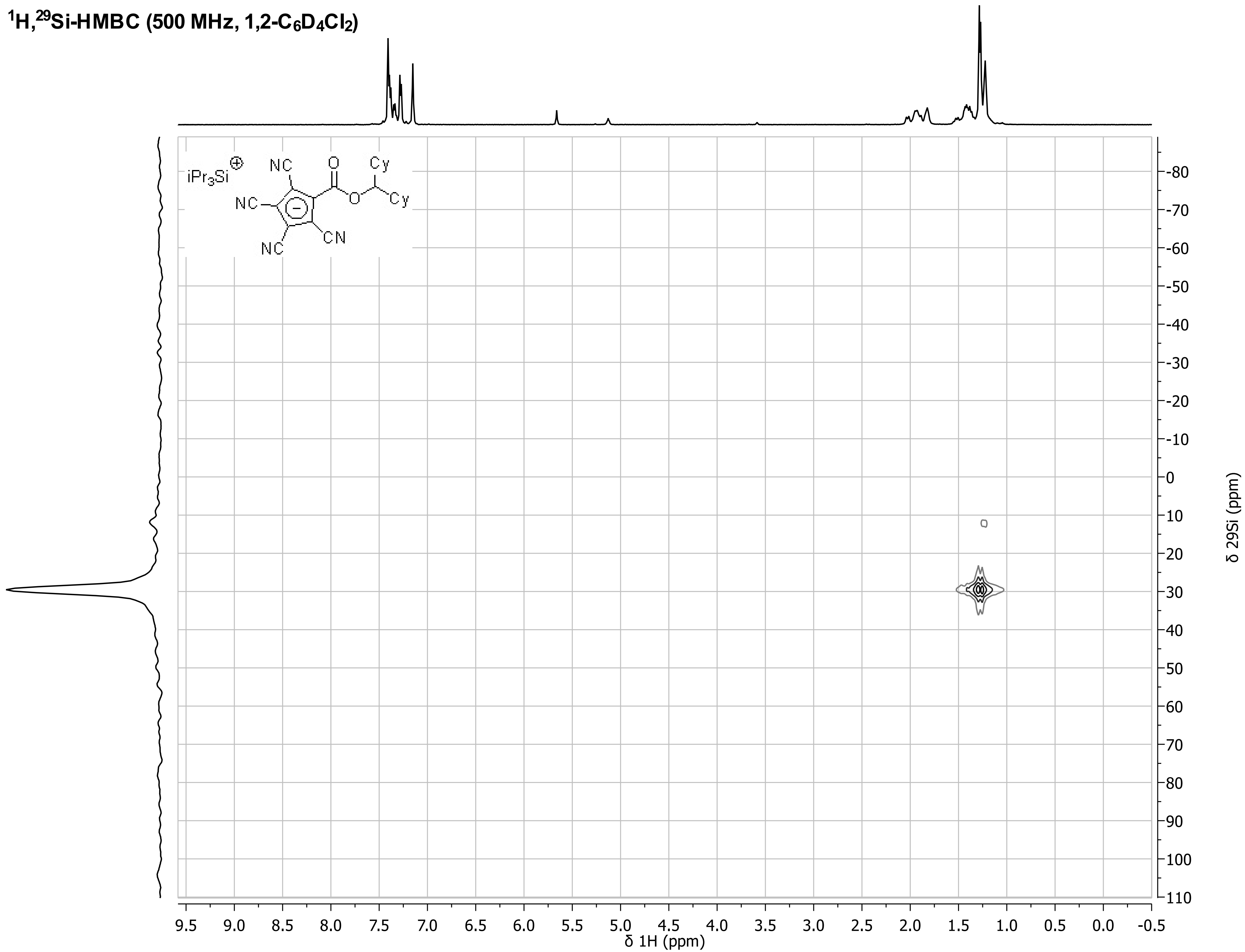

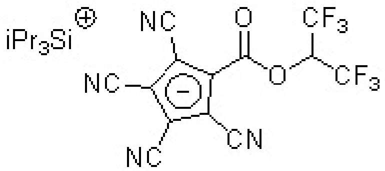
iPr3Si⊕
NC
CF3
NC
O
CF3
NC
CN
O

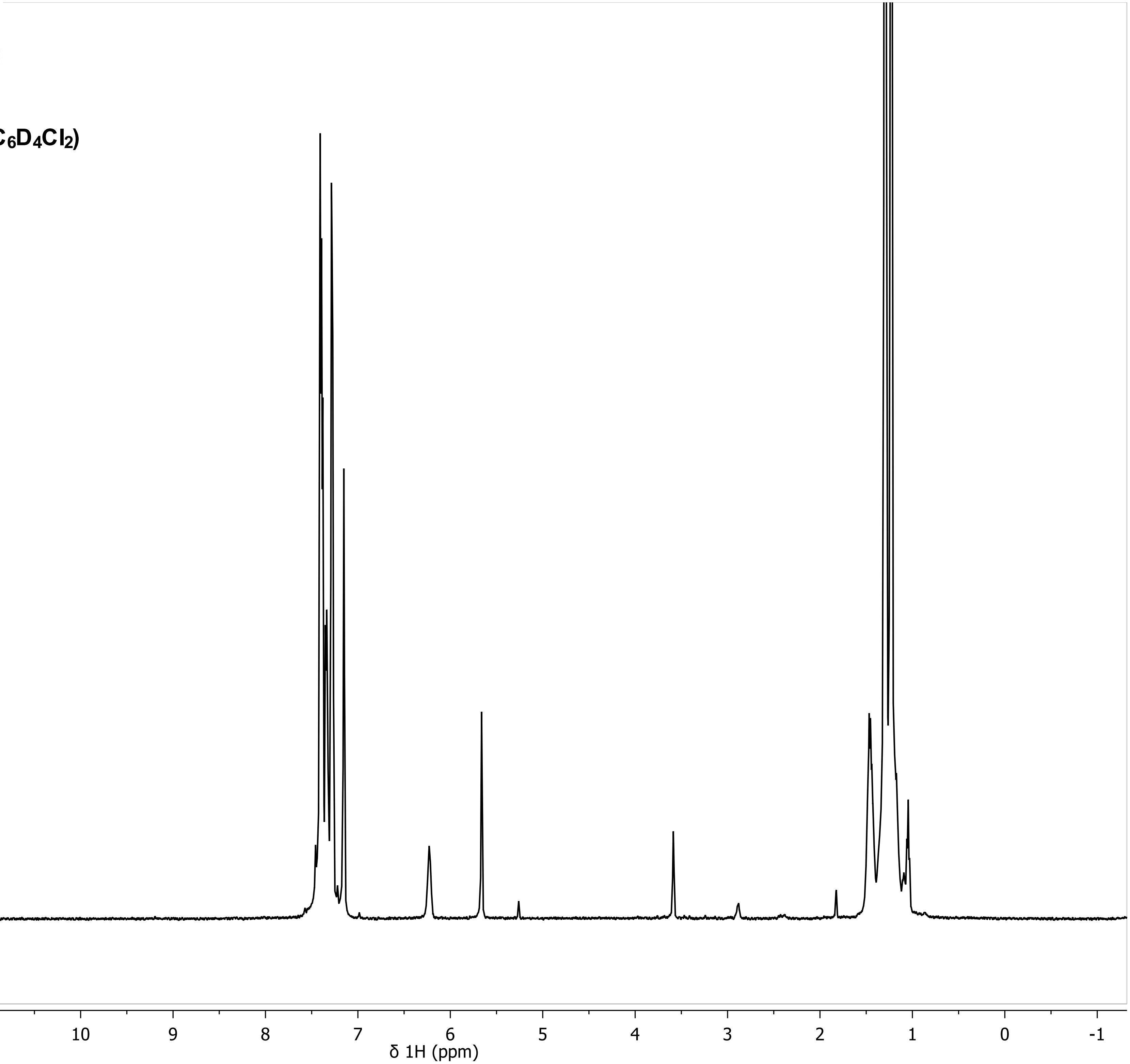
1H-NMR (500 MHz, 1,2-C6D4Cl2)
13
12
11
10
9
8
7
6
5
4
3
2
1
0
-1
δ 1H (ppm)

$^{1}H,^{29}Si$-HMBC (500 MHz, 1,2-$C_6D_4Cl_2$)

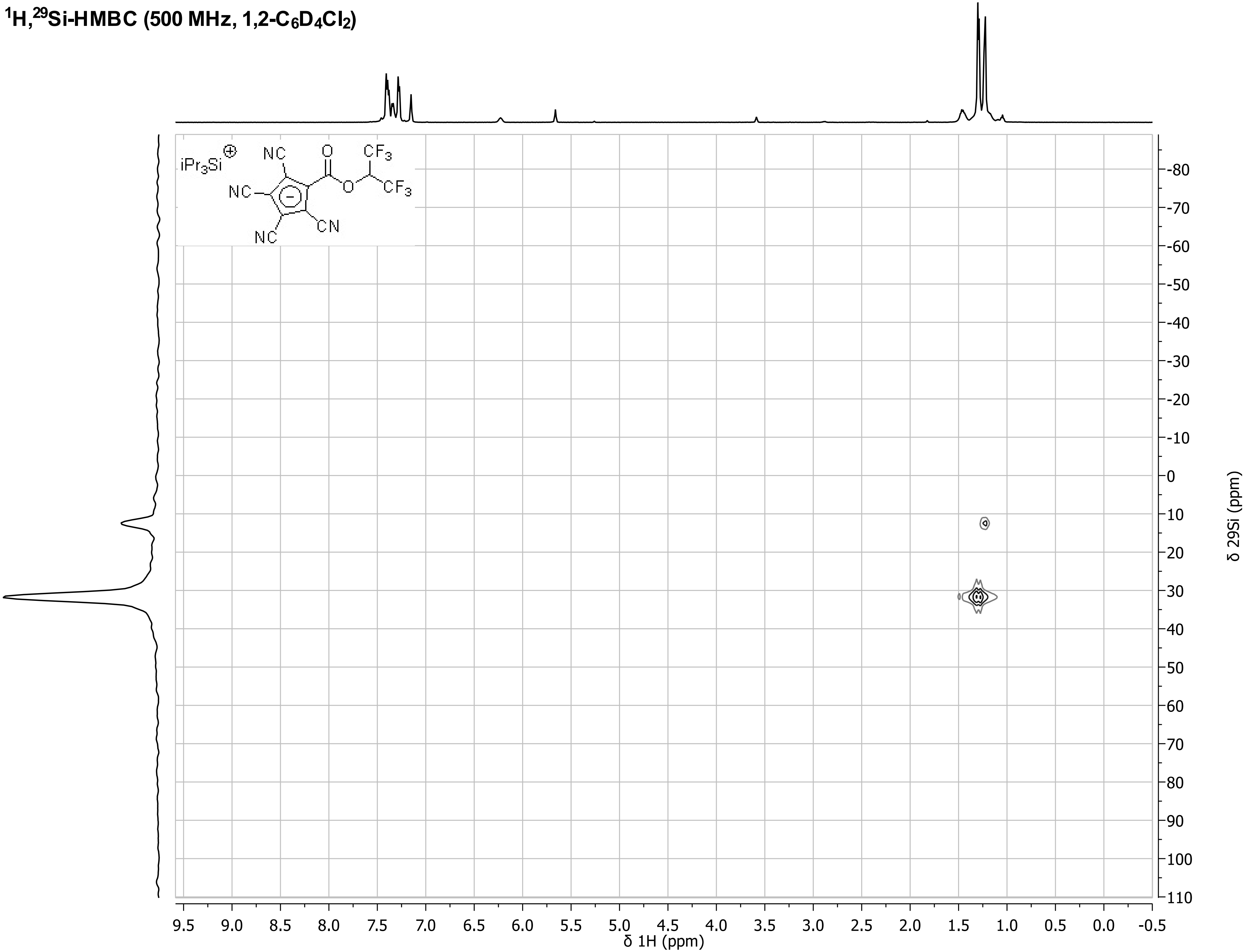

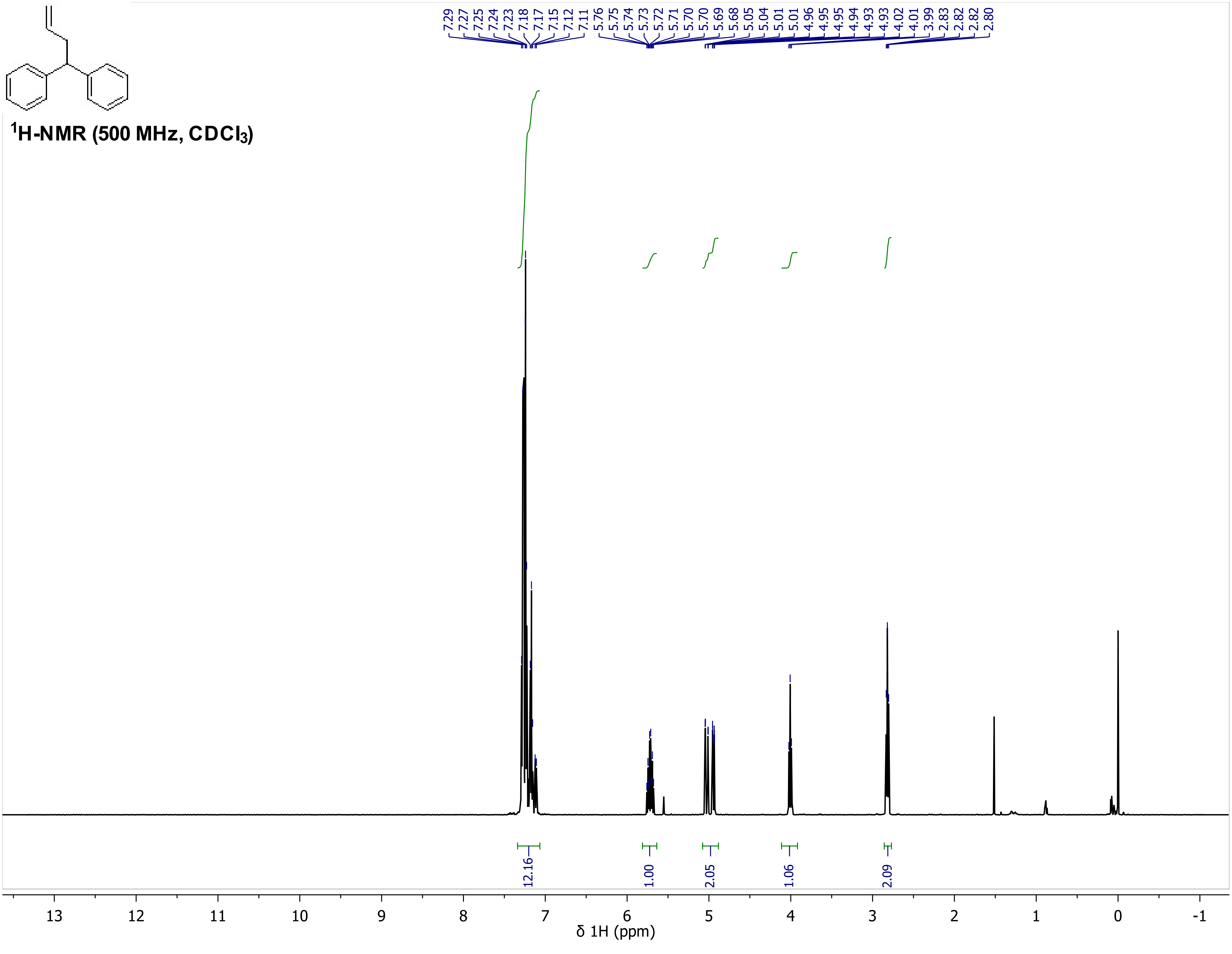

¹H-NMR (500 MHz, CDCl3)
7.29 7.27 7.25 7.24 7.23 7.18 7.17 7.15 7.12 7.11 5.76 5.75 5.74 5.73 5.72 5.71 5.70 5.70 5.69 5.68 5.05 5.04 5.01 5.01 4.96 4.95 4.95 4.94 4.93 4.93 4.02 4.01 3.99 2.83 2.82 2.82 2.80
12.16
1.00
2.05
1.06
2.09
13 12 11 10 9 8 7 6 5 4 3 2 1 0 -1
δ 1H (ppm)

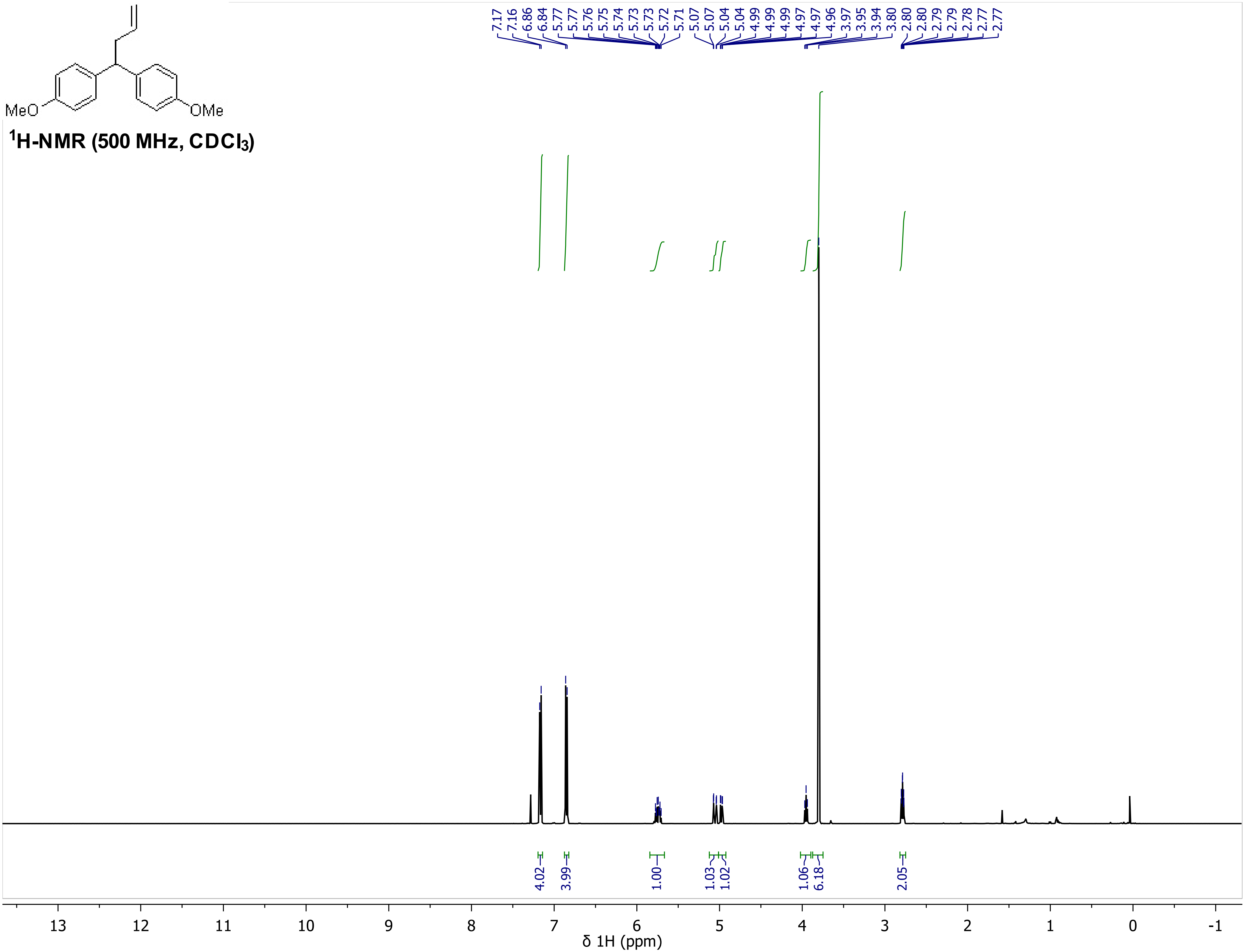

MeO
OMe
^{1}H-NMR (500 MHz, CDCl3)
7.17
7.16
6.86
6.84
5.77
5.77
5.76
5.75
5.74
5.73
5.73
5.72
5.71
5.07
5.07
5.04
5.04
4.99
4.99
4.99
4.97
4.97
4.96
3.97
3.95
3.94
3.80
2.80
2.80
2.79
2.79
2.78
2.77
2.77
4.02
3.99
1.00
1.03
1.02
1.06
6.18
2.05
13
12
11
10
9
8
7
6
5
4
3
2
1
0
-1
δ 1H (ppm)

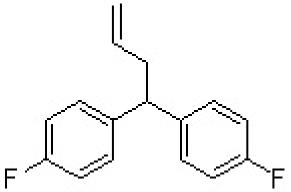

¹H-NMR (500 MHz, $CDCl_3$)

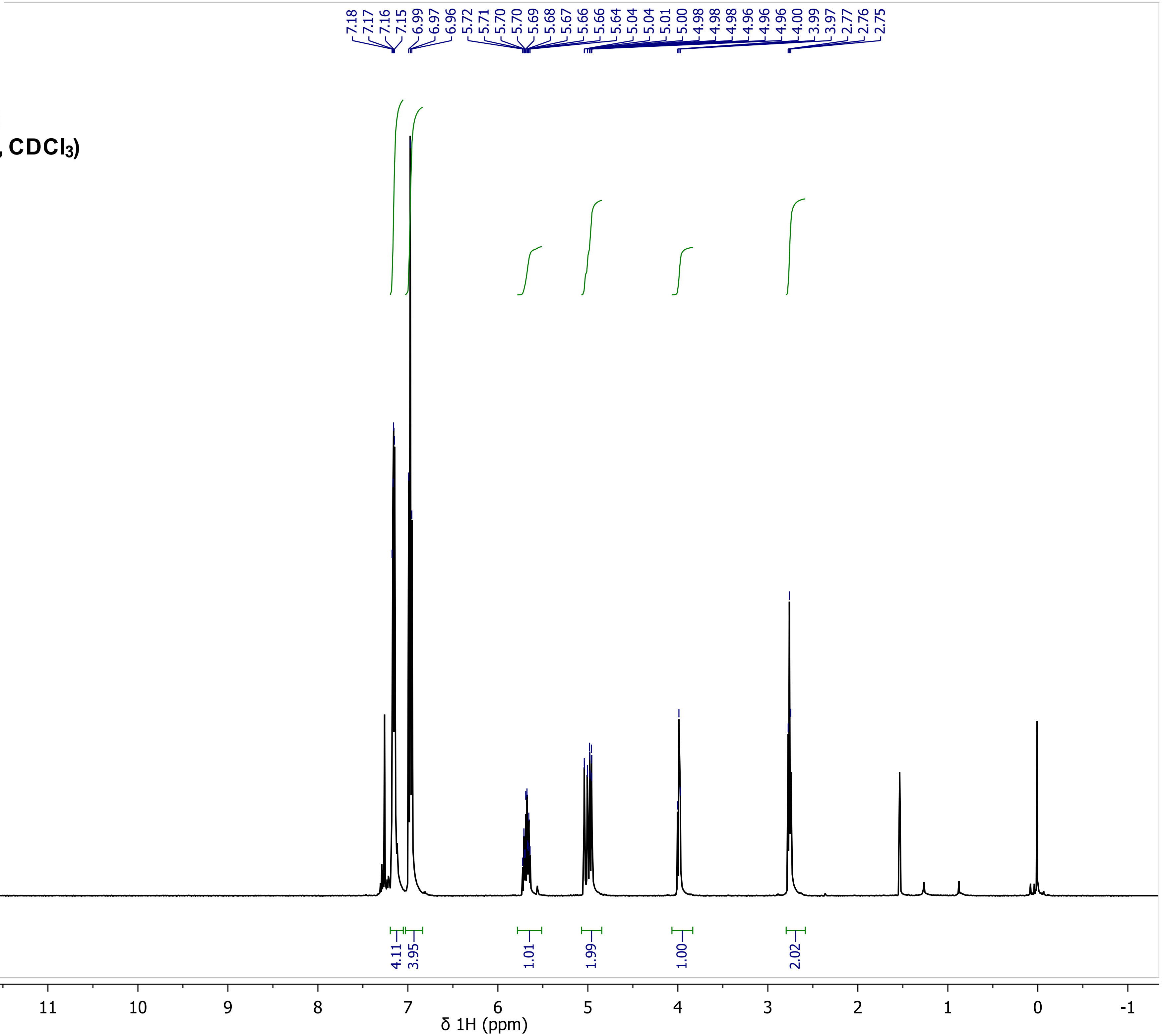

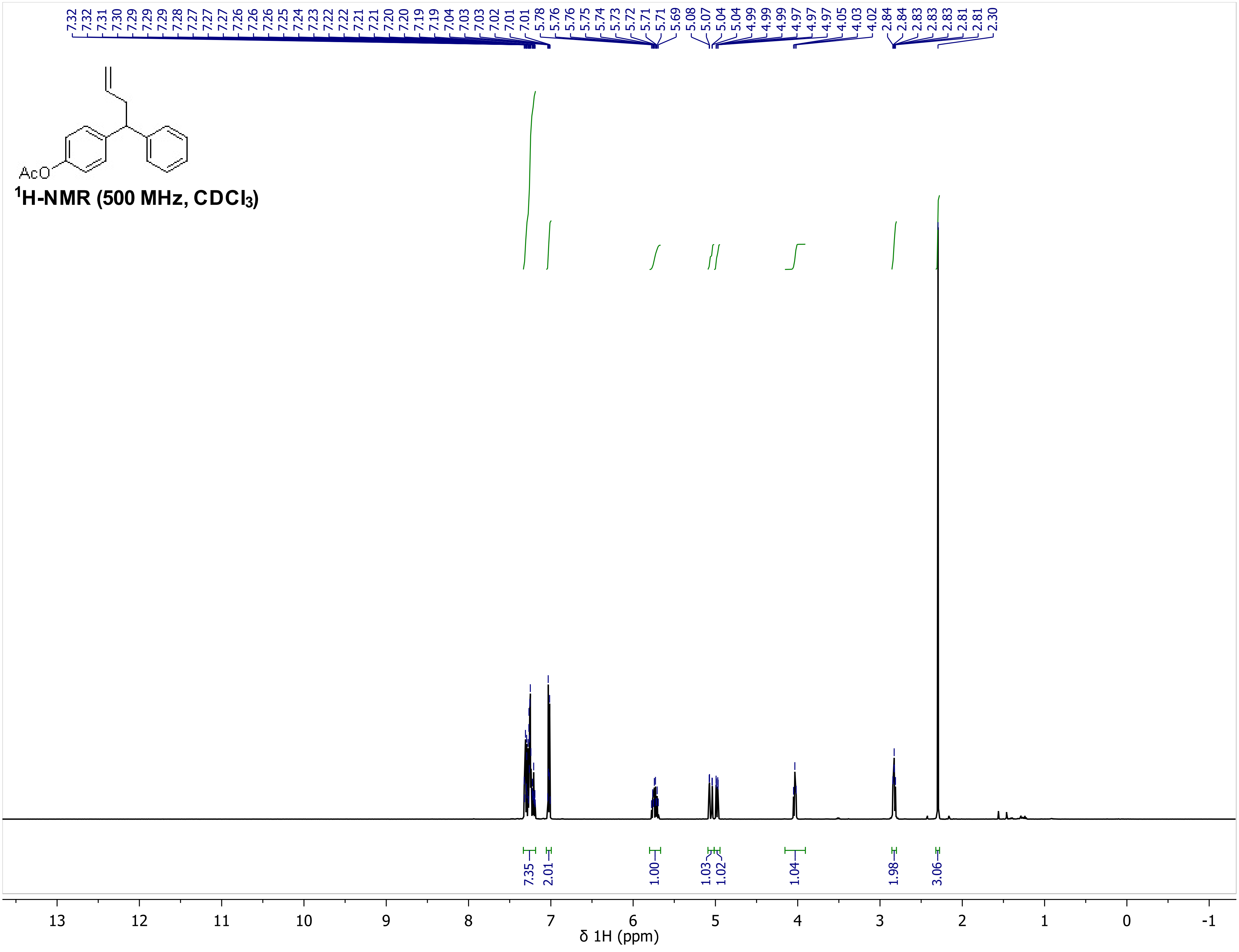
AcO
¹H-NMR (500 MHz, CDCl₃)
7.32 7.32 7.31 7.30 7.29 7.29 7.29 7.28 7.27 7.27 7.27 7.26 7.26 7.26 7.25 7.24 7.23 7.22 7.22 7.21 7.21 7.20 7.20 7.19 7.19 7.04 7.03 7.03 7.02 7.01 7.01 5.78 5.76 5.76 5.75 5.74 5.73 5.72 5.71 5.71 5.69 5.08 5.07 5.04 5.04 4.99 4.99 4.99 4.97 4.97 4.97 4.05 4.03 4.02 2.84 2.84 2.83 2.83 2.83 2.81 2.81 2.30
7.35 2.01 1.00 1.03 1.02 1.04 1.98 3.06
13 12 11 10 9 8 7 6 5 4 3 2 1 0 -1
δ 1H (ppm)

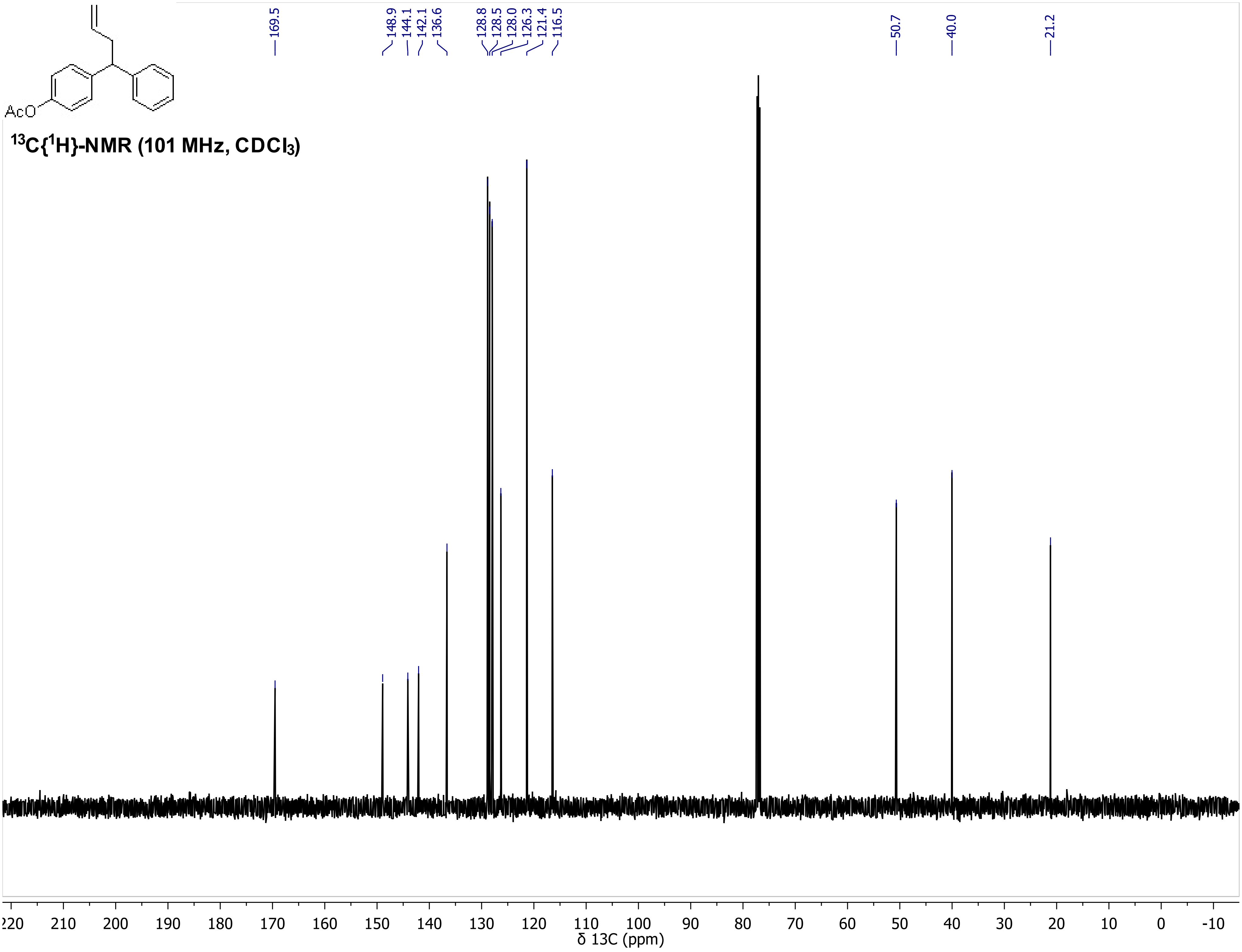
AcO
$^{13}C\{^{1}H\}$-NMR (101 MHz, $CDCl_3$)
169.5
148.9
144.1
142.1
136.6
128.8
128.5
128.0
126.3
121.4
116.5
50.7
40.0
21.2
220 210 200 190 180 170 160 150 140 130 120 110 100 90 80 70 60 50 40 30 20 10 0 -10
δ 13C (ppm)

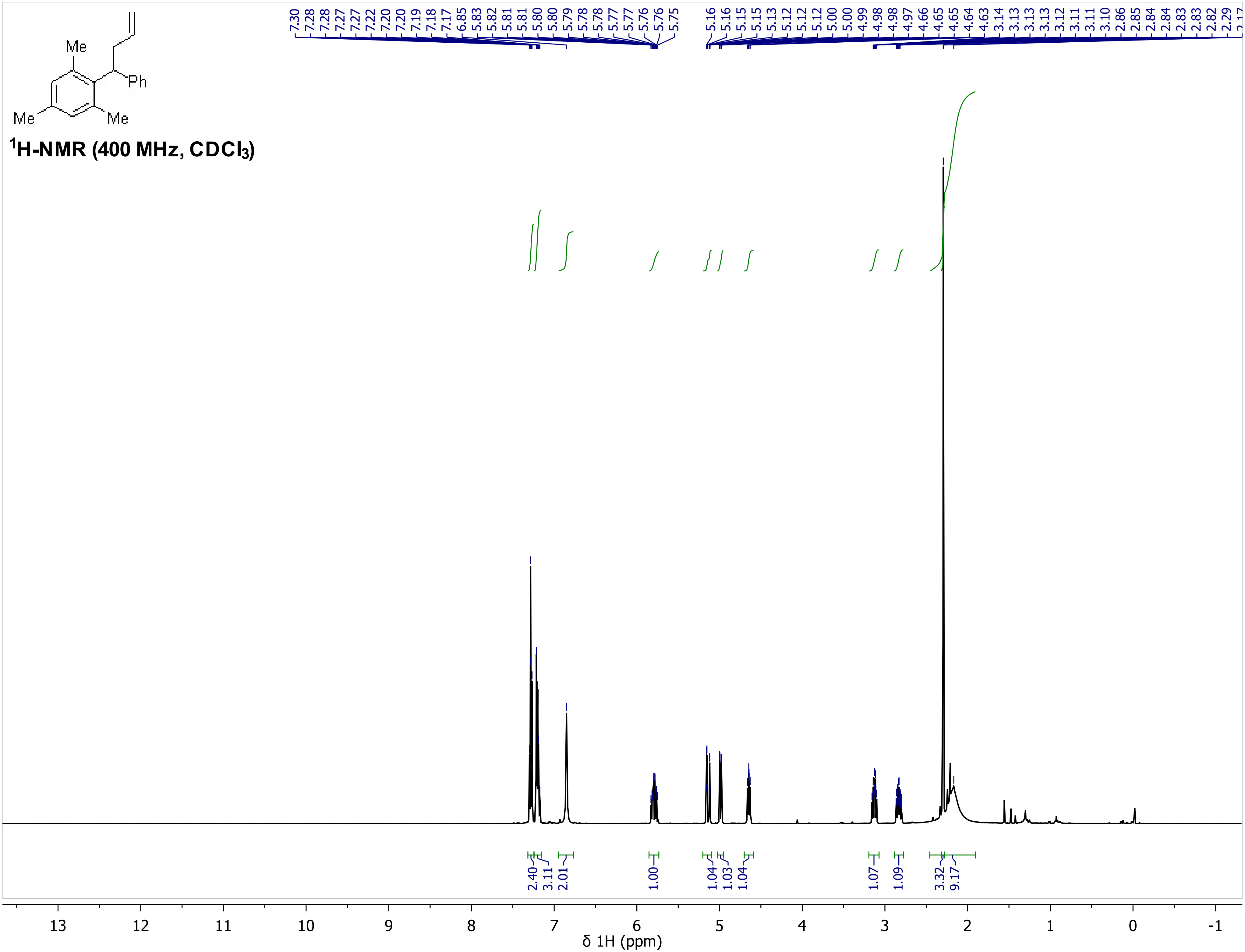

Me
Ph
Me
Me
¹H-NMR (400 MHz, CDCl₃)
δ 1H (ppm)

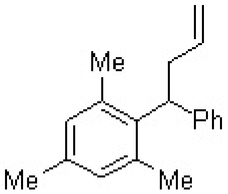

$^{13}C\{^{1}H\}$-NMR (101 MHz, $CDCl_3$)

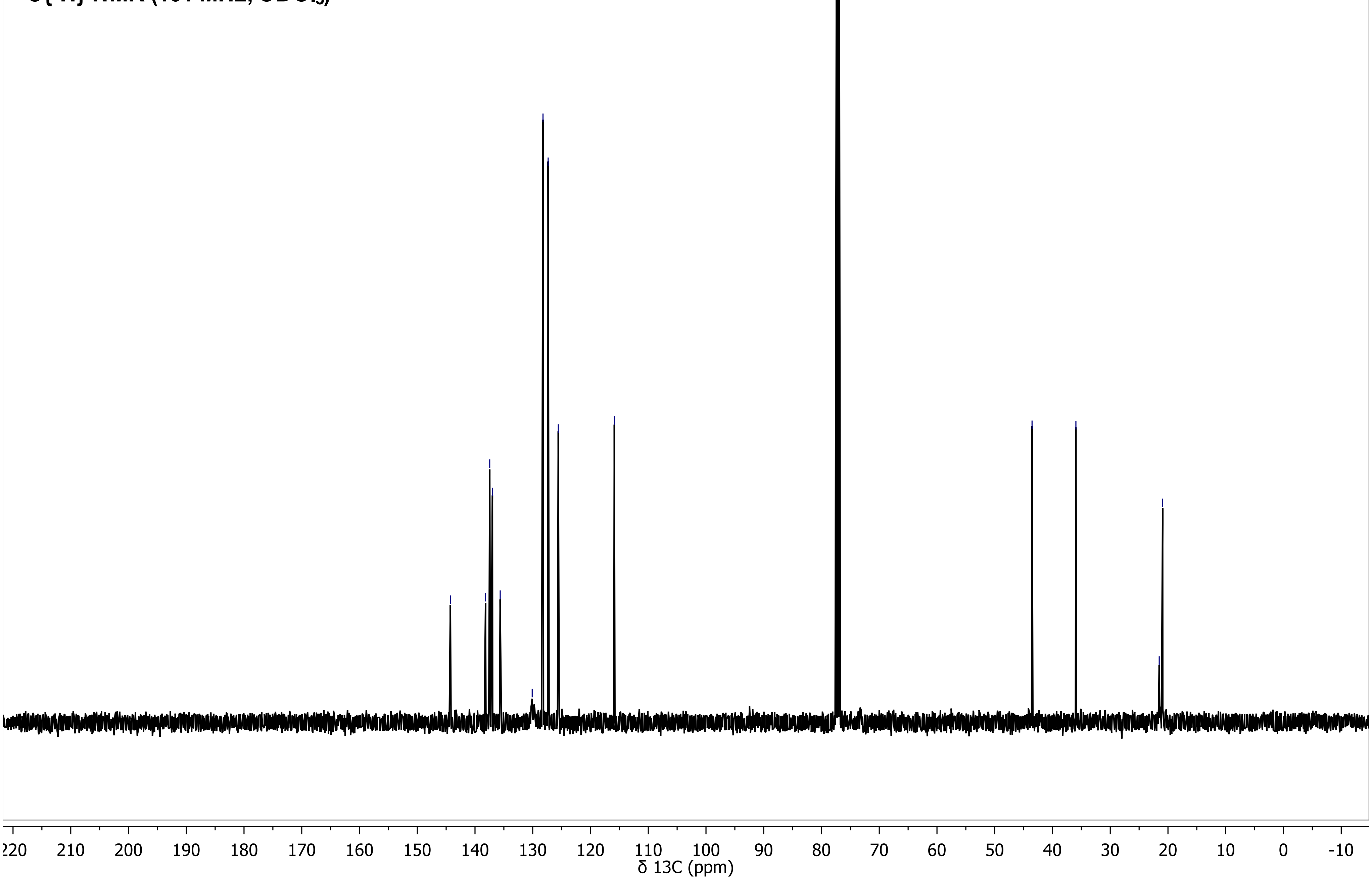

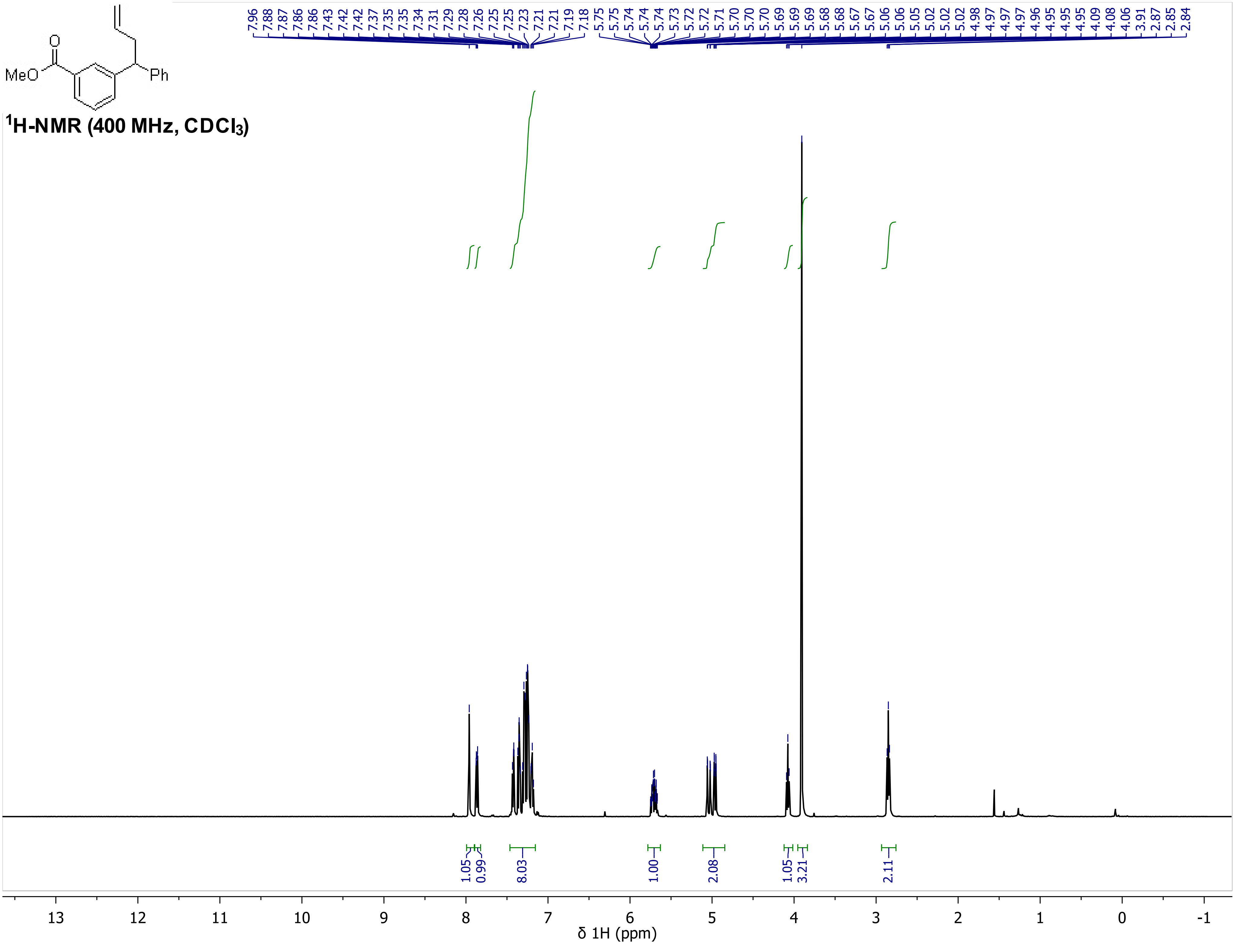
MeO
O
Ph
1H-NMR (400 MHz, CDCl3)
7.96 7.88 7.87 7.86 7.86 7.43 7.42 7.42 7.37 7.35 7.35 7.34 7.31 7.29 7.28 7.26 7.25 7.25 7.23 7.21 7.21 7.19 7.18 5.75 5.75 5.74 5.74 5.74 5.73 5.72 5.72 5.71 5.70 5.70 5.70 5.69 5.69 5.69 5.68 5.68 5.67 5.67 5.06 5.06 5.05 5.02 5.02 5.02 4.98 4.97 4.97 4.97 4.96 4.95 4.95 4.95 4.09 4.08 4.06 3.91 2.87 2.85 2.84
1.05
0.99
8.03
1.00
2.08
1.05
3.21
2.11
13 12 11 10 9 8 7 6 5 4 3 2 1 0 -1
δ 1H (ppm)

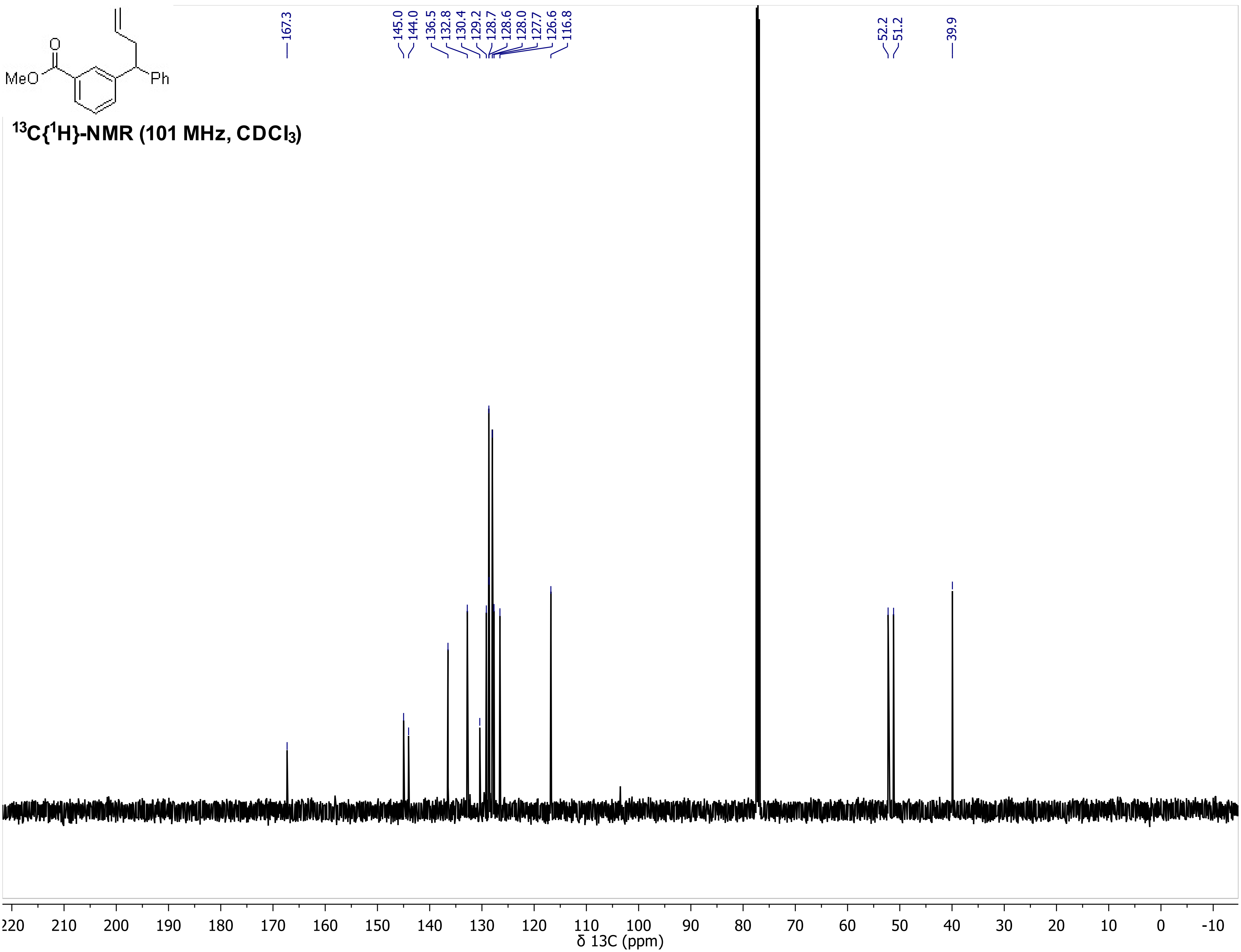

MeO
Ph
O
^{13}C{^{1}H}-NMR (101 MHz, CDCl$_3$)
167.3
145.0
144.0
136.5
132.8
130.4
129.2
128.7
128.6
128.0
127.7
126.6
116.8
52.2
51.2
39.9
δ 13C (ppm)

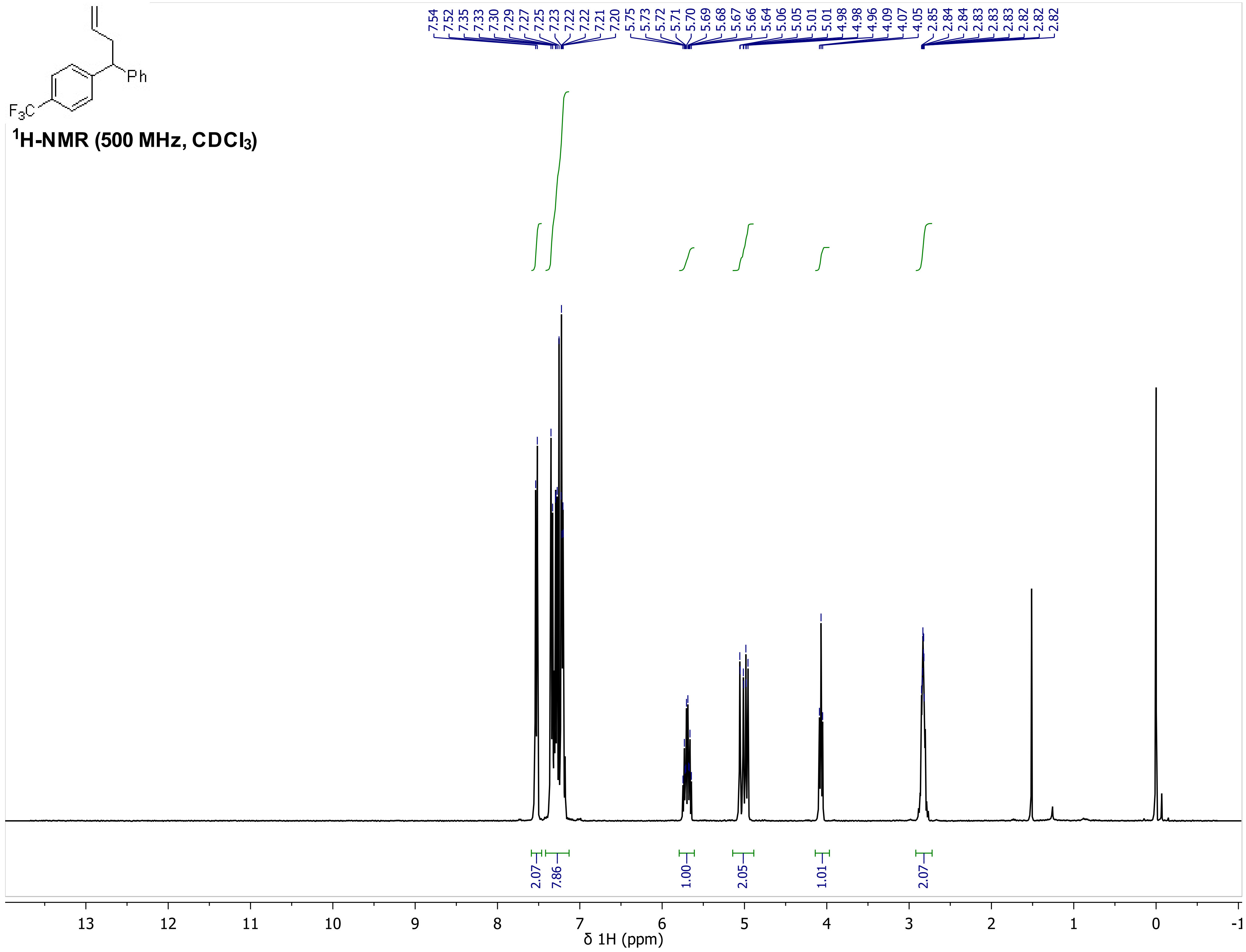

Ph
F_3C
^{1}H-NMR (500 MHz, $CDCl_3$)
7.54 7.52 7.35 7.33 7.30 7.29 7.27 7.25 7.23 7.22 7.22 7.21 7.20 5.75 5.73 5.72 5.71 5.70 5.69 5.68 5.67 5.66 5.64 5.06 5.05 5.01 5.01 4.98 4.98 4.96 4.09 4.07 4.05 2.85 2.84 2.84 2.83 2.83 2.83 2.82 2.82 2.82
2.07 7.86 1.00 2.05 1.01 2.07
13 12 11 10 9 8 7 6 5 4 3 2 1 0 -1
δ 1H (ppm)

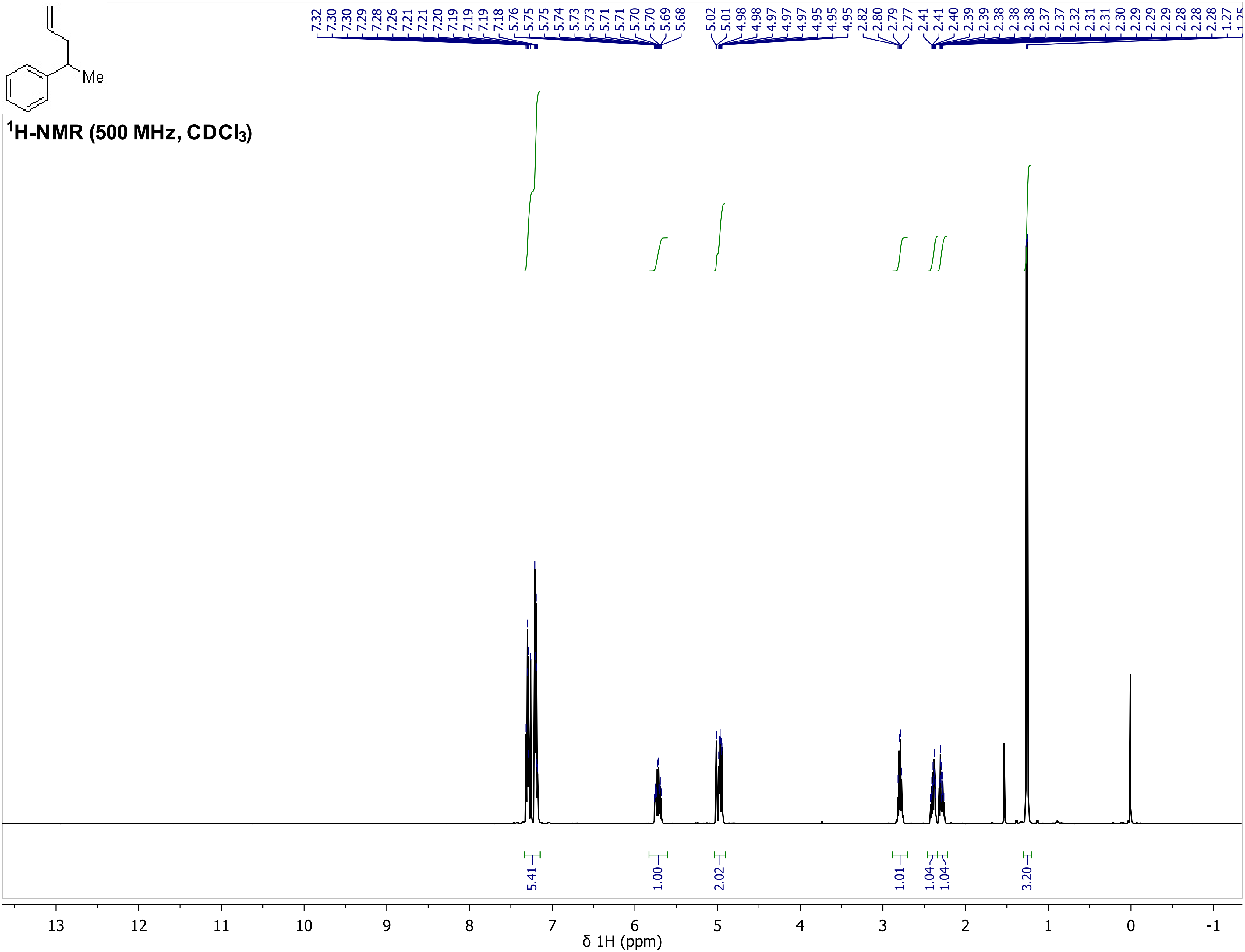
Me
1H-NMR (500 MHz, CDCl3)
7.32 7.30 7.30 7.29 7.28 7.26 7.21 7.21 7.20 7.19 7.19 7.19 7.18 5.76 5.75 5.75 5.74 5.73 5.73 5.71 5.71 5.70 5.70 5.69 5.68
5.02 5.01 4.98 4.98 4.97 4.97 4.97 4.95 4.95 4.95 2.82 2.80 2.79 2.77 2.41 2.41 2.40 2.39 2.39 2.38 2.38 2.38 2.38 2.37 2.37 2.32 2.31 2.31 2.30 2.29 2.29 2.29 2.28 2.28 2.28 1.27
5.41
1.00
2.02
1.01
1.04
1.04
3.20
13 12 11 10 9 8 7 6 5 4 3 2 1 0 -1
δ 1H (ppm)

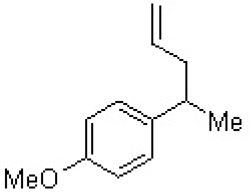

^{1}H-NMR (500 MHz, CDCl$_3$)

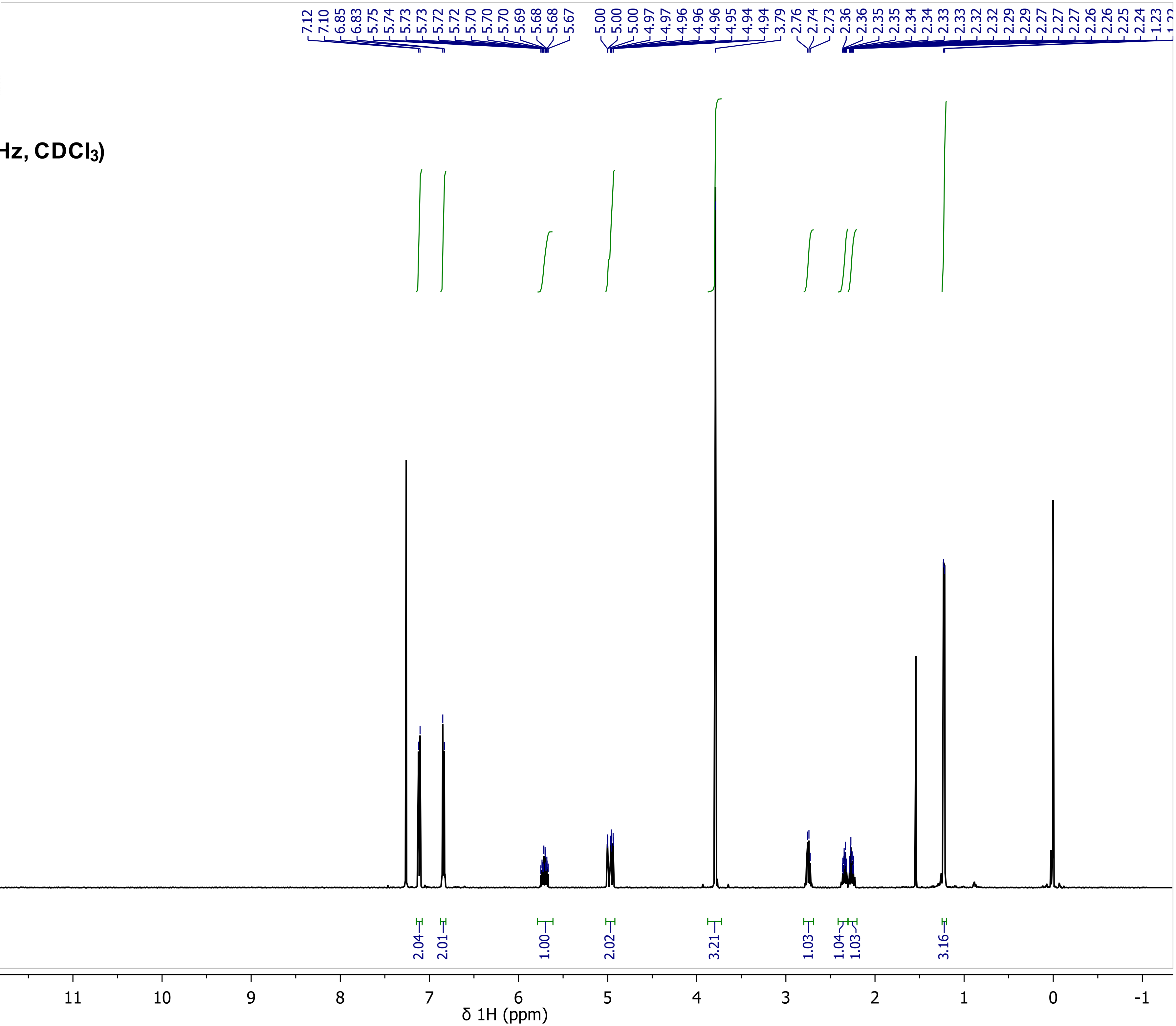

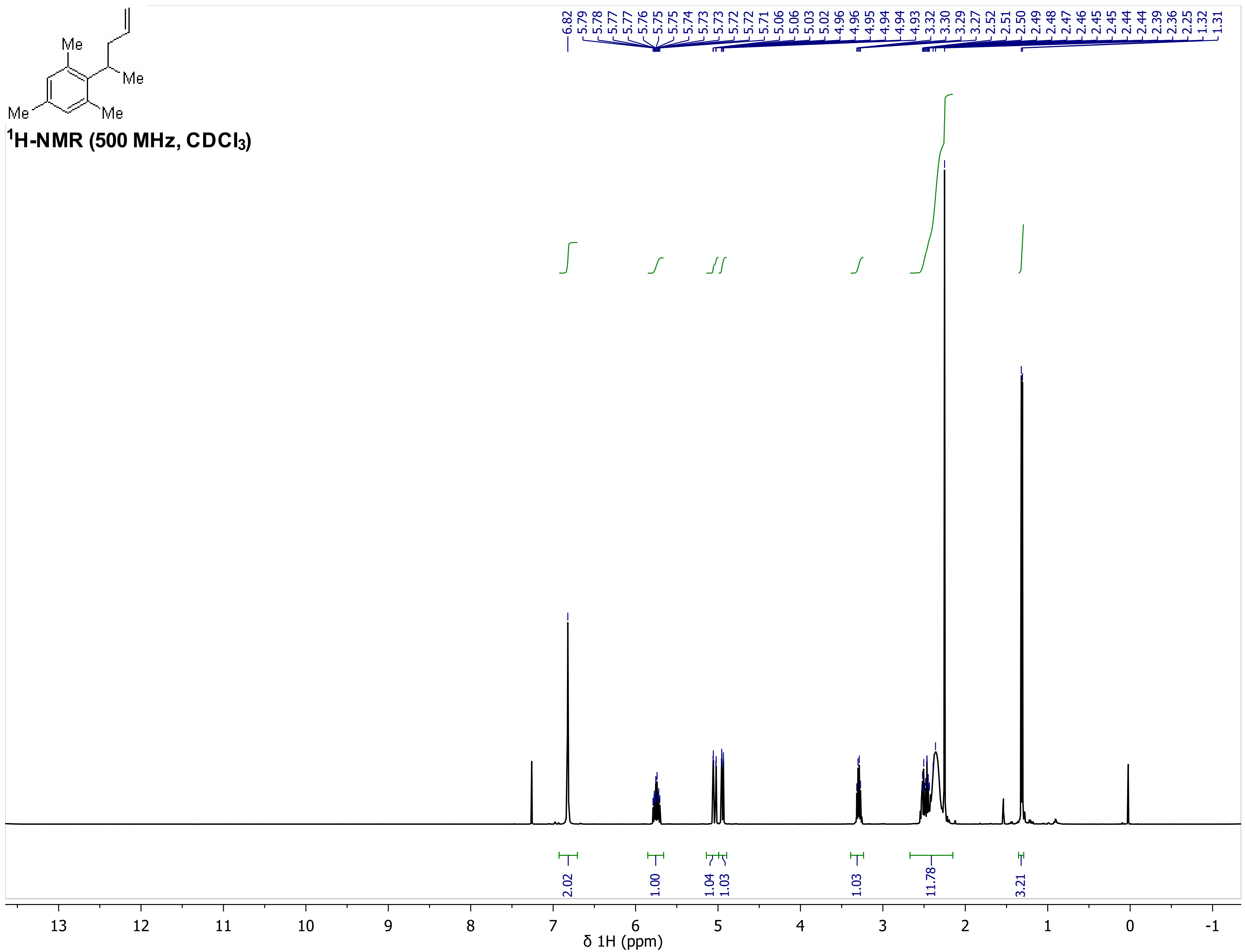
Me
Me
Me
Me
1H-NMR (500 MHz, CDCl3)
6.82
5.79
5.78
5.77
5.77
5.76
5.75
5.75
5.74
5.73
5.73
5.72
5.72
5.71
5.06
5.06
5.03
5.02
4.96
4.96
4.95
4.94
4.94
4.93
3.32
3.30
3.29
3.27
2.52
2.51
2.50
2.49
2.48
2.47
2.46
2.45
2.45
2.44
2.44
2.39
2.36
2.25
1.32
1.31
2.02
1.00
1.04
1.03
1.03
11.78
3.21
13
12
11
10
9
8
7
6
5
4
3
2
1
0
-1
δ 1H (ppm)

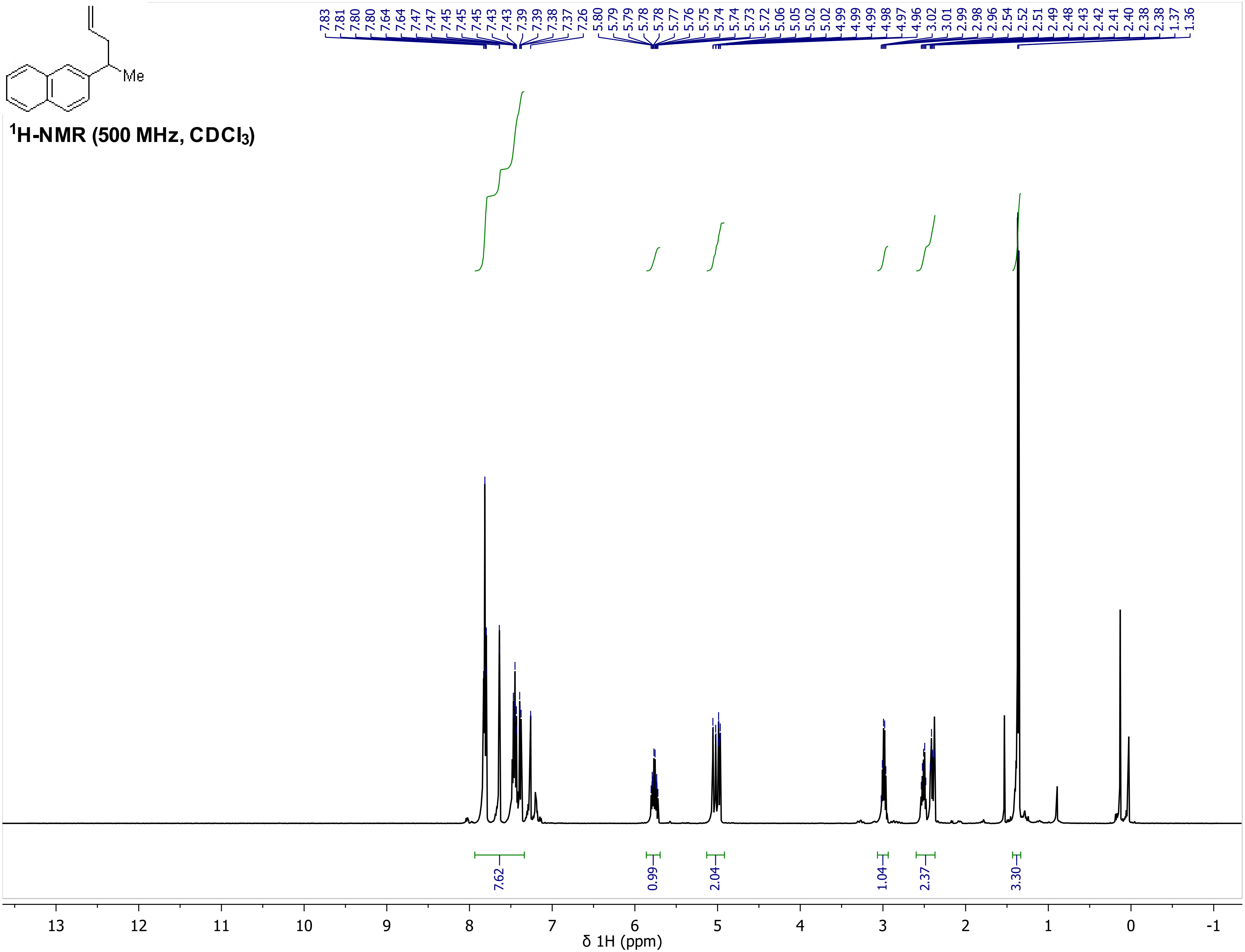

Me
¹H-NMR (500 MHz, CDCl3)
7.83 7.81 7.80 7.80 7.64 7.64 7.47 7.47 7.45 7.45 7.45 7.43 7.43 7.39 7.39 7.38 7.37 7.26 5.80 5.79 5.79 5.78 5.78 5.77 5.76 5.75 5.74 5.74 5.73 5.72 5.06 5.05 5.02 5.02 4.99 4.99 4.99 4.98 4.97 4.96 3.02 3.01 2.99 2.98 2.96 2.54 2.52 2.51 2.49 2.48 2.43 2.42 2.41 2.40 2.38 2.38 1.37 1.36
7.62
0.99
2.04
1.04
2.37
3.30
13 12 11 10 9 8 7 6 5 4 3 2 1 0 -1
δ 1H (ppm)

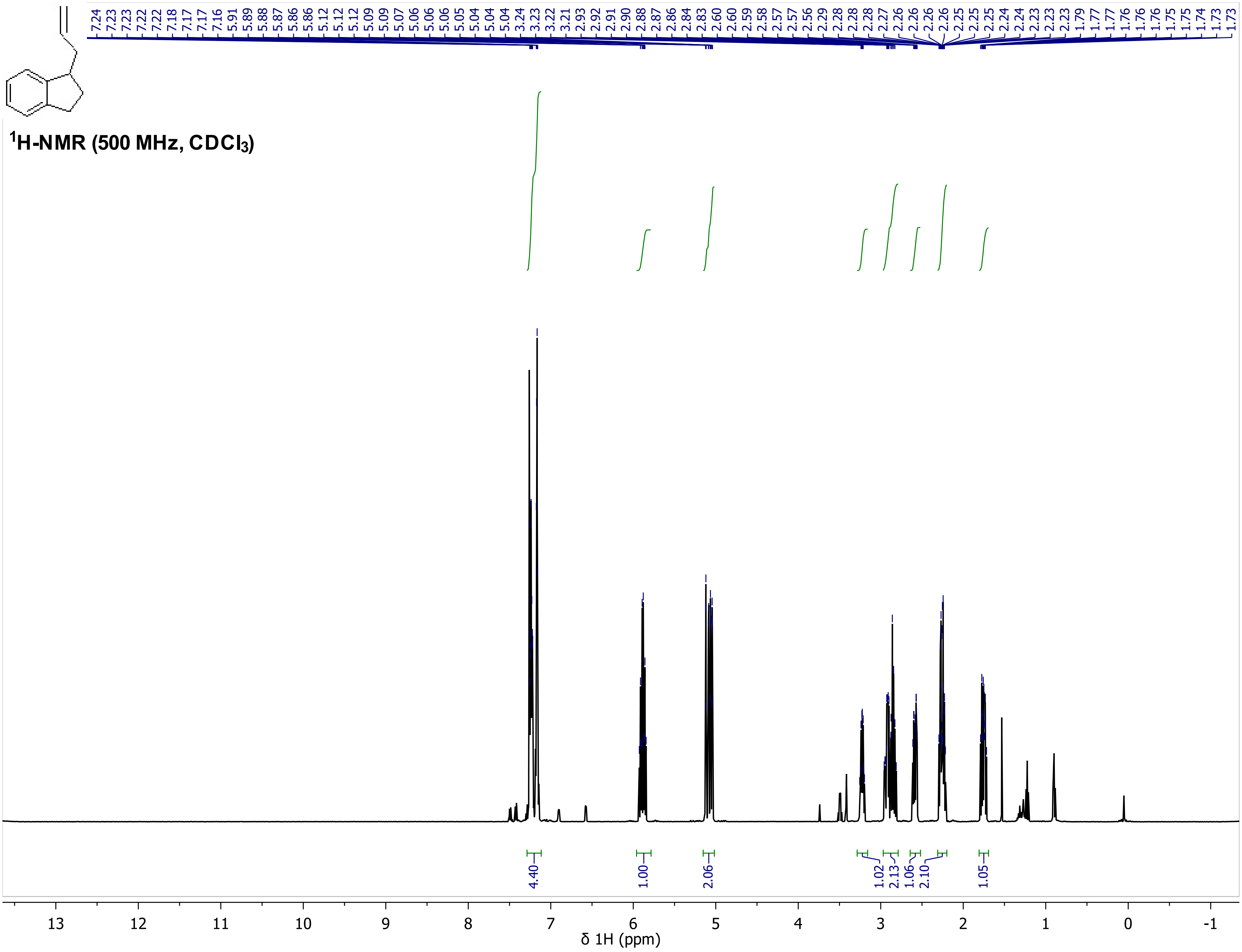

1H-NMR (500 MHz, CDCl3)
7.24 7.23 7.23 7.22 7.22 7.18 7.17 7.17 7.16 5.91 5.89 5.88 5.87 5.86 5.86 5.12 5.12 5.12 5.09 5.09 5.07 5.06 5.06 5.06 5.05 5.04 5.04 5.04 3.24 3.23 3.22 3.21 2.93 2.92 2.91 2.90 2.88 2.87 2.86 2.84 2.83 2.60 2.60 2.59 2.58 2.57 2.57 2.56 2.29 2.28 2.28 2.28 2.27 2.26 2.26 2.26 2.26 2.25 2.25 2.25 2.24 2.24 2.23 2.23 2.23 1.79 1.77 1.77 1.76 1.76 1.76 1.75 1.75 1.74 1.73 1.73
4.40
1.00
2.06
1.02
2.13
1.06
2.10
1.05
13 12 11 10 9 8 7 6 5 4 3 2 1 0 -1
δ 1H (ppm)

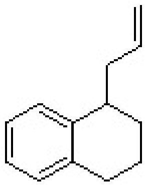

^{1}H-NMR (500 MHz, $CDCl_3$)

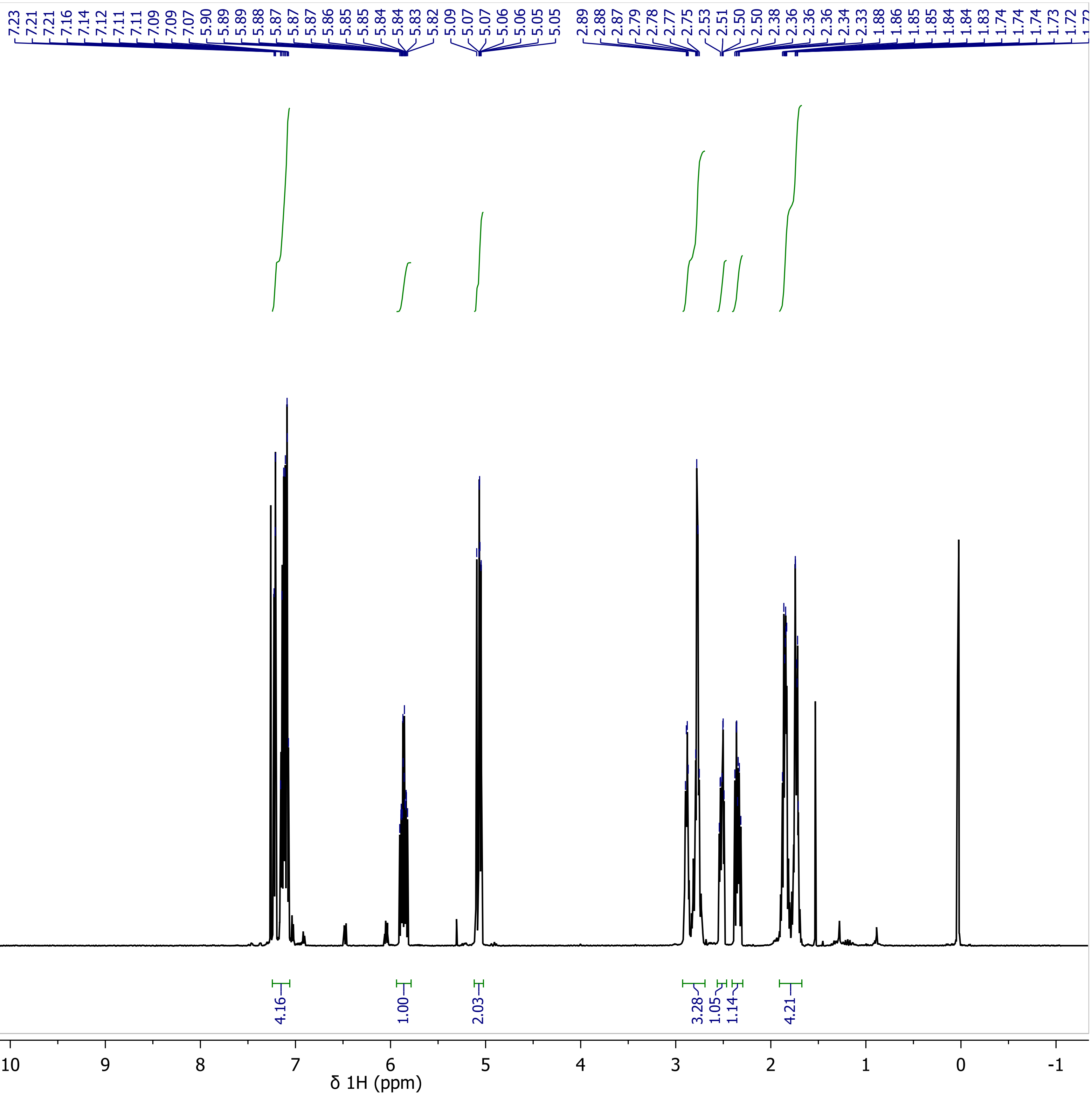

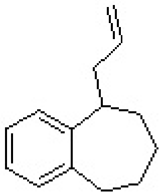

^{1}H-NMR (500 MHz, CDCl$_3$)

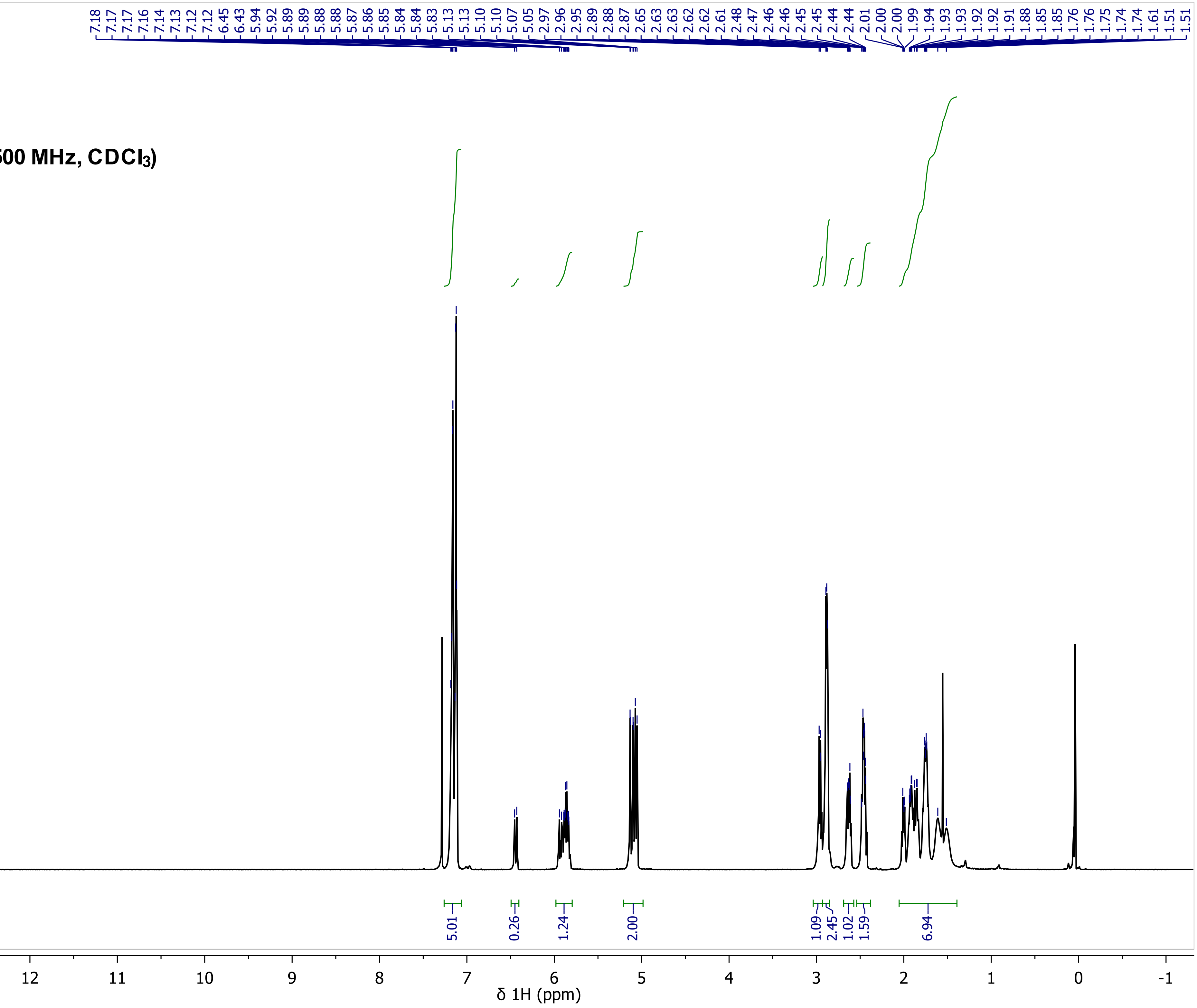

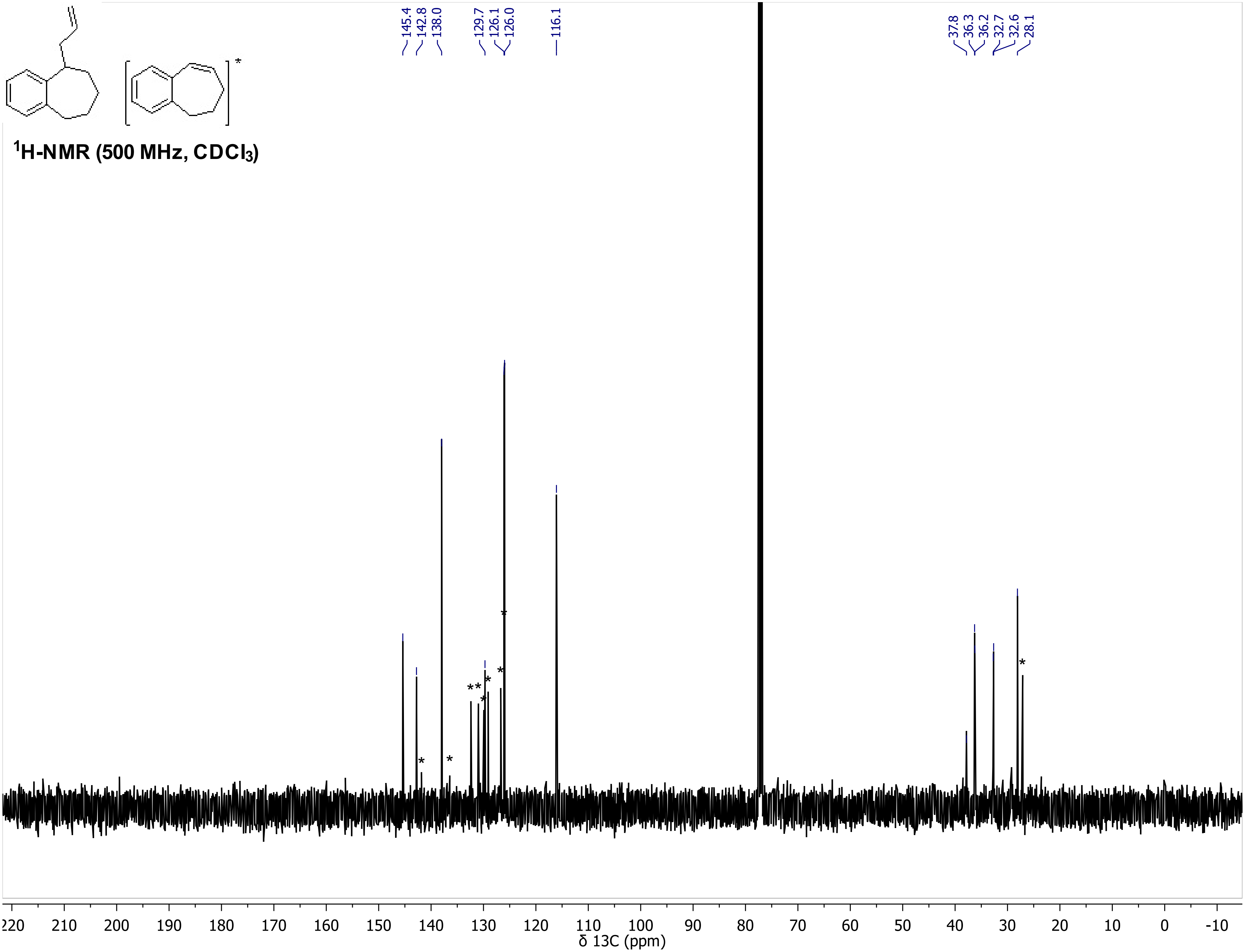
1H-NMR (500 MHz, CDCl3)
*
145.4
142.8
138.0
129.7
126.1
126.0
116.1
37.8
36.3
36.2
32.7
32.6
28.1
220 210 200 190 180 170 160 150 140 130 120 110 100 90 80 70 60 50 40 30 20 10 0 -10
δ 13C (ppm)

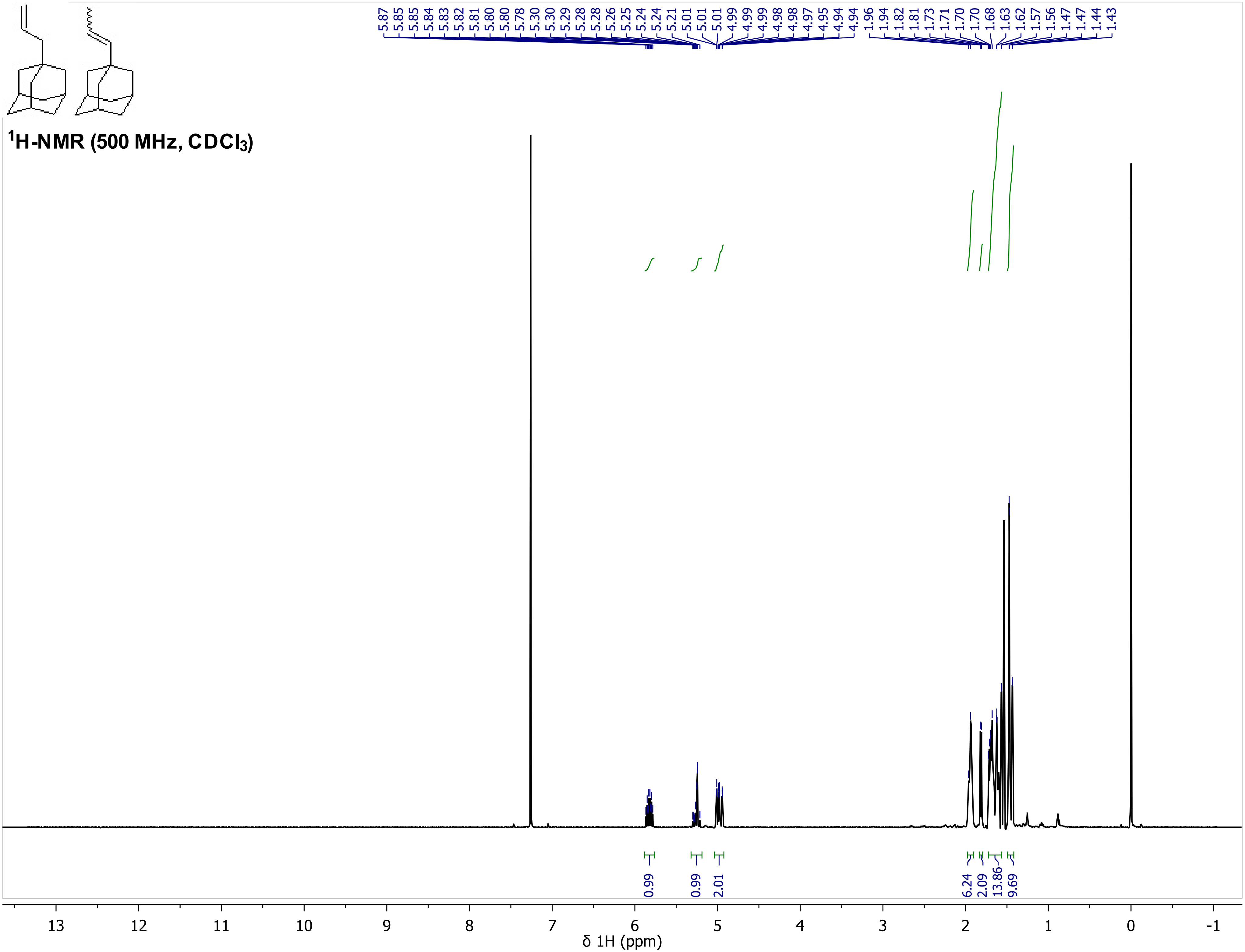

^{1}H-NMR (500 MHz, CDCl$_3$)
5.87 5.85 5.85 5.84 5.83 5.82 5.81 5.80 5.80 5.78 5.30 5.30 5.29 5.28 5.28 5.26 5.25 5.24 5.24 5.21 5.01 5.01 5.01 4.99 4.99 4.99 4.98 4.98 4.97 4.95 4.94 4.94 1.96 1.94 1.82 1.81 1.73 1.71 1.70 1.70 1.68 1.63 1.62 1.57 1.56 1.47 1.47 1.44 1.43
0.99 0.99 2.01 6.24 2.09 13.86 9.69
13 12 11 10 9 8 7 6 5 4 3 2 1 0 -1
δ 1H (ppm)

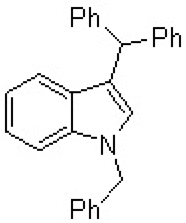

^{1}H-NMR (500 MHz, $CDCl_3$)

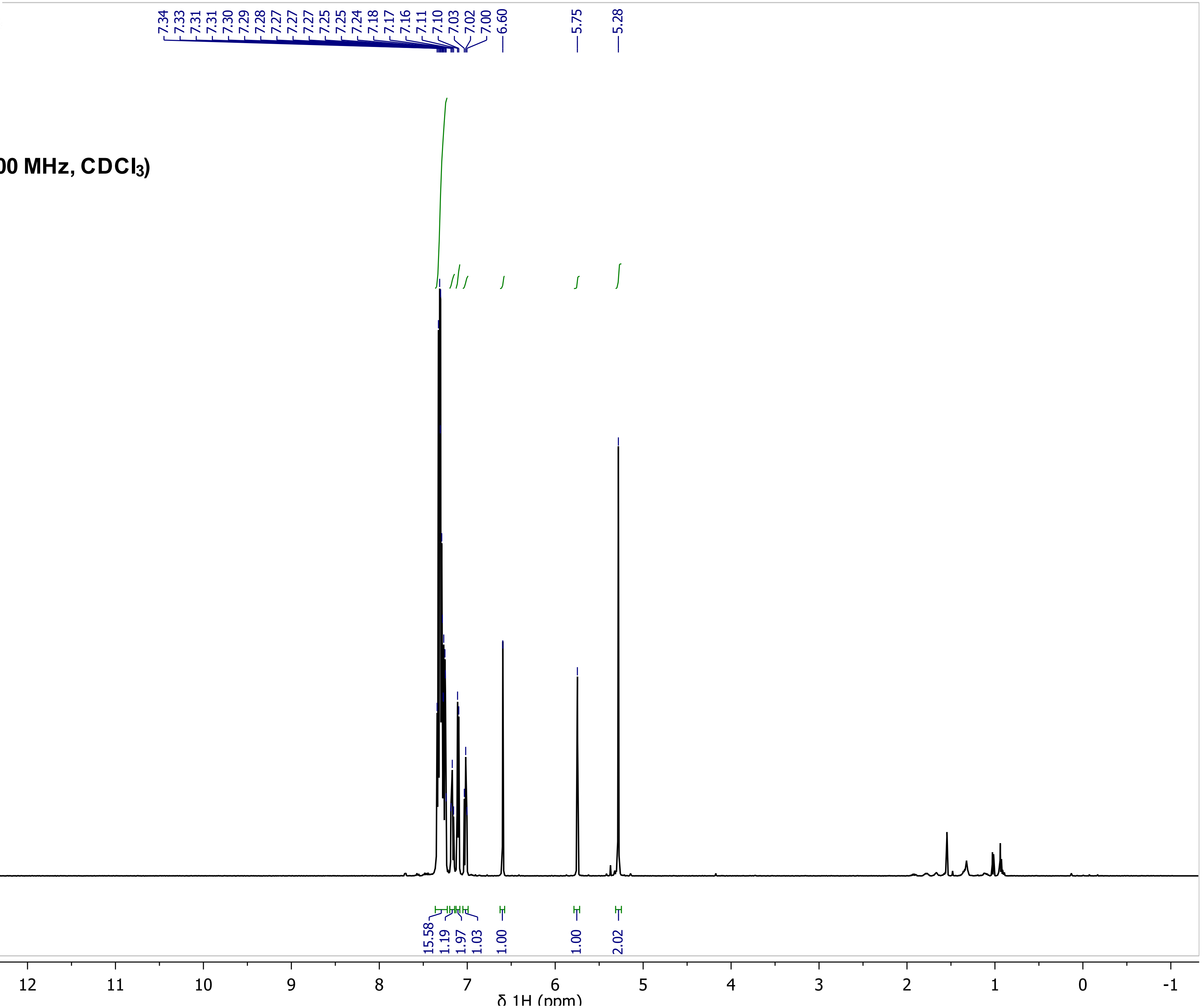

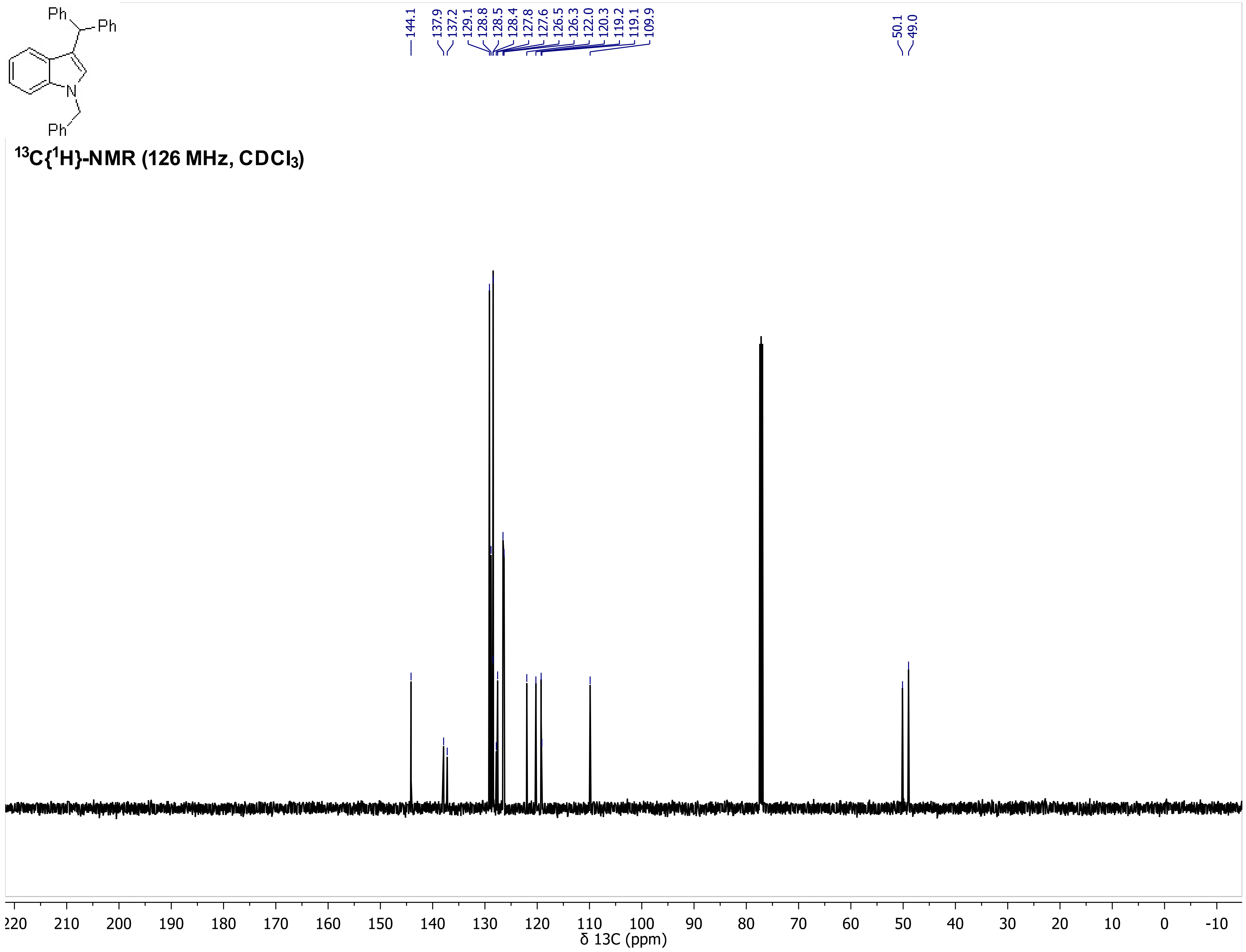

Ph
Ph
N
Ph
13C{1H}-NMR (126 MHz, CDCl3)
144.1
137.9
137.2
129.1
128.8
128.5
128.4
127.8
127.6
126.5
126.3
122.0
120.3
119.2
119.1
109.9
50.1
49.0
220 210 200 190 180 170 160 150 140 130 120 110 100 90 80 70 60 50 40 30 20 10 0 -10
δ 13C (ppm)

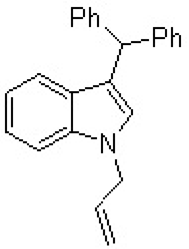

^{1}H-NMR (500 MHz, $CDCl_3$)

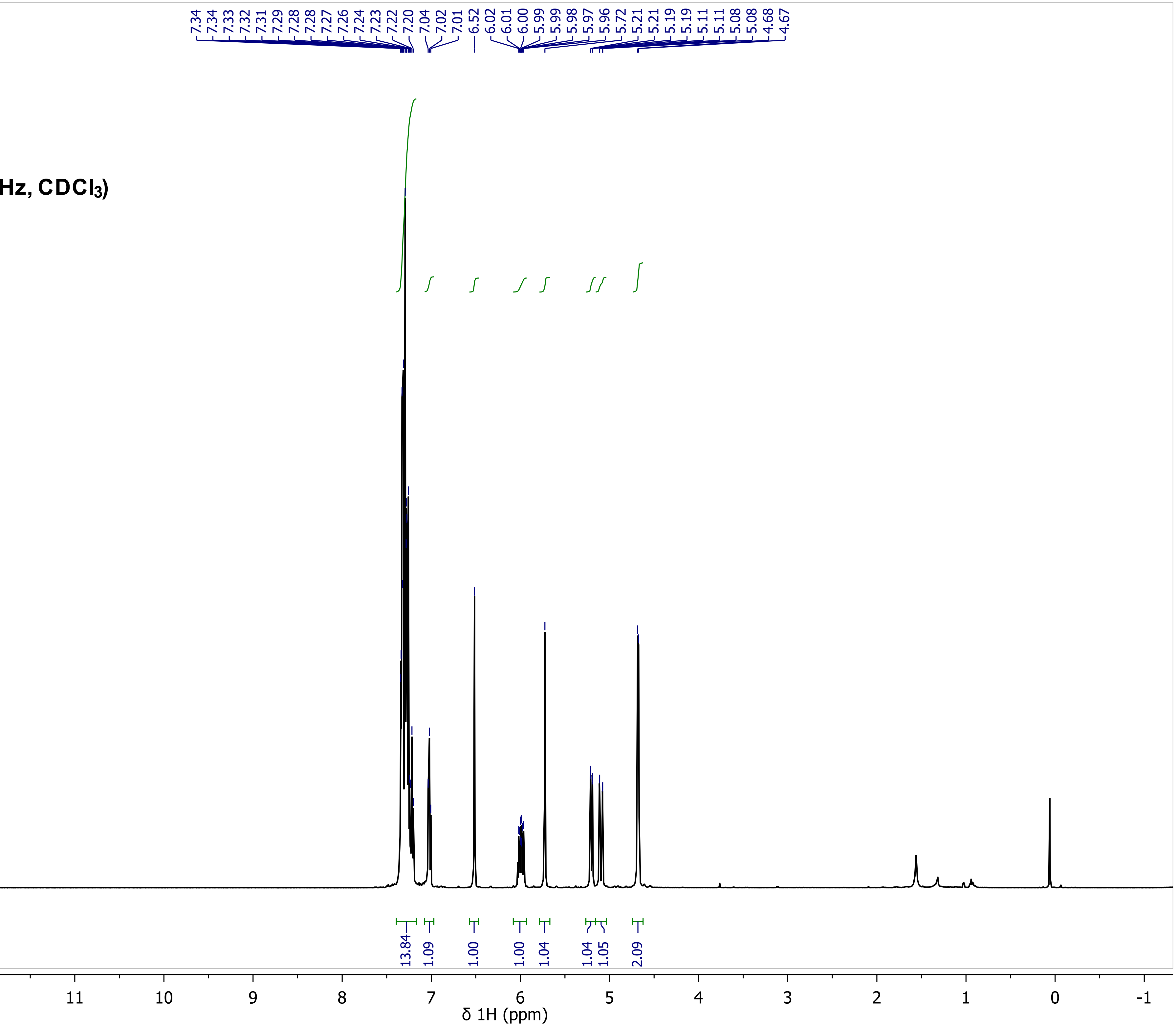

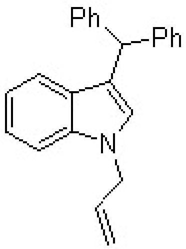

^{1}H-NMR (500 MHz, $CDCl_3$)

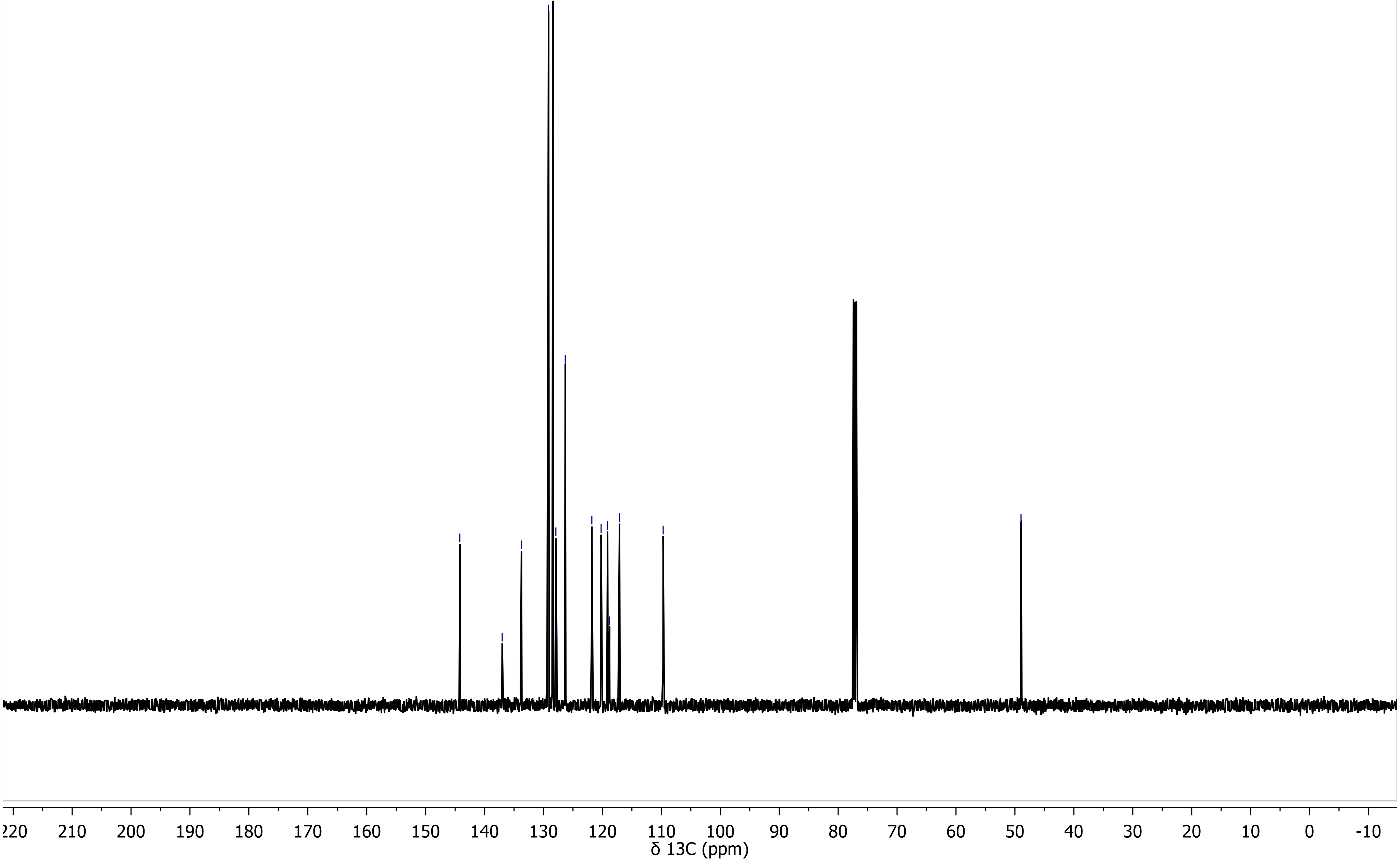

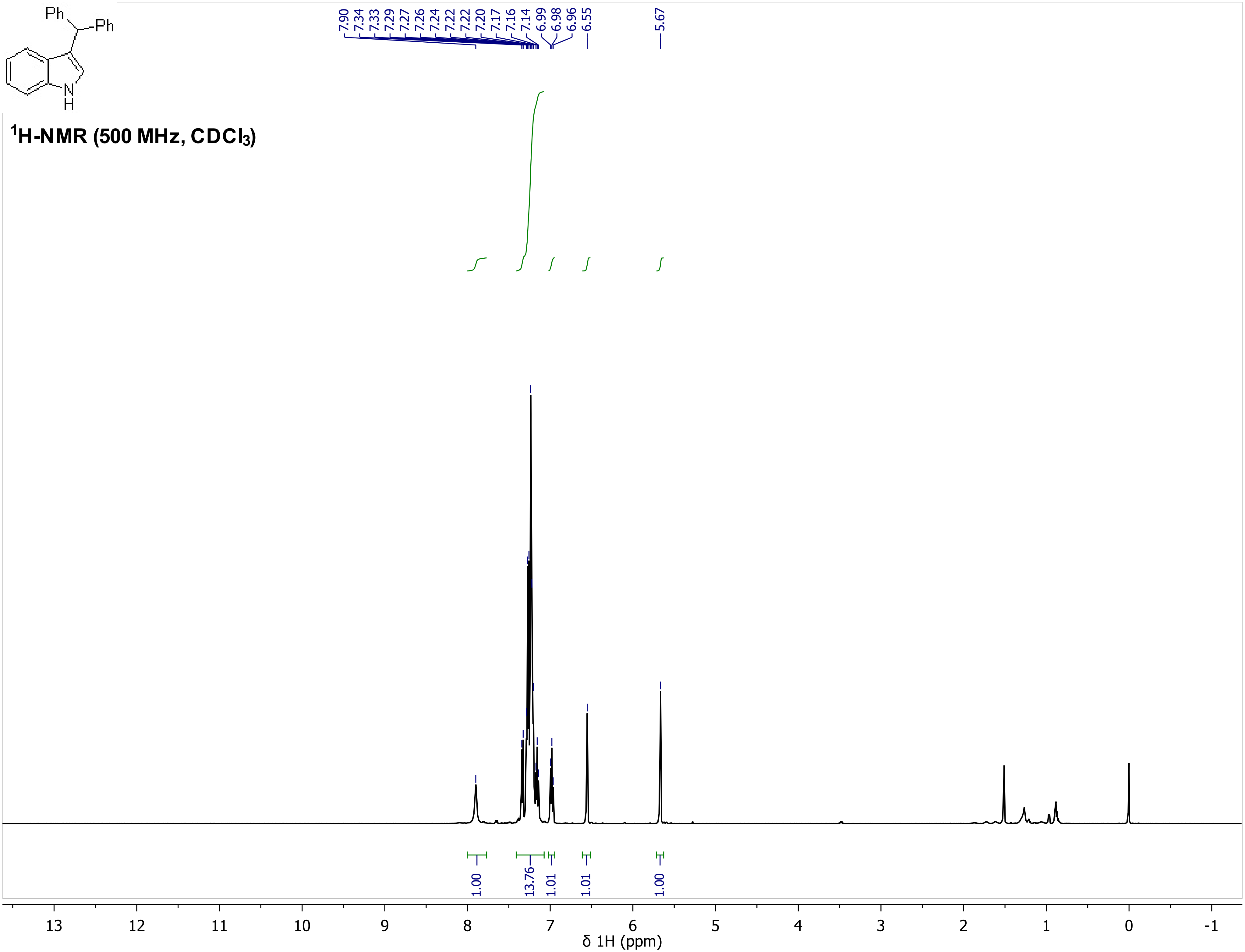
Ph
Ph
N
H
1H-NMR (500 MHz, CDCl3)
7.90
7.34
7.33
7.29
7.27
7.26
7.24
7.22
7.22
7.20
7.17
7.16
7.14
6.99
6.98
6.96
6.55
5.67
1.00
13.76
1.01
1.01
1.00
13
12
11
10
9
8
7
6
5
4
3
2
1
0
-1
δ 1H (ppm)

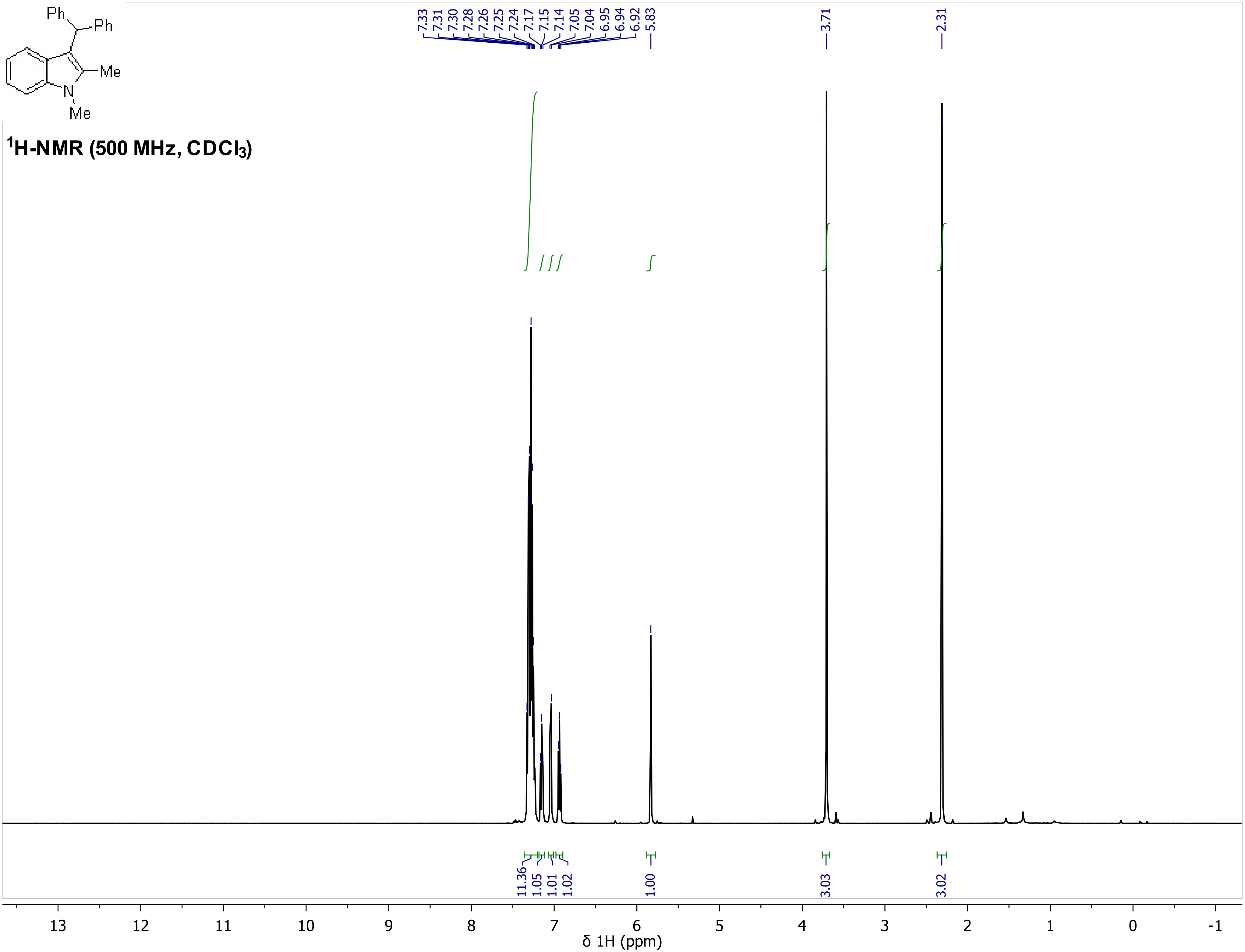

^{1}H-NMR (500 MHz, $CDCl_3$)

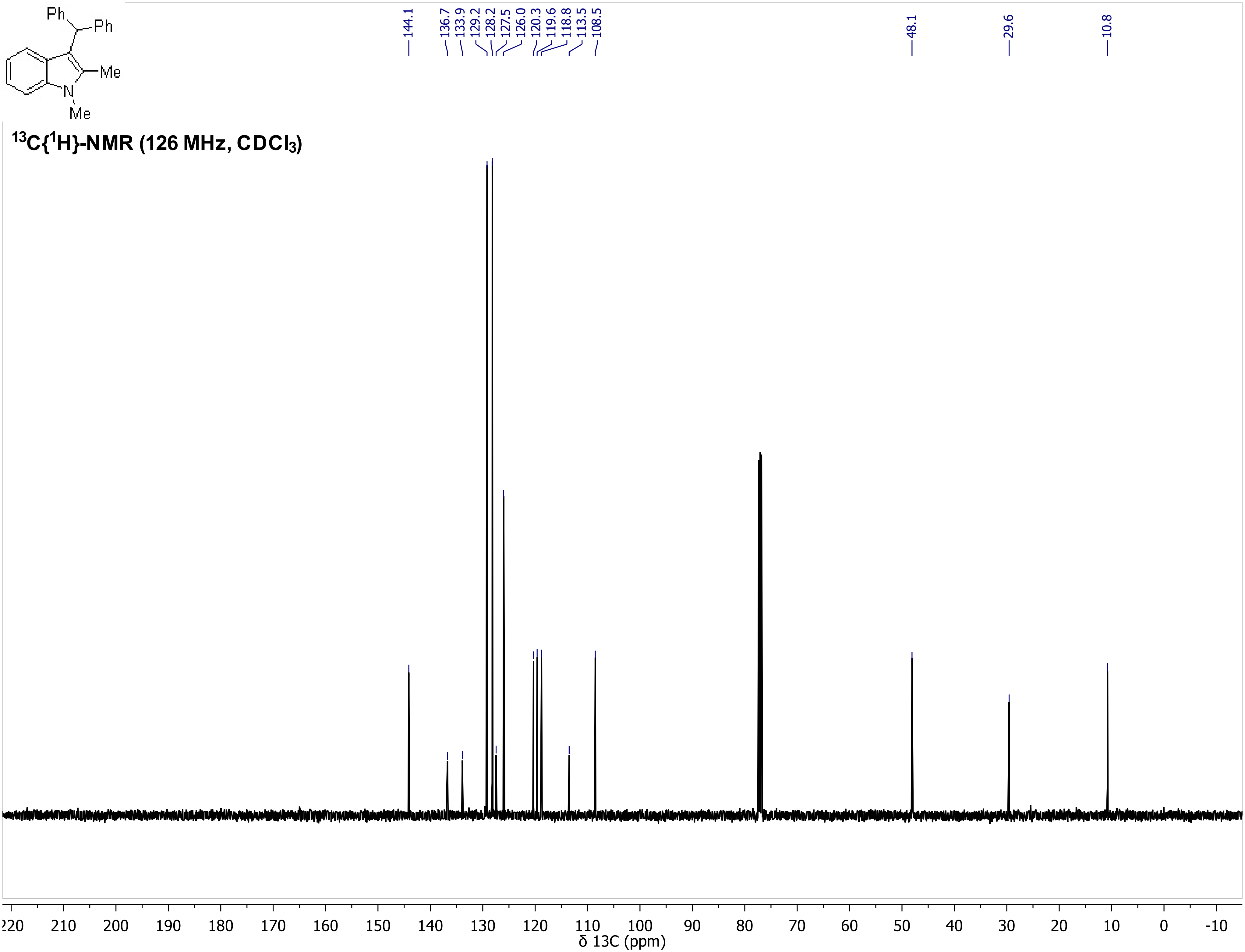
Ph
Ph
Me
N
Me
$^{13}C\{^1H\}$-NMR (126 MHz, $CDCl_3$)
144.1
136.7
133.9
129.2
128.2
127.5
126.0
120.3
119.6
118.8
113.5
108.5
48.1
29.6
10.8
220
210
200
190
180
170
160
150
140
130
120
110
100
90
80
70
60
50
40
30
20
10
0
-10
δ 13C (ppm)

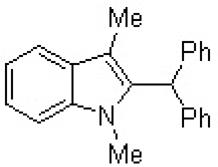

^{1}H-NMR (500 MHz, $CDCl_3$)

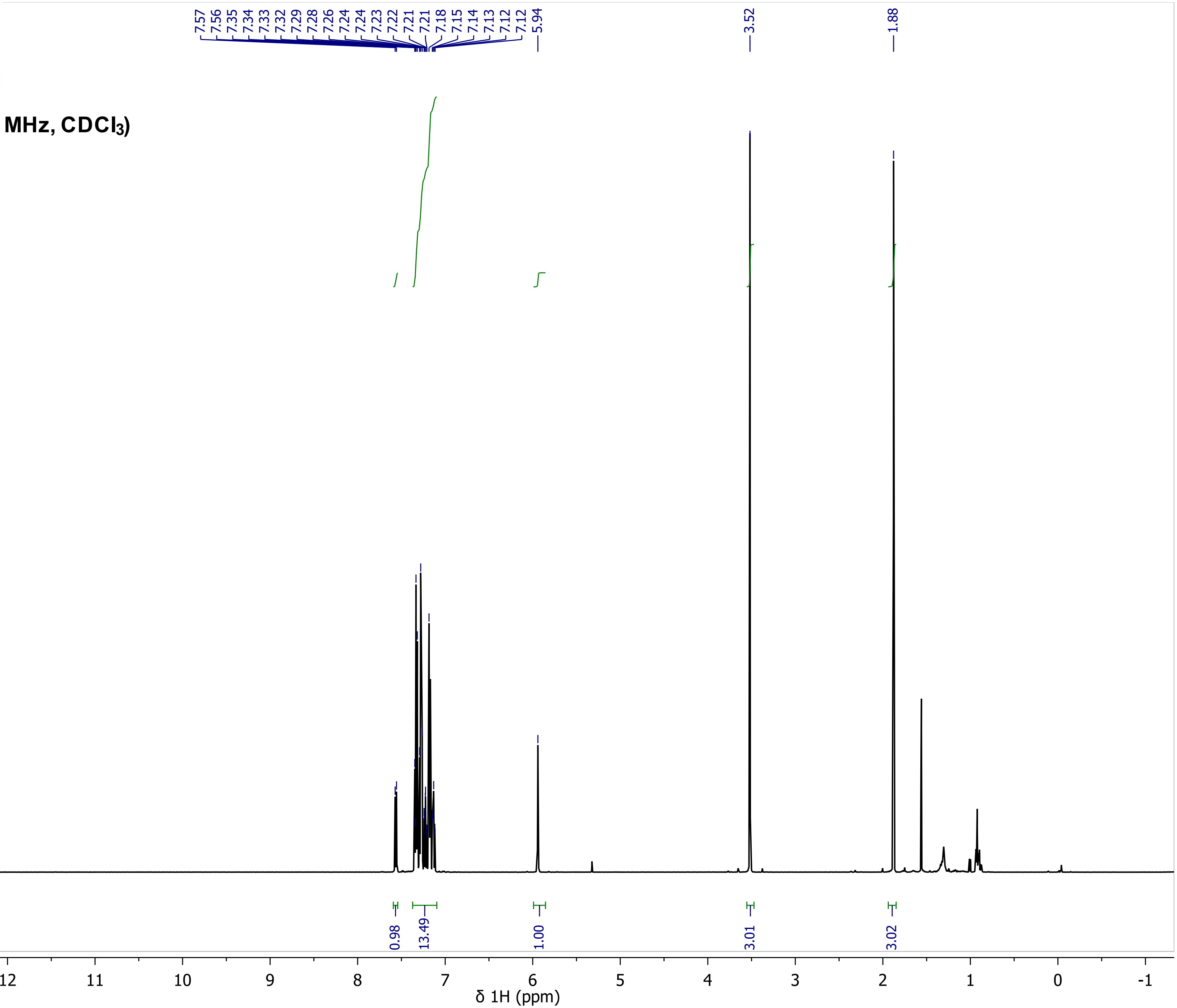

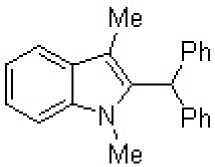

$^{13}C\{^1H\}$-NMR (126 MHz, $CDCl_3$)

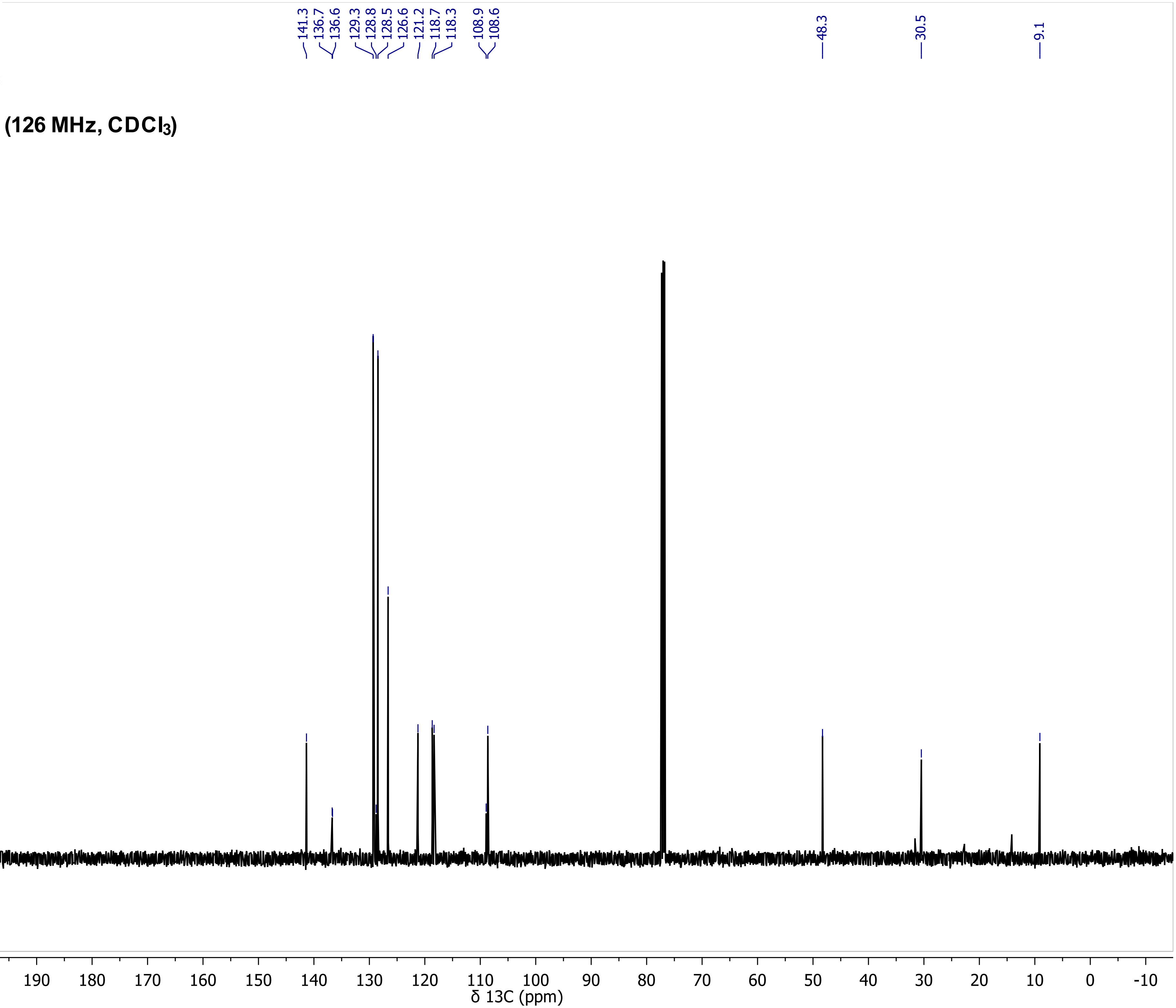

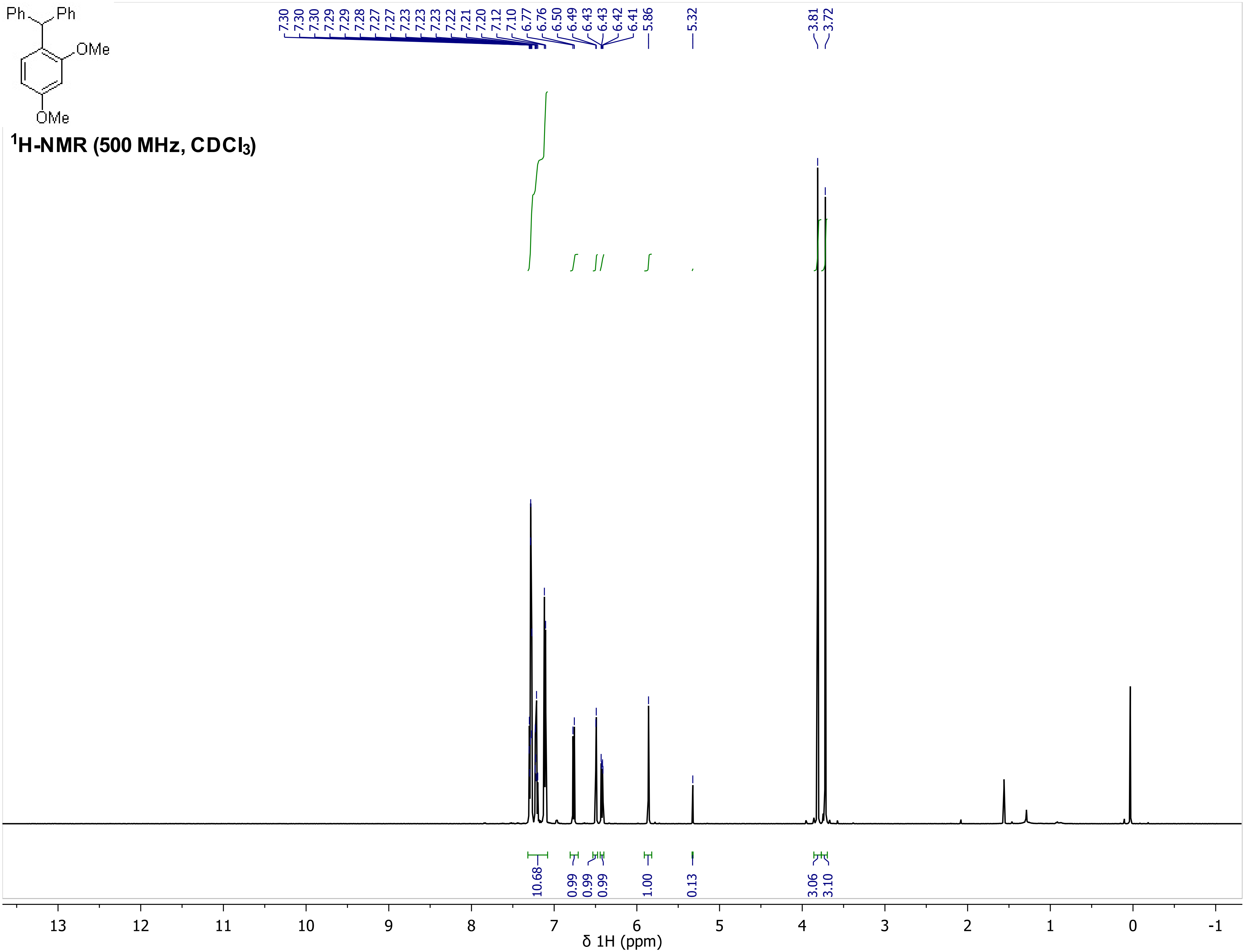

Ph
Ph
OMe
OMe
1H-NMR (500 MHz, CDCl3)
7.30
7.30
7.30
7.29
7.29
7.28
7.27
7.27
7.23
7.23
7.23
7.22
7.21
7.20
7.12
7.10
6.77
6.76
6.50
6.49
6.43
6.43
6.42
6.41
5.86
5.32
3.81
3.72
10.68
0.99
0.99
0.99
1.00
0.13
3.06
3.10
13
12
11
10
9
8
7
6
5
4
3
2
1
0
-1
δ 1H (ppm)

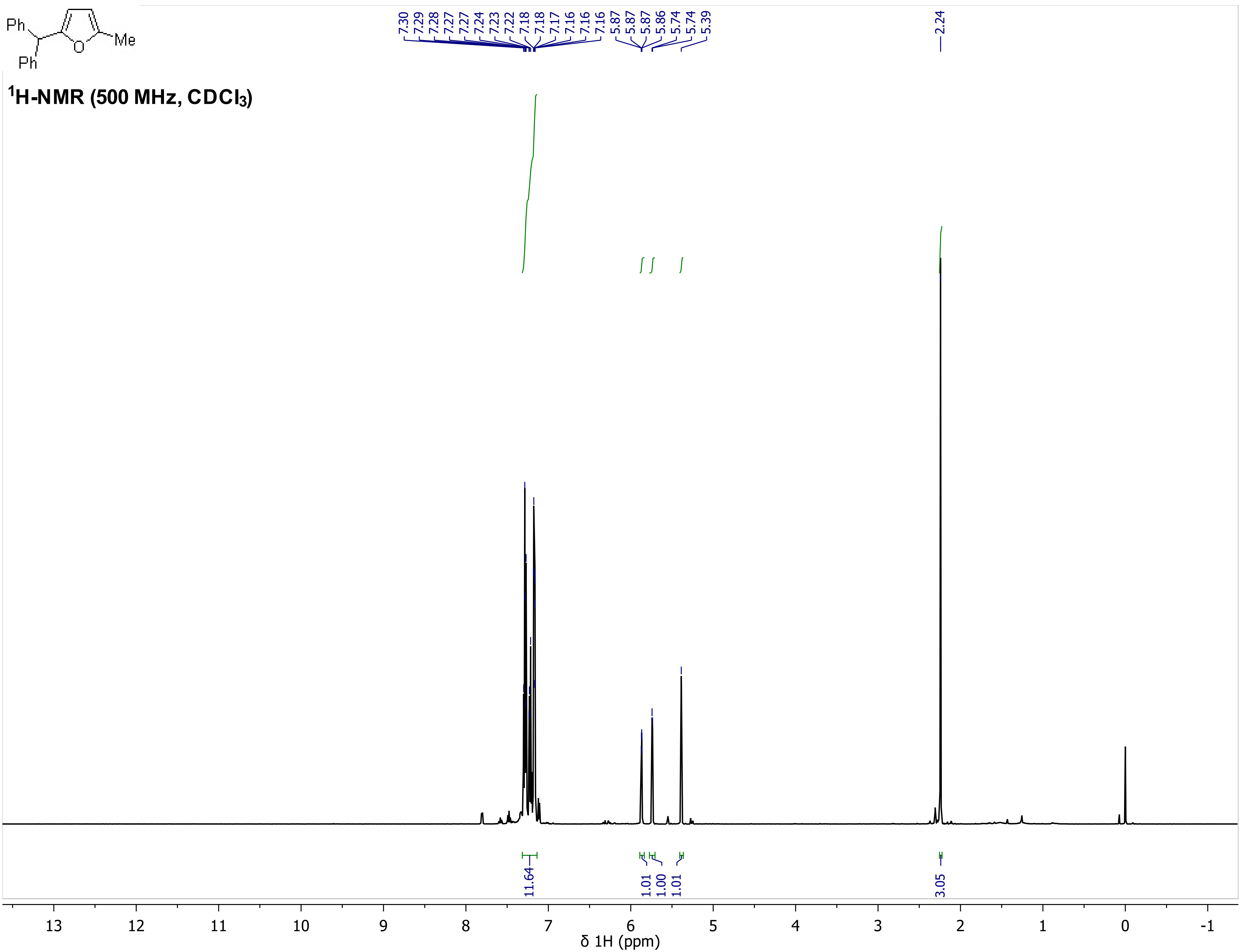

Ph
Ph
O
Me
¹H-NMR (500 MHz, CDCl3)
7.30
7.29
7.28
7.27
7.27
7.24
7.23
7.22
7.18
7.18
7.17
7.16
7.16
7.16
5.87
5.87
5.87
5.86
5.74
5.74
5.39
2.24
11.64
1.01
1.00
1.01
3.05
13
12
11
10
9
8
7
6
5
4
3
2
1
0
-1
δ 1H (ppm)

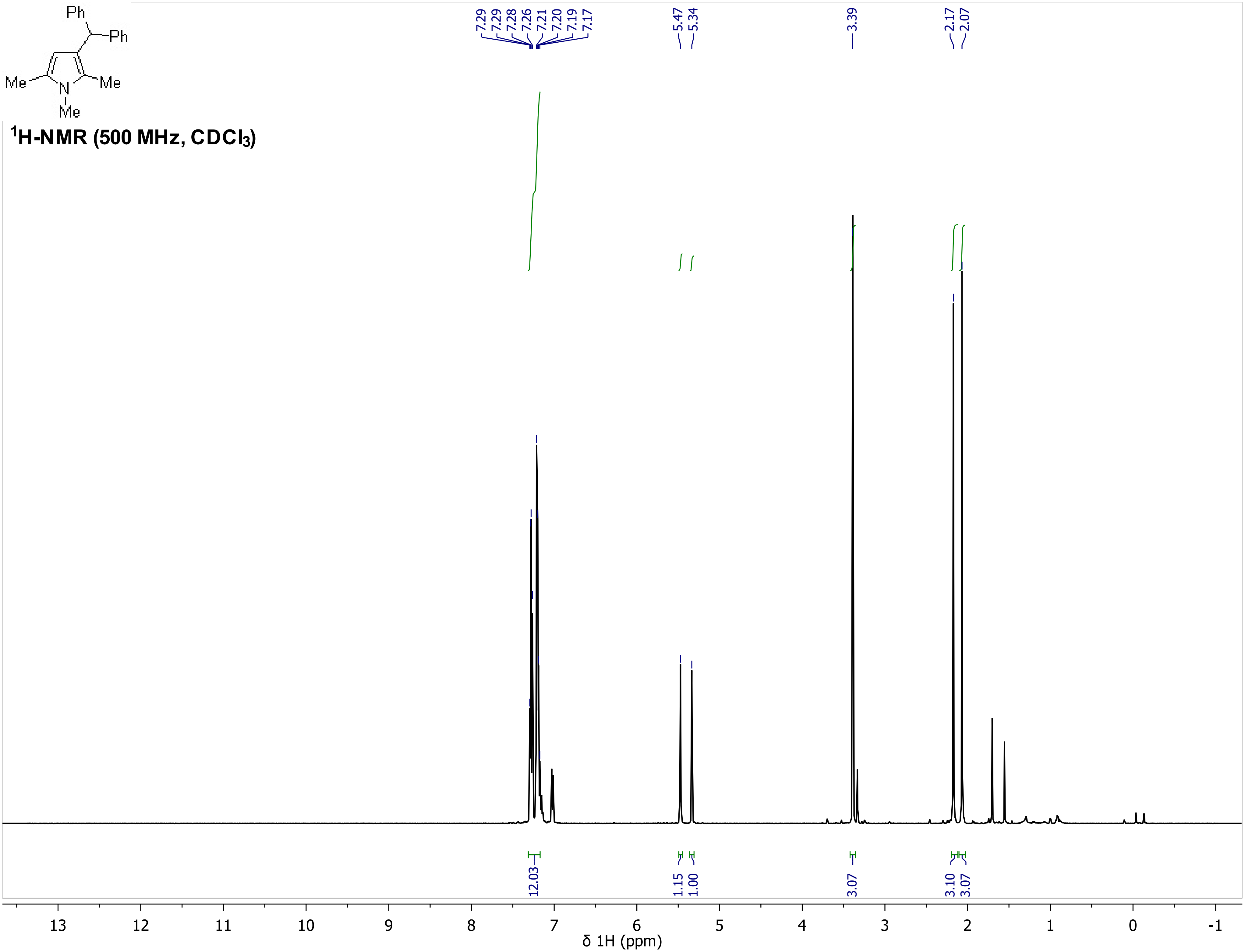

Ph
Ph
Me
N
Me
Me
¹H-NMR (500 MHz, CDCl₃)
7.29
7.29
7.28
7.26
7.21
7.20
7.19
7.17
5.47
5.34
3.39
2.17
2.07
12.03
1.15
1.00
3.07
3.10
3.07
13
12
11
10
9
8
7
6
5
4
3
2
1
0
-1
δ 1H (ppm)

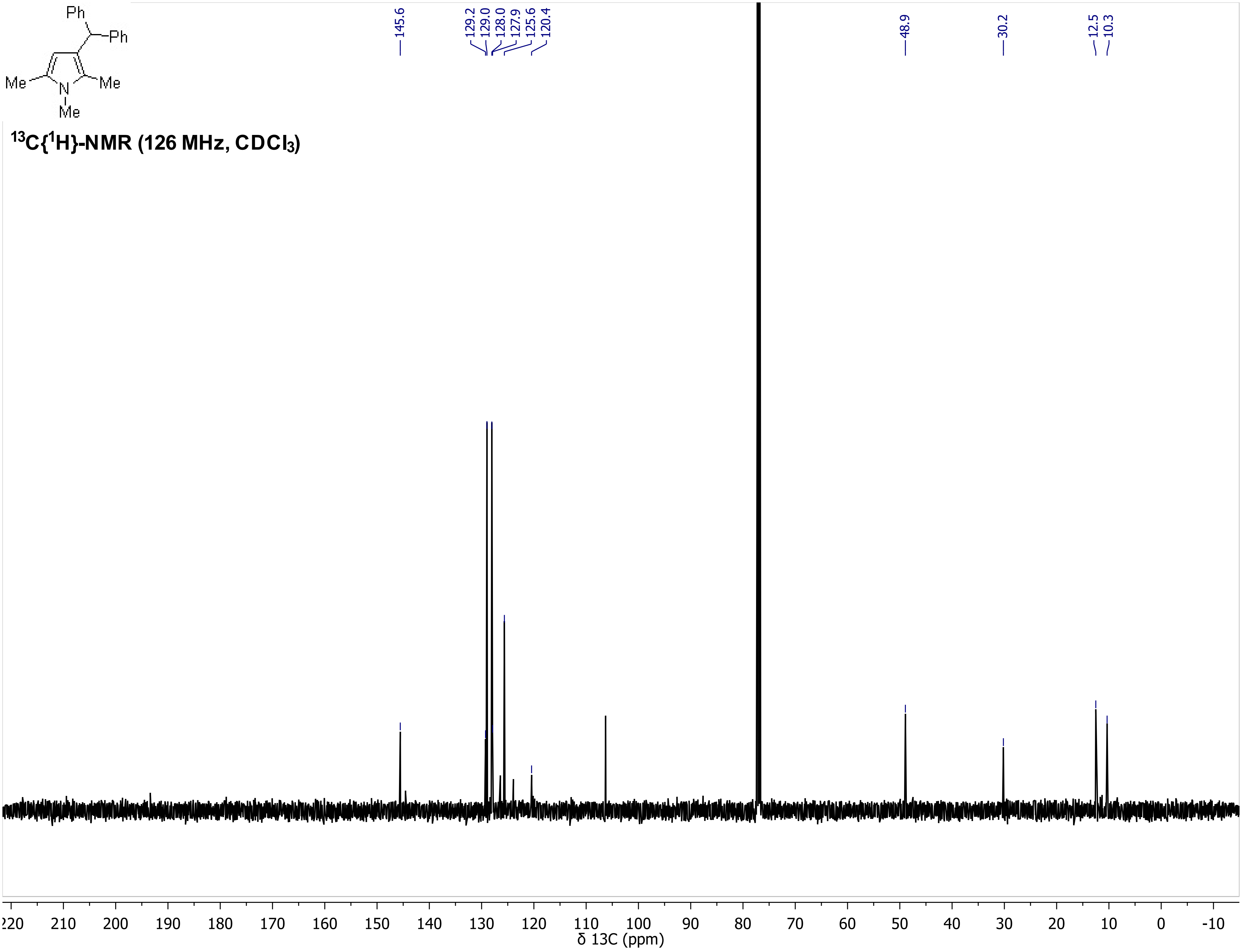

Ph
Ph
Me
Me
N
Me
$^{13}C\{^{1}H\}$-NMR (126 MHz, $CDCl_3$)
145.6
129.2
129.0
128.0
127.9
125.6
120.4
48.9
30.2
12.5
10.3
220 210 200 190 180 170 160 150 140 130 120 110 100 90 80 70 60 50 40 30 20 10 0 -10
δ 13C (ppm)

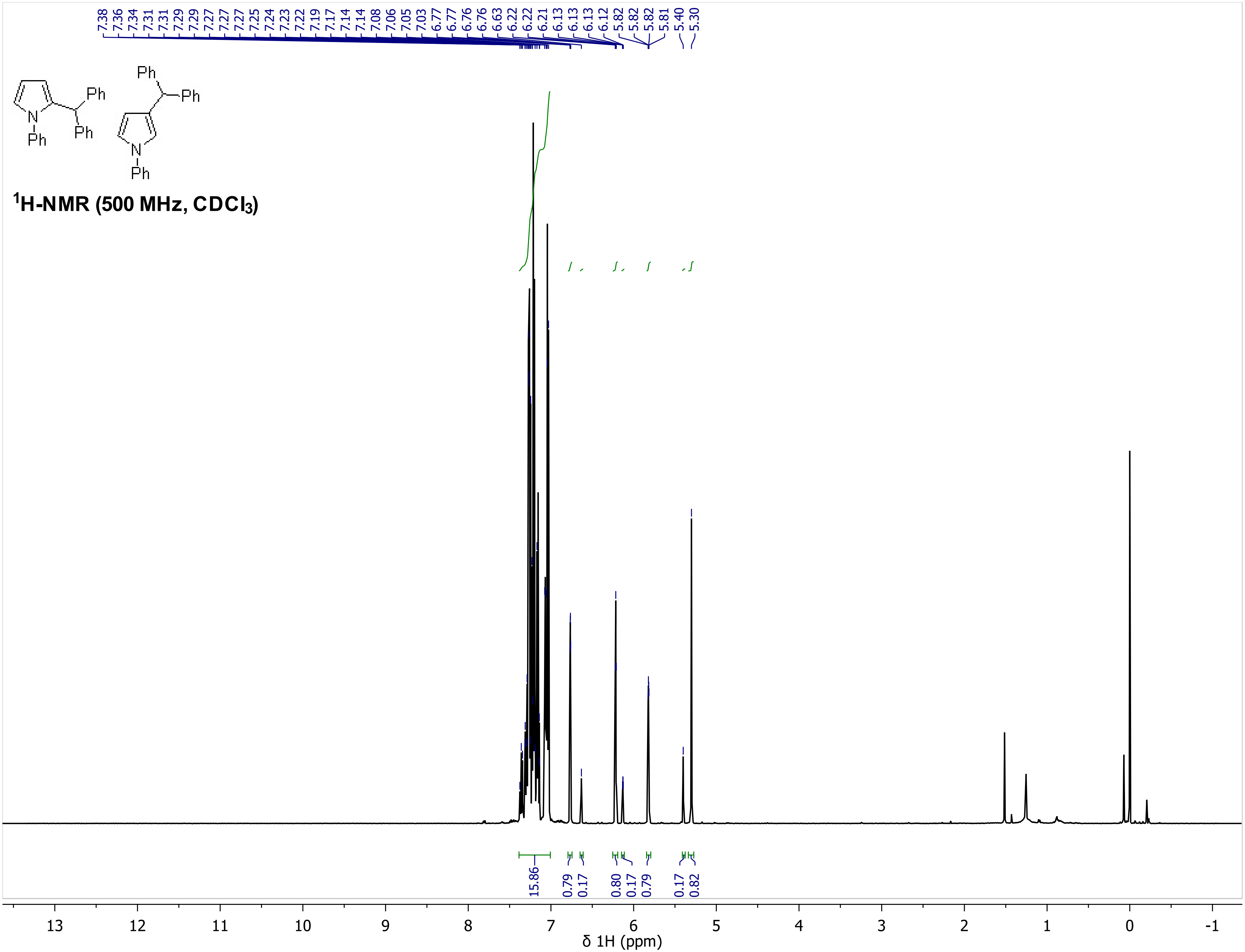

7.38
7.36
7.34
7.31
7.31
7.29
7.29
7.27
7.27
7.27
7.25
7.24
7.23
7.22
7.19
7.17
7.14
7.14
7.08
7.06
7.05
7.03
6.77
6.77
6.76
6.76
6.63
6.22
6.22
6.21
6.13
6.13
6.13
6.12
5.82
5.82
5.82
5.81
5.40
5.30
Ph
Ph
N
Ph
Ph
Ph
N
Ph
1H-NMR (500 MHz, CDCl3)
15.86
0.79
0.17
0.80
0.17
0.79
0.17
0.82
13
12
11
10
9
8
7
6
5
4
3
2
1
0
-1
δ 1H (ppm)

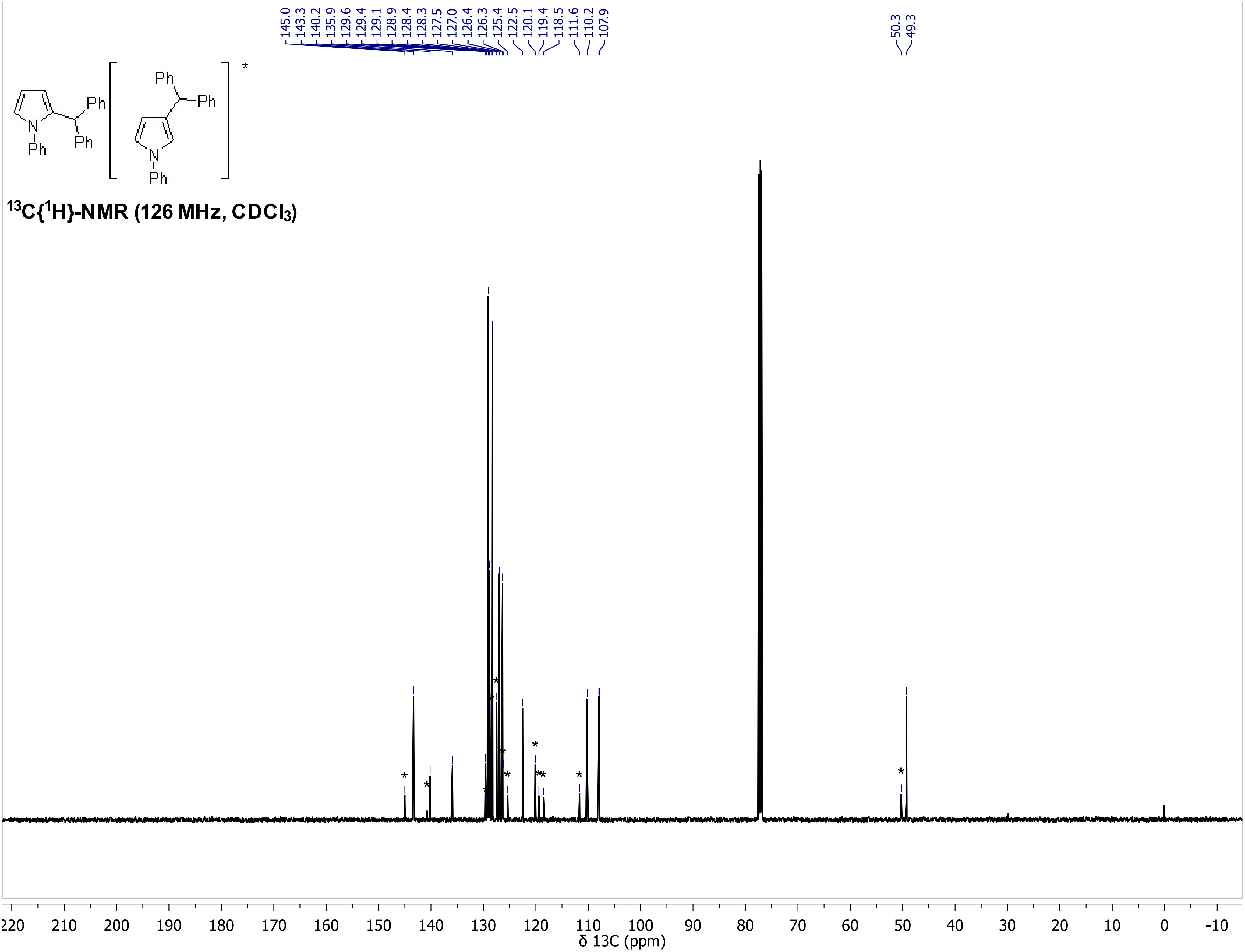

$^{13}C\{^1H\}$-NMR (126 MHz, $CDCl_3$)
